# Islands of retroelements are major components of *Drosophila* centromeres

**DOI:** 10.1371/journal.pbio.3000241

**Published:** 2019-05-14

**Authors:** Ching-Ho Chang, Ankita Chavan, Jason Palladino, Xiaolu Wei, Nuno M. C. Martins, Bryce Santinello, Chin-Chi Chen, Jelena Erceg, Brian J. Beliveau, Chao-Ting Wu, Amanda M. Larracuente, Barbara G. Mellone

**Affiliations:** 1 Department of Biology, University of Rochester; Rochester, New York, United States of America; 2 Department of Molecular and Cell Biology, University of Connecticut, Storrs, Connecticut, United States of America; 3 Department of Biomedical Genetics, University of Rochester Medical Center, Rochester, New York, United States of America; 4 Department of Genetics, Harvard Medical School, Boston, Massachusetts, United States of America; 5 Wyss Institute for Biologically Inspired Engineering, Harvard Medical School, Boston, Massachusetts, United States of America; 6 Department of Systems Biology, Harvard Medical School, Boston, Massachusetts, United States of America; 7 Department of Genome Sciences, University of Washington Seattle, Seattle, Washington, United States of America; 8 Institute for Systems Genomics, University of Connecticut Storrs, Connecticut, United States of America; Biomedical Center Munich, GERMANY

## Abstract

Centromeres are essential chromosomal regions that mediate kinetochore assembly and spindle attachments during cell division. Despite their functional conservation, centromeres are among the most rapidly evolving genomic regions and can shape karyotype evolution and speciation across taxa. Although significant progress has been made in identifying centromere-associated proteins, the highly repetitive centromeres of metazoans have been refractory to DNA sequencing and assembly, leaving large gaps in our understanding of their functional organization and evolution. Here, we identify the sequence composition and organization of the centromeres of *Drosophila melanogaster* by combining long-read sequencing, chromatin immunoprecipitation for the centromeric histone CENP-A, and high-resolution chromatin fiber imaging. Contrary to previous models that heralded satellite repeats as the major functional components, we demonstrate that functional centromeres form on islands of complex DNA sequences enriched in retroelements that are flanked by large arrays of satellite repeats. Each centromere displays distinct size and arrangement of its DNA elements but is similar in composition overall. We discover that a specific retroelement, *G2/Jockey-3*, is the most highly enriched sequence in CENP-A chromatin and is the only element shared among all centromeres. *G2/Jockey-3* is also associated with CENP-A in the sister species *D*. *simulans*, revealing an unexpected conservation despite the reported turnover of centromeric satellite DNA. Our work reveals the DNA sequence identity of the active centromeres of a premier model organism and implicates retroelements as conserved features of centromeric DNA.

## Abstract 2

Long-read sequencing, CENP-A ChIP, and chromatin fiber imaging reveal the composition and organization of *Drosophila melanogaster* centromeres, which have long remained elusive despite the high quality of this species’ genome. assembly.

## Introduction

Centromeres are marked by the histone H3 variant centromere protein A (CENP-A; also called centromere identifier [Cid] in *Drosophila* and centromeric histone H3 [CenH3] in plants), which is necessary and sufficient for kinetochore activity [1, 2]. Although epigenetic mechanisms play a major role in centromere identity and propagation [3], centromeric DNA sequences can initiate centromere assembly in fission yeast [4] and humans [5], and centromeric transcripts play a role in centromere propagation in human cells [6], suggesting that centromeric DNA-encoded properties may contribute to centromere specification [7]. However, our current understanding of most centromeres remains at the cytological level, as metazoan centromeres are embedded in highly repetitive, satellite-rich pericentric heterochromatin and thus are largely missing from even the most complete genome assemblies. Only recently, long-read single molecule sequencing technologies have made it possible to obtain linear assemblies of highly repetitive parts of multicellular genomes such as the human Y chromosome centromere [8] and maize centromere 10 [9].

*Drosophila melanogaster* provides an ideal model to investigate centromere genomic organization, as it has a relatively small genome (roughly 180 Mb), organized in just three autosomes (chromosome 2, 3, and 4) and two sex chromosomes (X and Y) [10]. The estimated centromere sizes in *Drosophila* cultured cells range between approximately 200 and 500 kb [11] and map to regions within large blocks of tandem repeats [12–15]. While CENP-A associates with simple satellites in chromatin immunoprecipitation sequencing (ChIP-seq) data [16], it may bind to additional undiscovered sequences. The linear organization at the sequence level of any of the centromeres is unknown in this species. Early efforts to determine the structural organization of centromeres in *D*. *melanogaster* combined deletion analyses and sequencing of an X-derived minichromosome, *Dp1187*. These studies mapped the minimal DNA sequences sufficient for centromere function to a 420-kb region containing the AAGAG and AATAT satellites interspersed with “islands” of complex sequences [14, 15]. However, it is unclear which parts of this minimal region comprise the active centromere, whether it corresponds to the native X chromosome centromere, and if other centromeres have a similar organization. By and large, satellites have been regarded as the major structural elements of *Drosophila*, humans, and mouse centromeres [2, 3, 17].

In this study, we reveal the detailed organization of all functional centromeres in *D*. *melanogaster*. By mapping CENP-A on single chromatin fibers at high resolution, we discover that CENP-A primarily occupies islands of complex DNA enriched in retroelements, which are flanked by large blocks of simple satellites. Our genomic analyses show that all centromeres have a unique sequence organization, even though many of the centromeric elements are shared among centromeres. In particular, all centromeres are enriched for a non–long terminal repeat (non-LTR) retroelement in the *Jockey* family, *G2/Jockey-3*. Although none of these elements are specific to centromeres, they are significantly enriched within these regions. We also find *G2/Jockey-3* enriched at the centromeres of *D*. *simulans*, which has centromeric satellite arrays highly divergent from those of *D*. *melanogaster* [16]. Collectively, these data are consistent with the model that retroelements may have a conserved role in centromere specification and function, as proposed for other species (for review, see [18]).

## Results

### Identification of candidate centromeres by long-read sequencing and ChIP-seq

**Fig 1:**
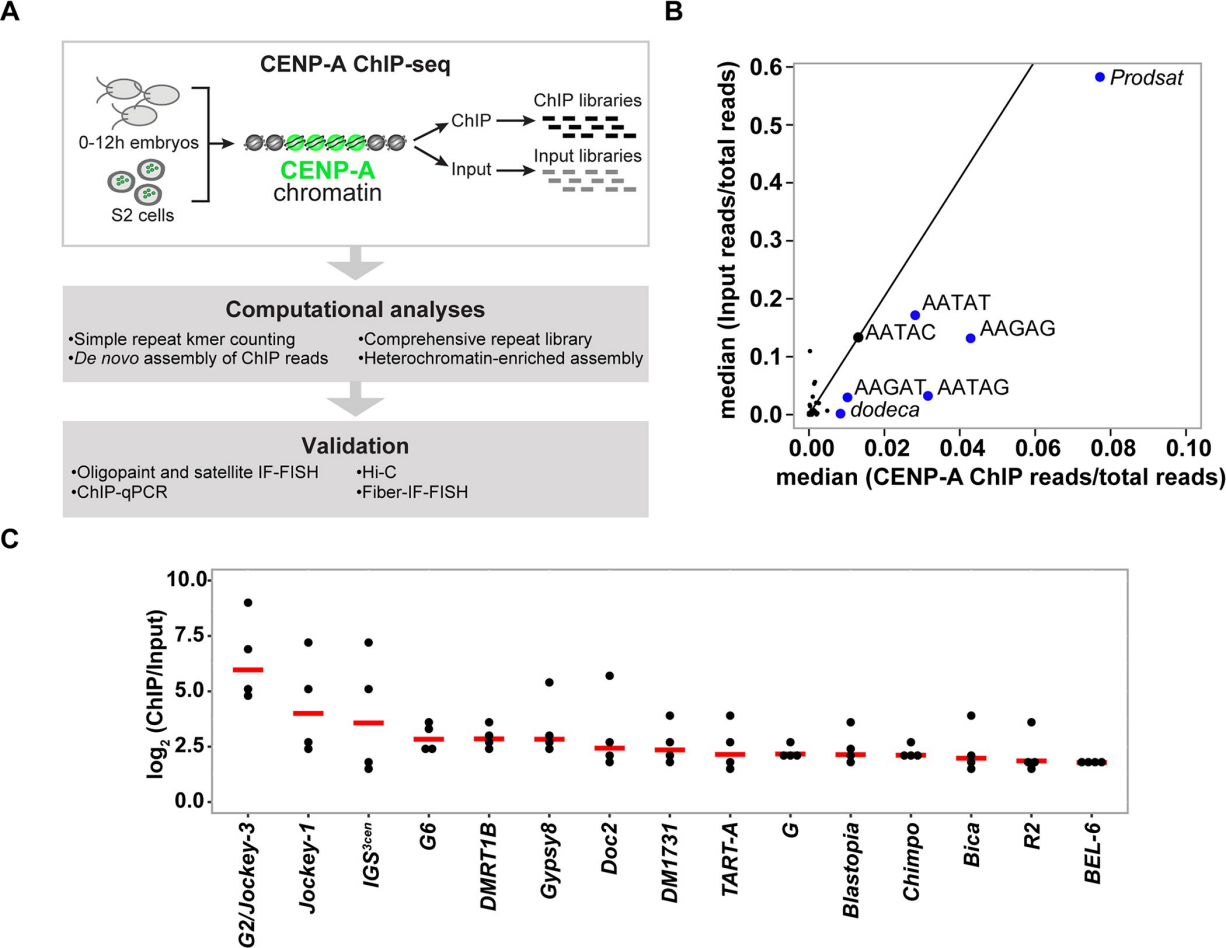
CENP-A binding association with satellites and transposable elements. (A) Schematic of the strategy used to identify the DNA sequence of *D*. *melanogaster* centromeres. The Illumina reads are 2 × 150 bp. (B) Kseek plot showing the relative abundance of simple repeat sequences in CENP-A ChIP compared to the input. Plotted on the x-axis is the median of CENP-A ChIP reads normalized over total mapped CENP-A ChIP reads across four ChIP replicates. Plotted on the y-axis is the median of input reads normalized over total mapped input reads across four replicates. The top 7 kmers in the ChIP read abundance are labeled. The line represents the enrichment of CENP-A ChIP/input for AATAC, a noncentromeric simple repeat. Repeats to the right of the line are putatively enriched in CENP-A. See also S1 Fig and S1 Table. (C) Plot of the normalized CENP-A/input reads on a log scale for each replicate, sorted by median (red lines) for complex repeat families. Shown are only the complex repeats in the top 20% across all four CENP-A ChIP replicates. See also S2 Fig and S2 Table. CENP-A, centromere protein A; ChIP, chromatin immunoprecipitation; ChIP-seq, ChIP sequencing; IF-FISH, immunofluorescence–fluorescence in situ hybridization; IGS^3cen^, intergenic spacer of the ribosomal genes on the third centromere; *Prodsat*, Prod satellite; qPCR, quantitative PCR; S2, Schneider 2; *TART*, Telomere-associated retrotransposon.

To identify the centromeric DNA sequences of *D*. *melanogaster*, we combined a long-read genome assembly approach [19] with four replicate CENP-A ChIPs on chromatin from *D*. *melanogaster* embryos, followed by paired-end Illumina sequencing (ChIP-seq). We also performed ChIP-seq in *D*. *melanogaster* Schneider 2 (S2) cells, a widely used model for cell division studies. We took four complementary approaches to discover regions of the genome enriched for CENP-A: (1) identifying simple repeats enriched for CENP-A based on kmers, (2) mapping reads to a comprehensive repeat library to summarize enriched transposable elements (TEs) and complex repeats, (3) using de novo assembly methods to assemble contigs from the ChIP reads and calculating enrichment relative to input post hoc, and (4) mapping reads to a heterochromatin-enriched assembly [19] and calling ChIP peaks (Fig 1A).

In our ChIP experiments, CENP-A pulls down simple satellites, consistent with a previous study [16]. Among the kmers most enriched in CENP-A ChIP relative to input are the *dodeca* satellite and its variants and complex kmers that include tandem (AATAG)_n_ and (AATAT)_n_ repeats (Fig 1B, S1 Fig and S1 Table). *Prodsat* (Prod satellite; also known as the 10-bp satellite) is enriched in the CENP-A ChIP but not relative to input (Fig 1B). In addition to satellites, we found that CENP-A is also strongly associated with retroelements, particularly non-LTR long interspersed nuclear element (LINE)-like elements in the *Jockey* family and with the intergenic spacer of the ribosomal genes (IGS). Among the *Jockey* elements, the most highly enriched in CENP-A ChIPs are annotated as *G2* and *Jockey-3* (Fig 1C and S2 Table). Our phylogenetic analysis suggests that *G2* and *Jockey-3* correspond to the same type of element, as genomic copies of these two elements are interleaved across the tree and not monophyletic (S2 Fig). Thus, we hereafter collectively refer to these elements as *G2/Jockey-3*.

To detect CENP-A-enriched sequences independently of known repeats in repeat libraries or of genome assemblies, we de novo assembled CENP-A ChIP reads into contigs (i.e., ChIPtigs [20]) and calculated their CENP-A enrichments. The resulting CENP-A-enriched ChIPtigs primarily contained fragments of TEs, other complex repeats, and some simple satellite repeats (S3 Table).

**Fig 2:**
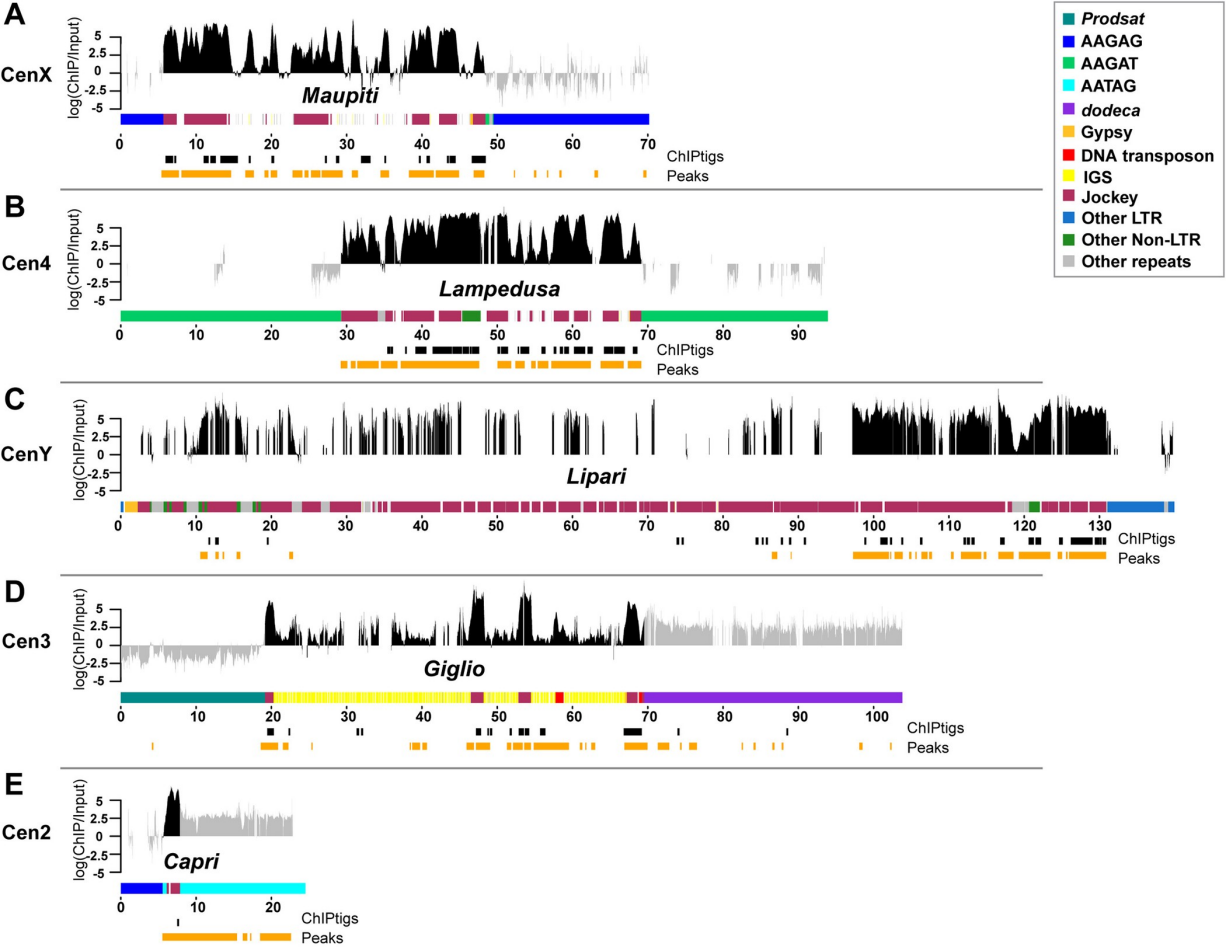
CENP-A occupies DNA sequences within putative centromere contigs. Organization of each CENP-A-enriched island corresponding to centromere candidates: (A) CenX, (B) Cen4; (C) CenY; (D) Cen3; (E) Cen2. Different repeat families are color coded (see legend; note that *Jockey* elements are shown in one color even though they are distinct elements). Shown are the normalized CENP-A enrichment over input (plotted on a log scale) from one replicate (replicate 2, other replicates are in S4 Fig) colored in gray for simple repeats and black for complex island sequences. Although the mapping quality scores are high in simple repeat regions, we do not use these data to make inferences about CENP-A distribution (see text for details). The coordinates of the significantly CENP-A-enriched ChIPtigs mapped to these contigs (black), and the predicted ChIP peaks (orange) are shown below each plot. See also S4 Fig and S3 and S4 Tables. Cen2, centromere 2; Cen3, centromere 3; Cen4, centromere 4; CENP-A, centromere protein A; CenX, X centromere; CenY, Y centromere; ChIP, chromatin immunoprecipitation; IGS, intergenic spacer of the ribosomal genes; LTR, long terminal repeat; *Prodsat*, Prod satellite.

To determine the genomic location of CENP-A-enriched sequences, we mapped ChIP reads to a new reference genome assembly that we generated using a heterochromatin-enriched assembly method resulting in greater representation of heterochromatin-associated regions [19] (S4 Table and S1 Text). Five contigs were consistently the most CENP-A enriched in the assembly, with highly reproducible ChIP peaks across technical and biological replicates (irreproducible discovery rate [IDR] < 0.05; S3 Fig and S5 Table). These CENP-A-enriched contigs have a similar organization: they contain islands of complex DNA (e.g., TEs) flanked by simple tandem satellite repeats with known centromeric locations (Fig 2, S4 Fig and Table 1). The candidate centromeric contig for the X chromosome (Contig79) is 70 kb and contains a 44-kb island of complex DNA (called *Maupiti* [15]), flanked by a short stretch of AAGAT satellite on one side and embedded in AAGAG satellite (Fig 2A). This region has an organization that is nearly identical to that of the *Dp1187* minichromosome putative centromere [14, 15], suggesting that this contig may contain at least part of the endogenous X centromere (CenX). The candidate centromeric contig for chromosome 4 (Contig119) contains a 42.8-kb island (we named *Lampedusa*) flanked by the AAGAT satellite (Fig 2B). This contig is consistent with the cytological location of the AAGAT satellite on chromosome 4 and with a recent report on the centromere of a B chromosome derived from chromosome 4 [21]. The candidate centromeric contig for chromosome Y (Y_Contig26) consists of a 138-kb island (we named *Lipari*; Fig 2C). The candidate centromeric contig for chromosome 3 (Contig 3R_5) contains a 68.5-kb island (we named *Giglio*) flanked by *Prodsat* and the *dodeca* satellite, which map to this centromere cytologically [12, 22, 23] (Fig 2D). Finally, the candidate contig for chromosome 2 (tig00057289) contains a small 1.8-kb complex island (we named *Capri*) flanked by the AATAG and AAGAG satellites (Fig 2E). The majority of the top enriched de novo ChIPtigs (88/100 for R1, 19/30 for R2, 26/30 for R3, and 82/100 for R4) map uniquely to these five contigs (S3 Table), providing independent support for the assembly and further substantiating our hypothesis that these contigs correspond to the centromeres.

**Table 1:** Location of centromeric and centromere-proximal satellites in *D*. *melanogaster*. Locations of satellites on chromosomes X, Y, 2, 3, and 4 according to previous reports and our observations in this report by IF-FISH in the *D*. *melanogaster* sequenced strain iso-1. Each satellite location is characterized as being centromeric (overlaps with CENP-C), pericentric (juxtaposed to CENP-C), or heterochromatic (more distal than pericentric). Note that the *dodeca* satellite includes its variants and that *Prodsat* is also known as the 10-bp satellite.

		Previous Reports			This Study		
Satellite	Sequence	Cen	Peri	Het	Cen	Peri	Het
AATAT^a^^,^^b^^,^^c^^,^^d^	(AATAT)_n_	X	-	3,4,Y	X	Y	3,4,Y
AAGAG^a^^,^^b^^,^^c^	(AAGAG)_n_	X	-	2,3,4,Y	2,X	4	3,Y
AATAG^a^^,^^b^	(AATAG)_n_	-	-	2,Y	2*	3	2,Y
AAGAT^e^	(AAGAT)_n_	4	-	-	4	X	2
*dodeca*^f^	(CGGTCCCGTACT/GGTCCCGTACT)_n_	3	-	-	3	-	-
*Prodsat*^a^^,^^g^	(AATAACATAG)_n_	-	2,3	-	2	2,3	-

*Indicates a small block not easily detected by FISH. See also S7 Fig.

^**a**^Lohe et al., 1993 [24]; Jagannathan et al. 2017 [25].

^**b**^Talbert et al., 2018 [16].

^**c**^Sun et al., 2003 [14].

^**d**^Tolchov et al., 2000 [26].

^**e**^Hanlon et al., 2018 [21].

^**f**^Abad et al., 1992 [27]; Garavís et al., 2015 [12]; Jagannathan et al., 2017 [25].

^**g**^Torok et al., 1997,2000 [28, 29]; Blower and Karpen, 2001 [30]; Garavis et al., 2015 [12].

Abbreviations: Cen, centromeric; CENP-C, centromere protein C; FISH, fluorescence in situ hybridization; Het, heterochromatic; IF, immunofluorescence; Peri, pericentric; *Prodsat*, Prod satellite.

### Genomic distribution of CENP-A in embryos and S2 cells

Our ChIP-seq experiments and their analyses provide evidence that CENP-A is specifically associated with the island DNA sequences for Contig79 (X^*Maupiti*^), Contig119 (4^*Lampedusa*^), Y_Contig26 (Y^*Lipari*^), and 3R_5 (3^*Giglio*^) and with a single interspersed *G2/Jockey-3* fragment within tig00057289 (2^*Capri*^; Fig 2 and S4 Fig). A previous study that used a *D*. *melanogaster* native ChIP-seq dataset (using anti–green fluorescent protein [GFP] antibodies and CENP-A–GFP-expressing embryos) focused exclusively on the quantification of simple repeats and did not identify any complex DNA associated with CENP-A [16]. However, our reanalysis of this dataset showed association of CENP-A–GFP with the centromere islands (S3B Fig and S4 and S6 Tables). We validated individual elements for which we could design contig-specific quantitative PCR (qPCR) primers in additional independent CENP-A ChIP experiments and confirmed that the CENP-A peaks in these regions are not a result of library amplification bias from ChIP-seq (S5 Fig) [31].

Having shown that CENP-A is associated with the complex islands, we next analyzed if the centromere extends to the surrounding satellite DNA. Simple sequences flanking the islands appear among the kmers enriched in the CENP-A ChIP (Fig 1B, S1 Table and S1 Fig). However, it is difficult to quantify the enrichment of CENP-A on simple satellite repeats for several reasons: (1) simple satellite sequences may be over- or underrepresented as an artifact of library preparation [31], particularly for ChIP-seq experiments that rely on PCR amplification to construct libraries; (2) satellites are abundant genomic sequences that are largely missing from whole genome assemblies [10], making it difficult to precisely quantitate how much of these sequences exist in genomes (and therefore how much to expect in the input); (3) highly abundant repeats are expected to have a low signal-to-noise ratio if only a small fraction of a simple repeat is enriched in CENP-A relative to the overall abundance of this satellite in the genome; and (4) simple satellite repeats present a challenge for even long read–based genome assembly methods [32]. Whereas we are confident in large-scale structural features of our assembly involving highly repetitive sequences, we observe even PacBio read depth in islands but not on simple satellites (S6 Fig), giving us less confidence in the base pair resolution of the assembly at simple repeats. Because of these limitations, we caution against using strictly assembly-based approaches in regions with simple repeats. Nonetheless, we report the ChIP peaks on simple satellites (shaded in gray in Fig 2). To confirm satellite localization near each centromere, we employed immunofluorescence (IF) with anti-centromere protein C (CENP-C; an inner kinetochore protein that colocalizes with CENP-A), followed by fluorescence in situ hybridization (FISH) with probes for the satellites *dodeca*, AAGAG, AATAT, AAGAT, AATAG, and *Prodsat* on metaphase chromosome spreads from third instar larval brains (S7 Fig); a summary of the colocalization data is shown in Table 1.

Although CENP-A localizes exclusively to the centromeres at the cytological level, it is possible that low levels of CENP-A occupy noncentromeric DNA. We found a low but consistent CENP-A enrichment at genomic regions outside of the centromere islands, including some telomere-associated elements (e.g., *TART-A*), rDNA genes from the rDNA clusters, and the LINE-like retroelements DMRT1B and R2 (Fig 1C, S4 Table and S1 Text). Many of these associations likely represent nonspecific peaks [33], as they were not highly enriched in CENP-A ChIP-qPCR (S5 Fig). However, previous studies found evidence for an association of some centromeric proteins with the nucleolus [34], perhaps relating to the possible association between CENP-A and rDNA or rDNA-associated retroelements (e.g., R2) that we detect. We also noted that noncentromeric copies of *G2/Jockey-3* were not consistently enriched in CENP-A (S8 Table).

CENP-A ChIP-seq reads from S2 cells showed a similar enrichment profile of sequences represented in the embryo ChIP-seq data (e.g., IGS and *G2/Jockey-3*) but were much more enriched for additional retroelements that were not represented within our centromere contigs (e.g., LTR elements *Dm1731*, *HMSBeagle*, and *Max-I*; S2 Table). We also observed a similar pattern of CENP-A enrichment on simple satellite repeats in S2 cells (AATAT, AATAG, AAGAG, *Prodsat*, and *dodeca*; S1 Table), and we confirmed that these satellites are near centromeres cytologically using IF-FISH in S2 cells (S8 Fig). However, complex satellites that are pericentric in embryos, including complex satellites in the 1.688 family and *Responder* (*Rsp*), are CENP-A-enriched in S2 cells (S2 Table). This suggests that the centromeres of S2 cells may have expanded into regions that are pericentromeric in flies; the additional retroelements enriched in CENP-A may be pericentric or they may represent new retroelement insertions occurred in this cell line. Our findings are consistent with the extensive structural rearrangements and aneuploidy reported for these cells [35].

### Centromeres are unique but are composed of similar non-LTR retrotransposons

**Fig 3:**
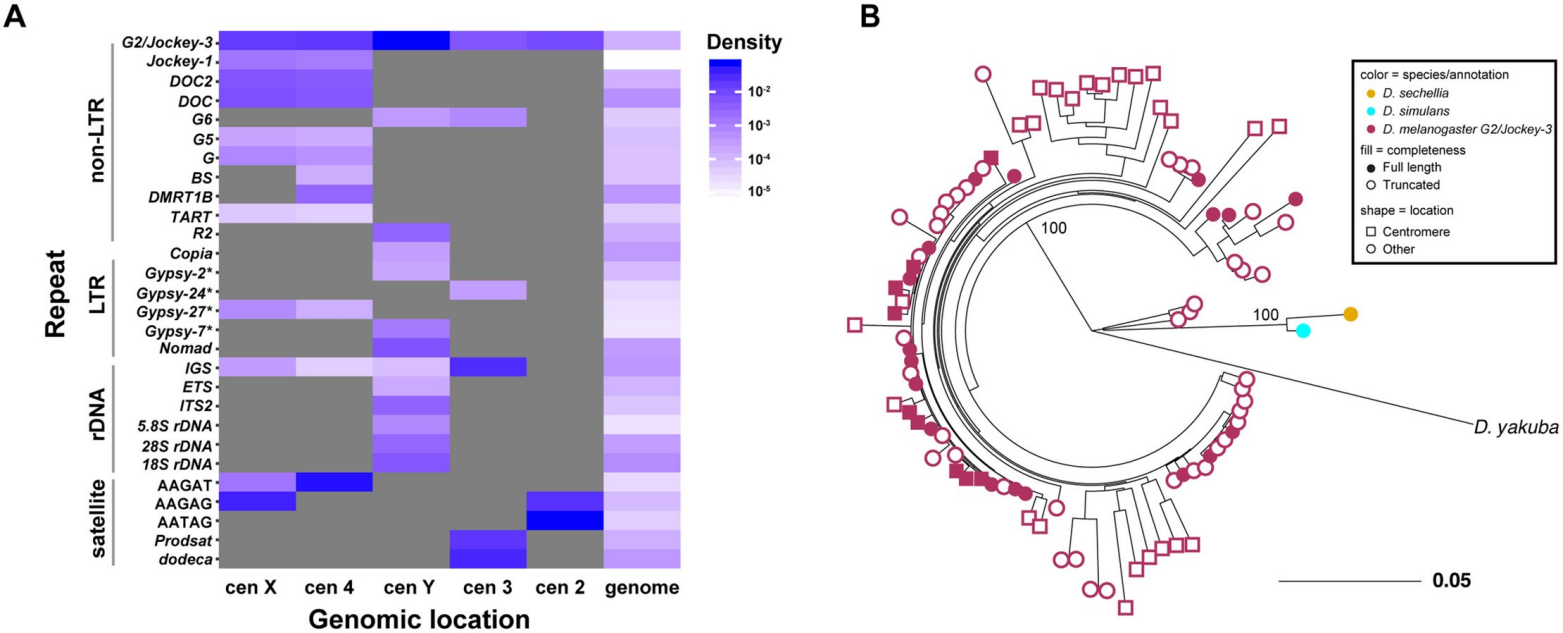
Centromeres are enriched in non-LTR retroelements in the *Jockey* family. (A) Density of all repetitive elements on each candidate centromere contig and the entire genome (minus the centromeres) grouped by type: non-LTR retroelements, LTR retroelements, rDNA-related sequences, and simple satellites. *G2/Jockey-3* is present on all centromeres. An * indicates annotations based on similarity to retroelements in other *Drosophila* species: *Gypsy-2* is from *D*. *simulans*, *Gypsy-24* and *Gypsy-27* are from *D*. *yakuba*, and *Gypsy-7* is from *D*. *sechellia*. For annotation, see Dryad repository file 9: https://doi.org/10.5061/dryad.rb1bt3j [37]. The underlying data can be found in S1 Data. (B) Maximum-likelihood phylogenetic tree based on the entire sequence of all *G2/Jockey-3* copies in *D*. *melanogaster* inside (squares) and outside (circles) of centromeric contigs and on the consensus repeat in its sister species *D*. *sechellia* and *D*. *simulans* and a more distantly related species (*D*. *yakuba*). The tree shows that centromeric *G2/Jockey-3* elements do not have a single origin (see Dryad repository files 13 and 15: https://doi.org/10.5061/dryad.rb1bt3j [37]). Cen2, centromere 2; Cen3, centromere 3; Cen4, centromere 4; CenX, X centromere; CenY, Y centromere; *ETS*, external transcribed spacer; IGS, intergenic spacer of the ribosomal genes; *ITS*, internal transcribed spacer; LTR, long terminal repeat; *Prodsat*, Prod satellite; *TART*, Telomere-associated retrotransposon.

**Fig 4:**
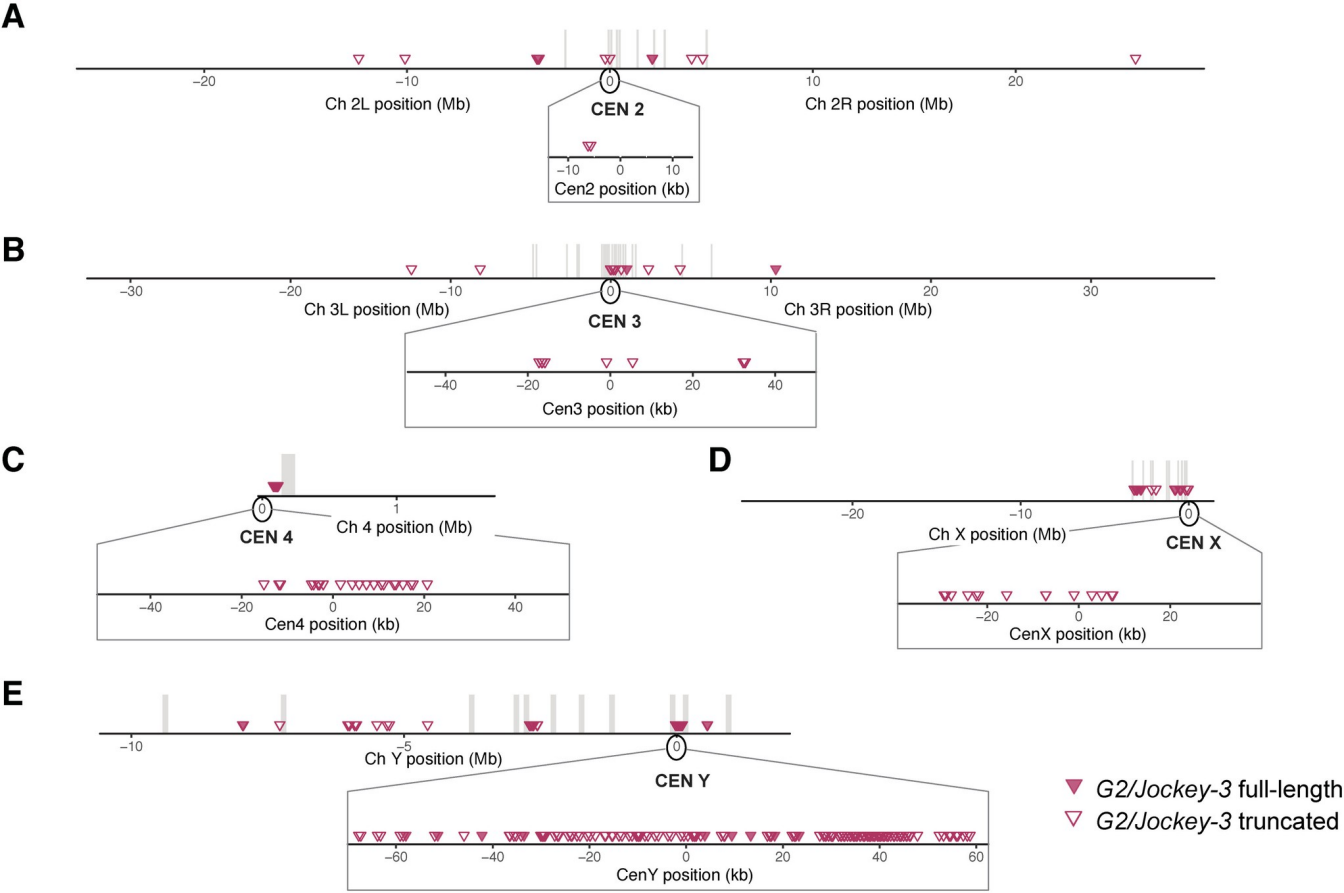
Genomic distribution of *G2/Jockey-3* elements in the *D*. *melanogaster* genome. Location of *G2/Jockey-3* elements across chromosome (“Ch”) 2 (A), 3 (B), 4 (C), X (D), and Y (E). Contigs from each chromosome were concatenated in order with an arbitrary insertion of 100 kb of “N.” Distances along the x-axis are approximate. The order and orientation of the Y chromosome contigs is based on gene order (see [19]). Each triangle corresponds to one TE, for which filled shapes indicate full-length TEs and open shapes indicate truncated TEs. The vertical gray bars represent the arbitrary 100-kb window inserted between contigs, indicating where there are gaps in our assembly. The centromere (“CEN”) positions are set to 0 for each chromosome. The insets zoom in to show the distribution of *G2/Jockey-3* elements on the centromere contigs. Chromosomes are not drawn to scale (chromosome 4 and Y are enlarged). TE, transposable element.

Although each island has a distinct arrangement of AT-rich sequences, repeats, and TEs, their composition is overall similar. In particular, non-LTR retroelements in the *Jockey* family such as *G2*/*Jockey-3*, *Doc*, and *Doc-2* are especially abundant within CenX, Cen4, and CenY (Figs 2 and 3A). *G2/Jockey-3* is the only element present in all five of our centromere contigs, suggesting a potential role in centromere function or specification. In our phylogenetic analysis of genomic *G2/Jockey-3* repeats in *D*. *melanogaster*, we cannot distinguish *G2/Jockey*-3 elements at centromeres from those across the genome, suggesting that centromeric copies do not have a single origin (Fig 3B and S1 Text). Although *G2/Jockey-*3 is not unique to centromeres and thus cannot be sufficient for centromere identity, it is significantly enriched at centromeres: approximately 63% of all genomic copies of *G2/Jockey-3* are found within our candidate centromere contigs (Fig 4 and S9 Table). *G2/Jockey-3* elements show signs of recent or ongoing activity based on their insertion polymorphism [36], pattern of 5′ truncation (see S1 Text and Dryad repository file 13: https://doi.org/10.5061/dryad.rb1bt3j [37]), and expression (S9A Fig). At least some of this expression comes from the centromeres: we analyzed total embryo RNA extracts by reverse-transcription qPCR (RT-qPCR) using primers targeting centromere-associated copies and found evidence for low levels of *G2/Jockey-3* transcription from copies in CenX, Cen4, and Cen3. We found no or negligible expression from the *G2/Jockey-3* copies that we measured on CenY and centromere Cen2 (S9B Fig).

In addition to *G2/Jockey-3*, the 3^*Giglio*^ island has 240 copies of a centromere-enriched variant of the ribosomal IGS (S1 Text and S10 Fig). Among the islands, 2^*Capri*^ differs the most, being the smallest and harboring only a single fragment of *G2/Jockey-3* (Fig 2E). As was previously reported for the X-derived *Dp1187* centromere [14, 15], none of the sequences contained within these islands are exclusive to centromeres. However, several of these elements are enriched in these regions compared to the genome in addition to *G2/Jockey-3*. For example, *Doc2*, *G*, and *Jockey-1* elements are non-LTR retroelements enriched in CENP-A with a genomic distribution biased toward centromeres (Fig 3A, columns labeled “genome”; S11 Fig and S9 Table).

### Validation of centromeric contigs

**Fig 5:**
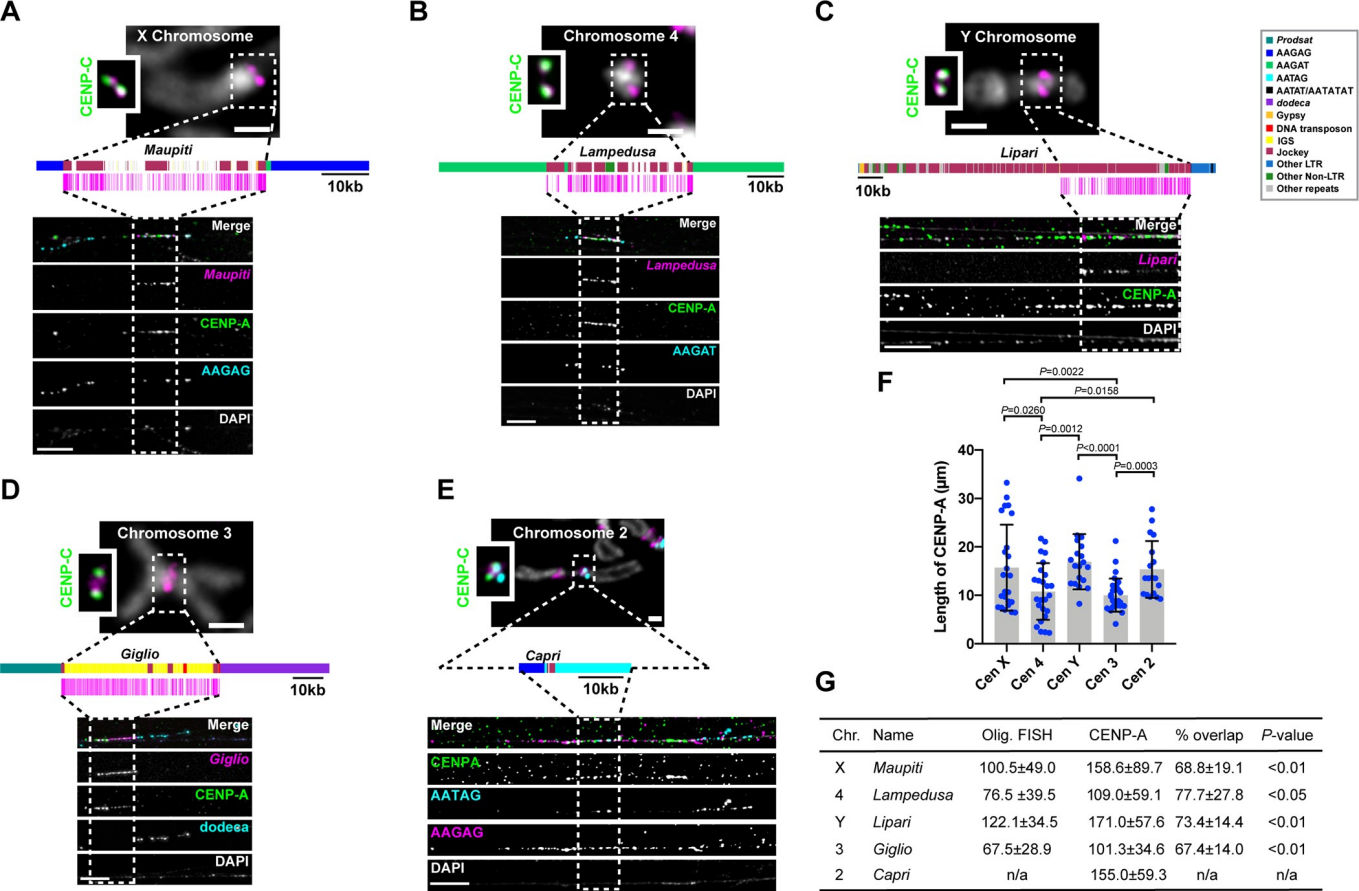
Islands of complex DNA are major components of centromeres. (A-D) Top, mitotic chromosomes from male larval brains showing IF with anti-CENP-C antibodies (green, inset) and FISH with chromosome-specific Oligopaints (magenta). Bar 1 μm. Middle, schematic of centromere contigs (see key) and location of Oligopaint probes (magenta). Bottom, IF-FISH on extended chromatin fibers from female larval brains. Anti-CENP-A antibodies (green), Oligopaints FISH (in panels A, B, and D; magenta), and centromere-specific satellites (cyan, and in E also in magenta). Dashed rectangles show the span of the Oligopaint probes, except for (E), where it is placed arbitrarily within the CENP-A domain where the Cen2 contig could be located. Bar 5 μm. (A) CenX; (B) Cen4; (C) CenY; (D) Cen3 (see also S20 Fig); (E) Cen2 using FISH probes AAGAG (magenta) and AATAG (cyan). The scale shown for the Cen2 diagram is approximate. (F) Scatterplot of CENP-A IF signal length for each centromere. Error bars = SD. *n* = 18–30 fibers for each centromere. Significant *P* values are shown (unpaired *t* test). The underlying data can be found in S1 Data. (G) Table showing the lengths of Oligopaint (“Olig.”) FISH and CENP-A IF signals on fibers (kb ± SD estimated based on 10 μm = 101 kb; S15 Fig). Percent overlap corresponds to CENP-A domain length/Oligopaint FISH length. The difference between the sizes of the CENP-A domain and the corresponding islands is significant (unpaired *t* test). Additional fibers are shown in S16, S17, S18, S19, S20 and S21 Figs. Cen2, centromere 2; Cen3, centromere 3; Cen4, centromere 4; CENP-A, centromere protein A; CENP-C, centromere protein C; CenX, X centromere; CenY, Y centromere; FISH, fluorescence in situ hybridization; IF, immunofluorescence; IGS, intergenic spacer of the ribosomal genes; n/a, not applicable; *Prodsat*, Prod satellite.

To verify the association of our contigs with the centromeres, we performed IF with anti-CENP-C antibodies, followed by FISH with satellite probes and custom-designed Oligopaints libraries [38] (see Materials and methods) for X^*Maupiti*^, 4^*Lampedusa*^, Y^*Lipari*^, and 3^*Giglio*^ (Fig 5, S12 Fig, and S1 Text). The X^*Maupiti*^ Oligopaints hybridized to CenX as well as CenY on third instar male larval brain metaphase spreads (Fig 5A and S12A Fig). Similarly, the Oligopaints for 4^*Lampedusa*^ hybridized to Cen4 as well as to CenY (Fig 5B and S12B Fig), suggesting that Oligopaints for X^*Maupiti*^ and 4^*Lampedusa*^ have homology to sequences at or near CenY. In contrast, the Oligopaints for Y^*Lipari*^ (Fig 5C and S12C Fig) and 3^*Giglio*^ were specific for their respective centromeres (Fig 5D and S12D Fig). We could not use Oligopaints to validate 2^*Capri*^ because of its small size, but its organization, with the AATAG and AAGAG satellites flanking a small CENP-A-enriched island (Fig 2E), is consistent with our FISH analyses (Fig 5E). In line with the CENP-A ChIP-seq data, we observed significant differences between S2 cells and embryo centromeres by Oligopaint FISH. With the exception of 3^*Giglio*^, centromeric island organization in S2 cells is dramatically different from larval brain metaphase spreads (S13 Fig and S1 Text), in contrast to the conservation of the centromeric distribution of simple satellites (S8 Fig).

*D*. *melanogaster* centromeres tend to cluster in the interphase nucleus cytologically [39, 40]. We found independent support for the complex islands being centromeric by analyzing previously published Hi-C data from *D*. *melanogaster* embryos [41]. Island–island interactions were among the most frequent interchromosomal interactions, followed by interactions between islands and their own proximal pericentric heterochromatin and lastly by interactions between islands and distal pericentric heterochromatin or euchromatin (S14 Fig and S1 Text). This analysis also shows that indeed native centromeres interact with one another physically in the 3D nucleus.

### Analysis of extended chromatin fibers reveals that CENP-A primarily occupies the islands

Based on the enrichment of CENP-A with island-associated repeats, we hypothesized that the TE-enriched islands are major centromere components in *D*. *melanogaster*. To test this, we investigated CENP-A occupancy, a direct reflection of centromere activity, and estimated the size of each centromere by visualizing extended chromatin fibers [11, 42]. This method has two major advantages: it does not rely on mapping low complexity ChIP-seq reads, thus providing more information that can be inferred by this method, and it affords single-chromosome, rather than population, information on CENP-A localization. We carried out IF with anti-CENP-A antibodies and FISH with Oligopaint and satellite probes on cells from third instar larval brains, selecting females to ensure specificity for our X^*Maupiti*^ and 4^*Lampedusa*^ Oligopaints (Fig 5). First, we calibrated our fiber stretching using three FISH probes spanning 100 kb: two heterochromatic (one for the *Rsp* locus [43] and one Oligopaint targeting the pericentromere of chromosome 3L; see Materials and methods for coordinates) and one euchromatic (an Oligopaint targeting a region approximately 600 kb from the telomere of chromosome 3L; see Materials and methods). The estimated stretching for these fibers is approximately 10.1 kb/μm for all three locations, with no significant difference among them (*P* = 0.085; S15 Fig). We next determined the sizes of the CENP-A domain and corresponding island of each centromere (Fig 5 and S16, S17, S18, S19, S20 and S21 Figs). The size of the CENP-A domain varies between centromeres, ranging in mean size between 101 and 171 kb (about 11–17 μm), smaller than previous estimates that relied on the measuring of a mixture of centromeres in *Drosophila* Kc and S2 cells [11]. This is consistent with our ChIP-seq analysis suggesting that S2 cells may have expanded centromeres. X, Y, and 2 are the largest centromeres, whereas 3 and 4 are the smallest (Fig 5F and 5G). CENP-A primarily occupies the centromeric islands X^*Maupiti*^, 4^*Lampedusa*^, Y^*Lipari*^, and 3^*Giglio*^ (about 70% of the CENP-A domain overlaps with the Oligopaint FISH signal; Fig 5G and S16, S17, S18, S19, S20 and S21 Figs). In some fibers, the X^*Maupiti*^ Oligopaint FISH signal showed interspersion with FISH signal for the AAGAG satellite (S16 Fig); this could be due to nonspecific binding of the AAGAG probe during FISH, which is optimized for Oligopaint specificity, or to a possible collapse of AAGAG repeats in our assembly, including within *Maupiti*. We also noticed that the estimated length of the Oligopaint-stained region was larger than the size of *Maupiti* in our CenX contig (100.5 ± 49 kb versus 44 kb; Figs 2A and 5G), a discrepancy that we attribute to variability in *Maupiti* Oligopaint probe hybridization. Alternatively, there could be additional sequences with similarity to *Maupiti* interspersed in the flanking satellites nearby the contig (and not included in our assembly).

Analysis of Cen4 shows that the CENP-A domain overlaps primarily with 4^*Lampedusa*^ and partially with the flanking AAGAT satellite (Fig 5B and 5F and S17 Fig). The Oligopaints for Y^*Lipari*^ target only the part of the island with the highest enrichment of CENP-A (Fig 5C). Fibers for this centromere show a continuous CENP-A domain that extends past the FISH signal, likely representing the remainder of the Y^*Lipari*^ island (Fig 5C and S18 Fig).

Fibers for 3^*Giglio*^ show colocalization between CENP-A and the island as well as a short, variable region of colocalization with flanking *dodeca* satellite (Fig 5D, S19 Fig and S20 Fig). We did not observe CENP-A signal on the opposite side of *Giglio*, where *Prodsat* is located according to our assembly (Fig 5D). The Cen3 satellite *dodeca* colocalizes with CENP-A on fibers in S2 cells [12] and is highly enriched in our CENP-A ChIP-seq (Fig 1B and S1 Fig). When we tracked longer fibers from 3^*Giglio*^ along *dodeca*, we observed a second CENP-A domain in which *dodeca* is interrupted by short fragments of Oligopaint FISH signal (S20 Fig), suggesting the existence of DNA sequences with homology to *Giglio* interspersed within *dodeca* that are not included in our assembly. A previous study identified sequences with homology to IGS within the *dodeca* satellite in one bacterial artificial chromosome (BAC) [12]. It is possible that the *dodeca*-associated Oligopaint FISH signal in our extended fibers corresponds to these additional IGS sequences. These data indicate that Cen3 has two CENP-A domains, a major one on 3^*Giglio*^ and one minor one on *dodeca*, although these appear as a single domain in standard metaphase spread IF. Unlike Cen3, all other centromeres display a single CENP-A domain by fiber analysis (e.g., see S21 Fig for Cen2). Our conclusions differ from those of the Talbert et al. study [16], which concluded that *dodeca* was not associated with CENP-A. As recognized by the authors, it is possible that different chromatin preparations, such as the MNase digestion, may introduce biases, leading to an underrepresentation of sequences like *dodeca* in ChIPs [16].

Lastly, we analyzed the organization of 2^*Capri*^ using FISH with a satellite combination unique to this chromosome AATAG, AAGAG, and *Prodsat* and found that the CENP-A domain overlapped with all three satellites (Fig 5E and S21 Fig). Thus, we speculate that the *Prodsat* sequences pulled down by CENP-A as seen in our kmer analysis (Fig 1B) and reported previously [16] are coming from Cen2, not Cen3. We therefore conclude that *D*. *melanogaster* CENP-A is primarily associated with the centromeric islands of chromosomes X, 4, Y, and 3 and less predominantly with the flanking satellites (Fig 5G).

### *G2/Jockey-3* is centromere-associated in *D*. *simulans*

**Fig 6:**
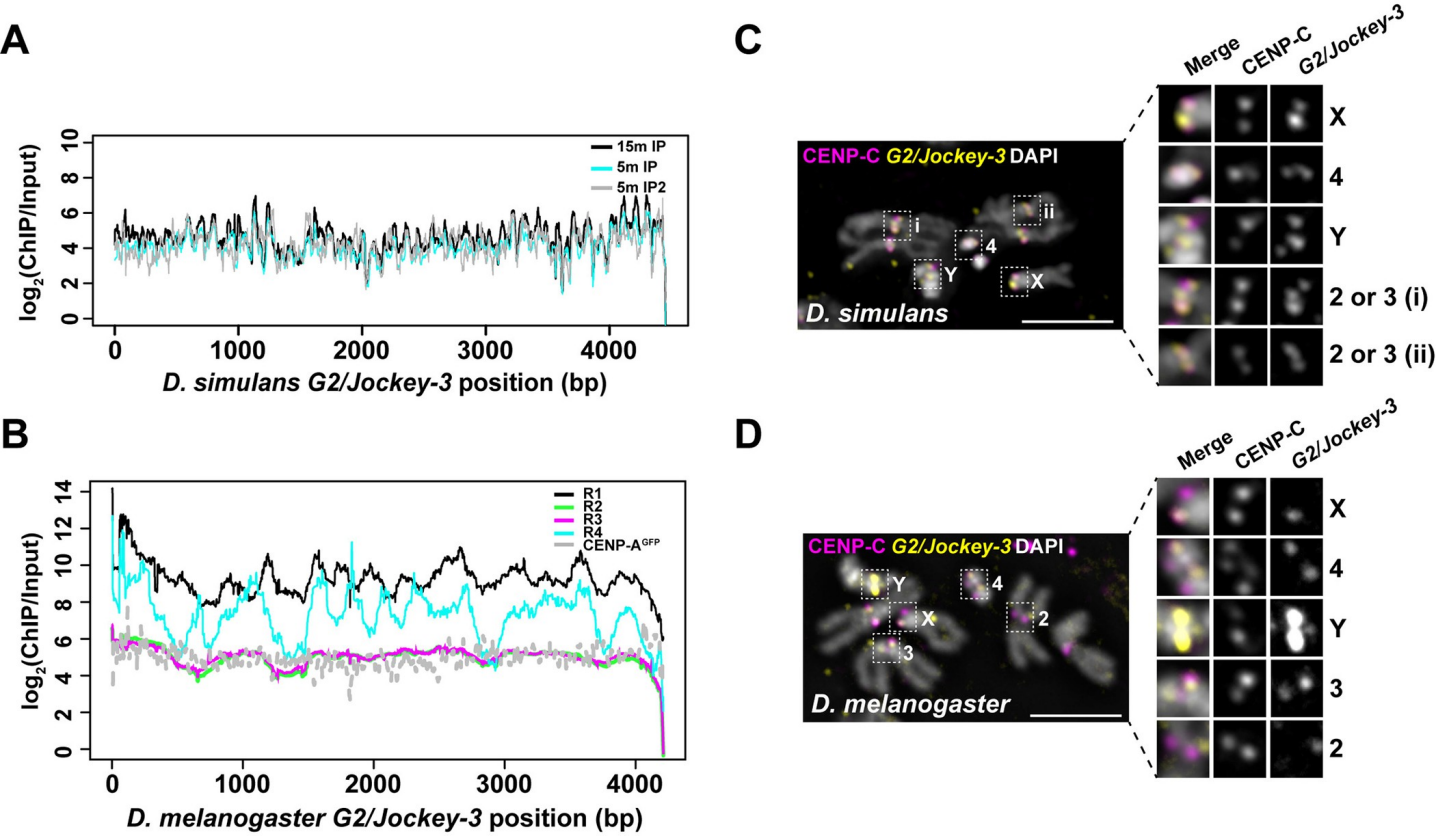
The association between *G2/Jockey-3* and centromeres is conserved in *D*. *simulans*. (A) Plot of the normalized CENP-A enrichment over input across the *D*. *simulans G2/Jockey-3* consensus sequence using CENP-A ChIP-seq data from *D*. *simulans* ML82-19a cells [16] showing that *G2/Jockey-3* is enriched in CENP-A in *D*. *simulans*. The labels “15m” and “5m” indicate minutes of MNase digestion, and IP and IP2 are technical replicates. Note that the first 487 bp of *D*. *simulans G2/Jockey-3* consensus sequence, which are homologous to the *D*. *simulans* 500-bp satellite, are not included in this figure; the 500-bp satellite was previously reported as enriched in CENP-A in *D*. *simulans* [16]. (B) Plot of the normalized CENP-A enrichment over input across the *D*. *melanogaster G2/Jockey-3* consensus sequence using our CENP-A ChIP-seq replicates (R1–R4) and ChIP-seq from CENP-A–GFP transgenic flies from Talbert and colleagues [16]. The underlying data for (A-B) can be found in S1 Data. IF-FISH on (C) *D*. *simulans* (w501) and (D) *D*. *melanogaster* (iso-1) mitotic chromosomes from male larval brains using an antibody for CENP-C (magenta) and FISH with a *G2/Jockey-3* DIG-labeled FISH probe (yellow). DAPI is shown in gray. Bar 5 μm. CENP-A, centromere protein A; CENP-C, centromere protein C; ChIP, chromatin immunoprecipitation; ChIP-seq, ChIP sequencing; DIG, digoxigenin; FISH, fluorescence in situ hybridization; GFP, green fluorescent protein; IF, immunofluorescence; IP, immunoprecipitation.

The *G2/Jockey-3* retroelement is a recently active transposon [36] shared among all *D*. *melanogaster* centromeres (Fig 3A). To determine if *G2/Jockey-3* is enriched at the centromeres outside of *D*. *melanogaster*, we investigated its centromeric distribution in its sister species, *D*. *simulans*, which diverged from *D*. *melanogaster* only about 2 million years ago [44] and yet displays major differences in satellite composition and distribution [25, 45]. These differences are especially apparent in centromeric regions, where *D*. *melanogaster* displays simple satellite repeats whereas *D*. *simulans* contains complex satellite repeats with larger repeat units [16]. We reanalyzed published *D*. *simulans* cell line CENP-A ChIP-seq data [16] (see S1 Text) and found that *G2/Jockey-3* elements are also highly enriched in CENP-A in this species, as in *D*. *melanogaster*. The pileups of CENP-A ChIP-seq reads on *G2/Jockey-3* show that CENP-A is associated with the entire length of the retroelement in both *D*. *simulans* and *D*. *melanogaster*, with no apparent affinity for any particular sequence (Fig 6A and 6B).

To validate the association of *G2/Jockey-3* with *D*. *simulans* centromeres, we designed a FISH probe that targets about 1.6 kb at the 3′ of the *D*. *melanogaster G2/Jockey-3* consensus sequence (see Materials and methods; approximately 94% identical to *D*. *simulans G2/Jockey-*3 consensus sequence) and performed IF-FISH on male larval brain metaphase spreads with anti-CENP-C antibodies, which recognize CENP-C in both species [46]. We observed colocalization between CENP-C and *G2/Jockey-3* at all *D*. *simulans* centromeres (Fig 6C; note that chromosome 2 and 3 of *D*. *simulans* cannot be distinguished morphologically [25]). The same probe showed colocalization of CENP-C and *G2/Jockey-3* at all *D*. *melanogaster* centromeres, except at Cen2, which is consistent with our model for this centromere showing only one copy of *G2/Jockey-3* (Figs 6D and 2E). Based on these observations, we infer that *G2/Jockey-3* is a conserved centromere-associated retroelement in these species.

## Discussion

**Fig 7:**
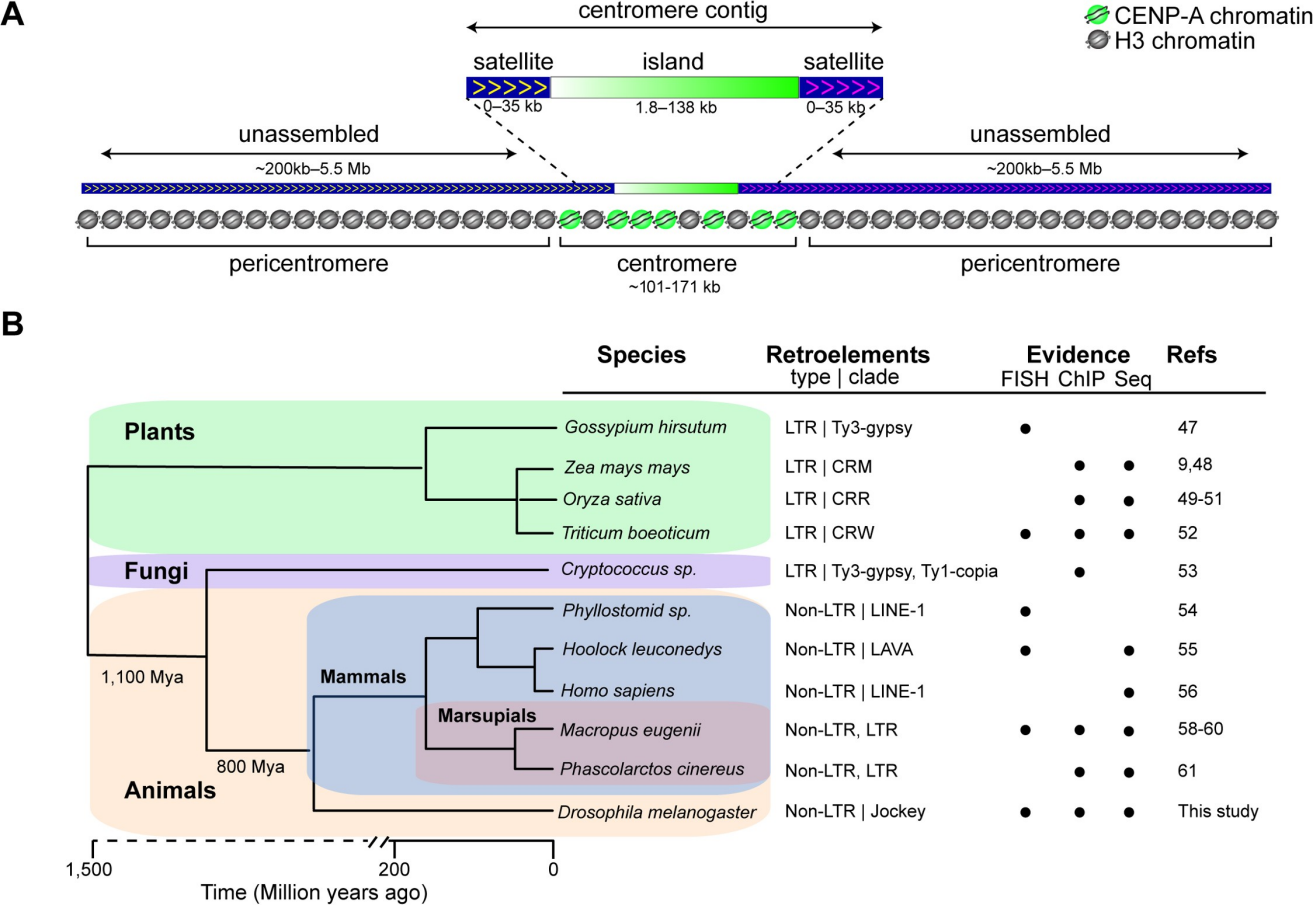
*Drosophila* centromere organization and widespread presence of retroelements at centromeres. (A) Schematic showing the organization of *D*. *melanogaster* centromeres. For at least CenX, Cen4, and Cen3, the bulk of CENP-A chromatin is associated with the centromere islands, whereas the remaining CENP-A is on the flanking satellites. The sequences flanking the Y centromere are not in our assembly, so whether CENP-A is also on satellites is unknown. Although the complexity of island DNA allowed us to identify centromere contigs by long-read sequencing, the flanking satellites remain largely missing from our genome assembly because of their highly repetitive nature. The approximate satellite size estimates are based on Jagannathan and colleagues’ work [25]. (B) Phylogenetic tree showing that centromere-associated retroelements are common across highly diverged lineages: *Gossypium hirsutum* (cotton) [47], *Zea mays mays* (maize) [9, 48], *Oryza sativa* (rice) [49–51], *Triticum boeoticum* (wild wheat) [52], *Cryptococcus* [53], *Phyllostomid* (bat) [54], *Hoolock leuconedys* (gibbon) [55], *Homo sapiens* (human) [56] (and a human neocentromere [57]), *Macropus eugenii* (tammar wallaby) [58–60], *Phascolarctos cinereus* (koala) [61], and *D*. *melanogaster* (this study for endogenous centromeres; also in an X-derived minichromosome [14, 15]). The phylogeny was constructed using TimeTree [62]. Indicated are the retroelement type and the clade that the element belongs to with element types as follows: LTR and non-LTR. The circles indicate the experimental evidence for centromere association of retroelements: FISH, CENP-A ChIP-seq (ChIP), and genome or BAC sequencing (Seq). BAC, bacterial artificial chromosome; CENP-A, centromere protein A; CenX, X centromere; ChIP-seq, chromatin immunoprecipitation sequencing; CRM, centromeric retrotransposons of maize; CRR, centromeric retrotransposons of rice; CRW, centromeric retrotransposons of wheat; FISH, fluorescence in situ hybridization; LAVA, LINE-Alu-VNTR-Alu-like; LINE, long interspersed nuclear element; LTR, long terminal repeat; Mya, million years ago.

Our study shows that combining long-read sequencing with ChIP-seq and chromatin fiber FISH is a powerful approach to discover centromeric DNA sequences and their organization. We reveal that for all but one chromosome (chromosome 2, which has a single *G2/Jockey-3* element), approximately 70% of the functional centromeric DNA of *D*. *melanogaster* is composed of complex DNA islands. The islands are rich in non-LTR retroelements and are buried within large blocks of tandem repeats (Fig 7A). They likely went undetected in previous studies of centromere organization (e.g., [12]) because three of the five islands are either missing or incomplete in the published reference *D*. *melanogaster* genome [10]. A recent study reported that satellite DNA repeats make up the majority of centromeric DNA in *D*. *melanogaster* embryos and S2 cells, by counting the relative number of motifs matching simple repeats in CENP-A ChIP relative to input [16]. Our reanalysis of those data showed CENP-A enrichment on the islands, suggesting that having an improved reference genome assembly [19] is crucial for identifying centromeric DNA sequences. To our knowledge, this is the first detailed report on the linear sequence of all centromeres in a multicellular organism. Our overall strategy therefore provides a blueprint for determining the composition and organization of centromeric DNA in other species.

To date, satellite DNAs have been regarded as the main sequence components of the centromeres of primary animal model systems—humans, mice, and *Drosophila* [2, 3, 17]. However, retroelements are abundant and widespread at the centromeres of plants such as maize [48] and rice [49, 50]. Retroelements are also found at the centromeres of fungi [53], humans [56], marsupials [63], bats [54], and gibbons [55], suggesting that they may be common centromeric features (Fig 7B). Our study shows that retroelements, particularly *G2/Jockey-3*, are not merely present near centromeres but are components of the active centromere cores through their association with CENP-A. Our BLAST search for *G2/Jockey-3* retroelements suggests that they are restricted to the *melanogaster* subgroup; therefore, we hypothesize that different non-LTR retroelements may be present at the centromeres of other *Drosophila* species. Why retroelements are such ubiquitous components of centromeres and whether they play an active role in centromere function remain open questions. In maize, centromeric retroelements invade neocentromeres following their inception [64], suggesting a preference for DNA sequences associated with CENP-A chromatin for retroelement insertions [18]. On the other hand, evolutionarily new centromeres in *Equus asinus* lie in LINE-rich regions [65], and a LINE element was found to be an integral component of a human neocentromere [57, 66], raising the possibility that it is CENP-A that may bind preferentially to retroelement-associated genomic regions [18]. Other models have proposed that retroelements could produce noncoding RNAs that affect centromere specification [18, 66] and that retroelement activity could help maintain centromere size through retrotransposition or by giving rise to tandem repeats via recombination-mediated mechanisms (e.g., [67, 68]; reviewed in [69]).

Centromeric transcription contributes to centromere homeostasis in several organisms, including fission yeast [70, 71], wallaby [72], human [6, 73], and *Drosophila* cells [74, 75]. Our preliminary analysis with quantitative RT-PCR using centromere-specific *G2/Jockey-3* primer sets shows some evidence for low levels of centromere expression.

In addition to retroelements, the centromeres of *D*. *melanogaster* display a diverse assortment of repeats, none of which are exclusive to centromeres, with the exception of IGS, for which we identified a centromere-enriched variant. The identification of the IGS tandem repeat within *3^Giglio^* is intriguing, as IGS sequences are dynamic in the potato [76], where they are located near the centromere, as well as in the tobacco [77], the tomato [78], and the common bean [79], where they show a dispersed pattern over several chromosomes. The origin of novel tandem repeats is still elusive, but one way it has been proposed to occur for the IGS repeat in plants is through the initial insertion of a retroelement within rDNA, followed by IGS duplication, amplification, and transposition to a new locus [78].

Defining the span of the CENP-A domain is important to understand precisely which sequences are associated with centromere activity and which are part of pericentric heterochromatin. Although we are able to confidently map our ChIP-seq reads to the islands to determine CENP-A occupancy, the same cannot be done for simple satellites, because of the limitations of mapping to highly repetitive DNA. We therefore infer the organization of the centromere from analyzing extended chromatin fibers by IF-FISH. Blocks of simple satellite sequences flank the islands on each of our contigs, with the exception of the CenY contig. However, these regions represent only a fraction of the estimated abundance of those repeats in the genome. For example, the *dodeca* satellite occupies approximately 1 Mb of the genome [27], yet only about 570 kb of *dodeca* sequence are included in the assembly, with just roughly 35 kb of *dodeca* on the Cen3 contig. Therefore, for many satellite sequences, inferences based on read mapping, even uniquely mapped reads, are confounded by the underrepresentation of satellites in the assembly. Our analysis of chromatin fibers suggests that CENP-A spans beyond the islands into the simple satellites, although the precise boundaries remain unknown (Fig 7A).

The finding that CENP-A can bind to several different sequences that are not uniquely associated with centromere regions is consistent with the epigenetic model of centromere specification, which proposes that specific sequences alone do not govern centromere activity [3]. Yet it is possible that the diverse sequence arrangements observed at each centromere somehow contribute to centromere activity or specification [18, 48]. Possible mechanisms include the promotion of unusual types of transcription, as reported for fission yeast [80], or the formation of non-B DNA structures (e.g., stem loops, hairpins, and triplexes) that may promote CENP-A deposition [7, 12, 81]. Knowing the identity of *D*. *melanogaster* centromeric DNA will enable the functional interrogation of these elements in this powerhouse model organism.

## Materials and methods

### ChIP-seq

CENP-A ChIPs were performed using an affinity purified rabbit anti-CENP-A antibody (gift of Gary Karpen) that we previously verified works well for ChIP using S2 cells that contain LacI/lacO inducible ectopic centromeres and showing that CENP-A ChIP pulled down lacO plasmid DNA sequences [82].

#### ChIP in embryos

Embryo (wild type line Oregon-R) collection, fixation, and chromatin isolation were performed as described in [83]. We carried out four ChIP replicates as follows. From one embryo collection, we generated chromatin used in R1; from a second independent embryo collection, we generated chromatin used for replicates R2–4. We used formaldehyde-crosslinked overnight collections of Oregon-R embryos (about 1.5 g per collection). Chromatin was sheared to 200–500 bp using a Bioruptor sonicator (Diagenode), aliquoted, and flash frozen. The first biological replicate (R1) was performed following the protocol in [83] using 165 μg of chromatin (in 500 μl volume and 30 μl of protein A agarose beads) and 2 μl of anti CENP-A antibody. For R2, 3, and 4, we used the MAGnify kit, with 15 μl of dynabeads, approximately 60 μg of chromatin in 200 μl volume, and 3 μl of anti-CENP-A antibody. Libraries were made from eluted DNA using the TruSeq ChIP kit (Illumina) for R1 and R4, whereas the Accel-NGS 2S Plus DNA Library (Swift Biosciences) was used for R2 and R3. Note that R2–3 were performed in parallel and sequenced the same way and are thus technical replicates. The libraries were sequenced by paired-end on the NextSeq platform using Reagent v.2. Chromatin extracted from the second embryo collection was also used for ChIP-qPCR experiments.

For both chromatin preparations, the quality of the chromatin was confirmed by control ChIPs with 15 μg of chromatin in 200 μl volume and 2 μl of rabbit anti-H3K27Ac (ThermoFisher). The eluted DNA was analyzed by qPCR confirming enrichment of the *RpL32* promoter (F-TTGTTGTGTCCTTCCAGCTTCA and R-TTGTTGTGTCCTTCCAGCTTCA) and lack of enrichment of *RpL32* 5′ region (F-GGCACGGCGCCAAAATTAATCA and R-CCGATGCCACTGCCTCTTTGGT) [84, 85].

#### ChIP in S2 cells

Chromatin from 10^6^ fixed *Drosophila* S2 cells (approximately 90 μg) were used for each IP, and chromatin was sheared to 100–300 bp using a Covaris sonicator. ChIPs were performed using the MAGnify kit (ThermoFisher). The anti-CENP-A antibody (1 μl) was coupled to 10 μl of beads for 2 h followed by incubation with chromatin overnight at 4°C. DNA was eluted in 50 μl of elution buffer. Libraries were generated using the TruSeq kit (Illumina) and paired-end sequenced using the Reagent kit v.3. (Illumina) on the NextSeq platform.

#### ChIP-seq quality control analyses

We estimated read quality of each replicate ChIP-seq experiment using two metrics estimated in phantompeakqualtools [86]: the normalized strand coefficient (NSC) and the relative strand correlation (RSC) (S6 Table). These statistics report the cross correlation between Watson and Crick strands, as ChIP reads from a true positive are expected to be highly clustered and accumulate on either side of the binding site on both strands, with a shift between the peaks on the Watson and Crick strands that is determined by read length and fragment length distribution [87]. This shift should not occur in the input. NSC is the fragment-length cross-correlation peak divided by the background cross correlation and RSC is the fragment-length cross-correlation peak divided by the read-length peak [86].

### Analysis of repeat enrichment in ChIP-seq replicates

To determine the CENP-A enrichment in simple tandem repeats, we summarized repeat composition in the trimmed reads and identified overrepresented kmers using kseek (https://github.com/weikevinhc/k-seek; [31]). The CENP-A/input ratio is normalized by the number of mapped reads to the genome assembly to remove possible read contamination. We consider a class of repeats to be enriched for CENP-A if the minimum number of kmers in the input is ≥10 in each replicate and the median normalized CENP-A/input ratio is >1 across all four replicate ChIP experiments (S1 Fig). Simple tandem repeats may be overrepresented or underrepresented because of Illumina library preparation and the effects of PCR amplification on sequence library complexity. To determine CENP-A enrichment on complex repeats, we used a mapping approach. We annotated repeats in our assembly [19] using a custom *Drosophila*-specific consensus repeat library [43] modified from Repbase to include complex satellite DNAs (Repbase version 20150807; [88]; Dryad repository file 1: https://doi.org/10.5061/dryad.rb1bt3j [37]). Using these RepeatMasker annotations, we generated a comprehensive library of all individual repetitive elements in the genome to capture sequence variation among repeats. We mapped ChIP and input reads to this comprehensive repeat library using bowtie2 (default settings) and summarized read counts for each type of complex repeat (e.g., TEs, complex satellite DNAs with repeat units > 100 bp) using custom python scripts. The CENP-A/input ratio is normalized by the number of mapped reads to the genome assembly. We consider a class of repeats to be enriched for CENP-A if it is in top 20th percentile of normalized CENP-A/input in all four replicate ChIP experiments.

To address if any motif in *G2/Jockey-3* is particularly enriched for CENP-A, we constructed a consensus sequence of *G2/Jockey-3* in *D*. *melanogaster* and *D*. *simulans*. We mapped ChIP and input reads to this comprehensive repeat library with only one version of *G2/Jockey-3* (either *D*. *melanogaster* or *D*. *simulans*) using bwa (default settings). We then called the depth of reads with samtools depth (v1.7) using “-Q 10 (mapping quality ≥ 10)” and calculated ChIP/input ratio across each site after normalization by the number of mapped reads to the genome assembly.

### De novo ChIP-seq assembly

We used kmer-based de novo assembly methods to detect CENP-A-enriched regions [20]. We trimmed reads using TrimGalore v0.4.4 (https://www.bioinformatics.babraham.ac.uk/projects/trim_galore/) and the settings “-gzip -length 35 -paired.” For the second replicate, we further subsampled reads to 100× coverage using Bbnorm (v37.54, https://sourceforge.net/projects/bbmap/) with the settings “threads = 24 prefilter = t target = 100” for the de novo assembly. We created de novo ChIP-seq contigs (ChIPtigs) with Spades v3.11.0 (-t 24 -careful–sc;[89]) for each replicate (Dryad repository files 2–6: https://doi.org/10.5061/dryad.rb1bt3j [37]). To calculate CENP-A enrichment, we mapped input and ChIP reads to the ChIPtigs. We masked duplicates using Picard MarkDuplicates (v2.12.0; http://broadinstitute.github.io/picard) and filtered low-quality reads with samtools (v1.7) using “-f 3 -F 4 -F 8 -F 256 -F 2048 -q 30” for the paired-end reads and “-q 30” for the single-end reads to keep high-quality reads (mapping quality ≥ 30 and properly paired). We calculated the enrichment *P* value using MACS (version 2.1.1.20160309; -q 0.01—call-summits; [90]). ChIPtigs were mapped back to our assembly using megablast BLAST 2.6.0+ [91] with default setting, and the best hits were chosen. We removed potentially misassembled ChIPtigs and adjusted the peak regions in the reference sequence using custom scripts. We identified 1,919, 16,310, 14,667, and 4,916 significantly CENP-A-enriched ChIPtig regions from 127,426, 268,663, 625,927, and 184,133 total de novo ChIPtigs for each replicate, respectively (S3 Table).

### Identification of candidate centromeric contigs

We identified candidate centromeric contigs in the new iso-1 assembly [19] based on organization: we looked for contigs containing complex DNA flanked by satellites with known centromeric and pericentric locations. We first generated the assembly with long-read sequence data, including PacBio [92] and nanopore reads (S4 Table; [93]). We filtered nanopore reads using Porechop and Filtlong (—min_length 500) to remove adaptors and short reads (https://github.com/rrwick/Porechop and https://github.com/rrwick/Filtlong). We assembled the nanopore and PacBio reads into a hybrid assembly using Canu v1.7 with default settings [94]. Our new hybrid PacBio-Nanopore assembly is less contiguous than our previous PacBio-only assembly despite using more reads (see Dryad repository file 7: https://doi.org/10.5061/dryad.rb1bt3j [37]; assembly size = 162,798,260 bp in 798 contigs; N50 = 5,104,646 bp). We thus decided to use our PacBio-only assembly [19], which has a greater representation of heterochromatin compared to previously published assemblies (see details in [19]). To ensure that we were not missing putative centromeric contigs, we looked for sequences with CENP-A-enriched repeats (based on our repeat analysis; Fig 1B and S1 Fig) in the error-corrected PacBio and nanopore reads and the hybrid assembly that were missing from our PacBio-only assembly. We were particularly interested in contigs containing repeat sequences that we identified as enriched in our ChIP data. To annotate contigs and unassembled corrected reads, we used RepeatMasker 4.06 [95] with Repbase 20150807 and settings “-species drosophila -s” to annotate interspersed repeats (described above) and Tandem Repeat Finder (TRF v4.09; [96]) to annotate tandem repeats. We extracted 19 nonredundant sequences from our new hybrid assembly and error-corrected reads with candidate centromeric repeats, including *dodeca*, *Prodsat*, AATAG, IGS^3cen^ (as determined by phylogenetic analysis below), and *G2/Jockey-3* sequences. We added these 19 candidates to our new PacBio-only assembly [19] (Dryad repository file 8: https://doi.org/10.5061/dryad.rb1bt3j [37]) to create the final version of our assembly. We polished the final assembly 10 times using Pilon (v1.22 [97]) with Illumina [98, 99] and long synthetic reads [100] (S6 Table; with settings “—mindepth 3—minmq 10—fix bases”). We annotated the finished assembly using our customized repeat library (-lib library.fasta -s) and RepeatMasker 4.06 [95] (Dryad repository file 9: https://doi.org/10.5061/dryad.rb1bt3j [37]). Additionally, we transferred gene annotations from Flybase r6.20 to our genome using BLAT [101] and CrossMap v0.2.5 [102] (Dryad repository file 10: https://doi.org/10.5061/dryad.rb1bt3j [37]).

### Peak calling

We mapped our input and ChIP reads and publicly available data [16] to our genome assembly with new candidate sequences [19] using bwa v0.7.15 [103]. We masked duplicates using Picard MarkDuplicates (v2.12.0; http://broadinstitute.github.io/picard) and filtered low-quality reads with samtools (v1.7) using “-f 3 -F 4 -F 8 -F 256 -F 2048 -q 30” for the paired-end reads and “-q 30” for the single-end reads to keep high-quality reads (mapping quality ≥ 30 and properly paired). We then called peaks using MACS (version 2.1.1.20160309; -q 0.01—call-summits; hereafter referred to as MACS peaks [90]) with the alignments. We report top 100 peaks with strongest signal from each replicate (fold-change column of S4 Table). We used IDR [104] to overlap the datasets and identify high confidence peaks (https://github.com/nboley/idr) between every replicate (IDR < 0.05 corresponding to an IDR score ≥ 540). Since there are many peaks with weak CENP-A enrichment in the comparison between R2 and R3 (16,870), we only chose 37 peaks—the average peak number of other comparisons (27–44)—with strongest signals for our figures (S5 Table).

### ChIP-qPCR

qPCR was performed using SYBR-green (Bio-Rad) on a CFX96 Real-Time System (Bio-Rad). Input or ChIP eluted DNA (1 μl) was used in each qPCR reaction. Melting curves were analyzed to ensure primer specificity. Only primers with reaction efficiencies within a linear dynamic range were used. The fold enrichment of centromeric DNA after immunoprecipitation of CENP-A chromatin compared to its level in the bulk input chromatin was calculated with the equation 100 × E^(Ct*input −* Ct*ip*)^, where E is the efficiency of the primer set. Enrichment values were normalized by the enrichment value of *RpL32* as a noncentromeric control. qPCR primer sets are listed in S7 Table.

### Transcription of centromeric *G2/Jockey-3* elements

Total RNA was extracted from three independent overnight collection of embryos (iso-1). Briefly, embryos were scooped from apple juice plates and rinsed with water in a mesh basket, dechorionated in 50% bleach for 3.5 min with gentle shaking, rinsed thoroughly with water, moved to a 1.5-ml microfuge tube, and resuspended in 300 μl of Trizol reagent (Sigma-Aldrich). Embryos were homogenized using a motorized pestle until the solution became clear (30–40 s). The homogenized solution was centrifuged at 13,000 rpm for 10 min at 4°C, and the clear supernatant was transferred to a new RNAse-free tube. RNA was isolated using the Direct-Zol RNA miniprep plus kit (Zymo Research) according to the manufacturers’ protocol. The RNA was eluted in 30 μl of RNAse-free water and quantified with a Nanodrop. A total of three consecutive Turbo DNase (Invitrogen) treatments, each followed by RNeasy Cleanup (Qiagen), were performed to remove DNA contamination.

Reverse transcription was performed using the iScript Select cDNA Synthesis kit (Bio-Rad) according to the manufacturer’s instructions. Briefly, 75 ng of total embryo RNA was used to make cDNA libraries using random priming in a 30 μl reaction. For the no-RT control, the reverse transcriptase was omitted from the reaction.

qPCR was performed as described for ChIP-qPCR using 1 μl of cDNA in each reaction and primers sets targeting *G2/Jockey-3* copies from each centromere (X-G2, 4-G2, Y-G2, 3-G2, 2-G2). Primers for *Actin5C* were used as a positive control for a highly expressed gene, whereas primers for the testis-specific gene, *Mst84Da*, were used as a control for a nonexpressed gene. The no-RT samples produced Ct values comparable to the negative nonexpressed control showing successful removal DNA.

Gene expression was analyzed as done by Schmittgen et al. [105] by determining the mean 2^-ΔCt^, where ΔCt is (Ct_G2/Jockey-3_ − Ct_Mst84Da_), from three biological replicates. Primer sets are listed in S7 Table.

### IF and FISH

#### S2 mitotic chromosome preparation

Preparation of mitotic chromosomes from Drosophila S2 cells was performed as described in [82]. Cells (2 × 10^5^) were treated with 0.5 μg/mL demecolcine solution (Sigma-Aldrich) and incubated at 25°C for 1 h to induce a mitotic arrest. Cells were pelleted (600g for 5 min) and resuspended in 250 μL 0.5% (w/v) sodium citrate for 8 min. Cells were loaded into cytofunnels and spun onto Superfrost Plus slides (VWR) at 1,200 rpm for 5 min using a Shandon Cytospin 4 (ThermoFisher). Cells were fixed for 10 min with 3.7% formaldehyde in PBS, 0.1% Triton X-100 (PBS-T). Slides were washed three times in PBS-T for 5 min and stored at 4°C until ready for use.

#### *D*. *melanogaster* and *D*. *simulans* mitotic chromosomes preparation

Preparation of mitotic spreads was carried out from iso-1 *D*. *melanogaster* flies (Bloomington Drosophila Stock Center stock no. 2057: y^1^; Gr22b^iso-1^ Gr22d^iso-1^ cn^1^ CG33964^iso-1^ bw^1^ sp^1^; MstProx^iso-1^ GstD5^iso-1^ Rh6^1^) and *D*. *simulans* (w501, gift of Andy Clark) in larvae following the method in [106] with minor modifications. Third instar larval brains from male larvae were dissected in PBS and immersed in 0.5% (w/v) sodium citrate for 8 min. Individual brains were fixed for 6 min in 6 μL of 45% acetic acid, 2% formalin (Sigma-Aldrich) on siliconized coverslips. Whole brains were applied to clean poly-L-lysine slides (ThermoFisher) and were manually squashed between coverslip and slide by pressing with the thumb. Slides were immersed in liquid nitrogen. Once bubbling stopped, the slides were removed from liquid nitrogen and the coverslip was immediately removed using a razor blade. Slides were immediately immersed in PBS and were either washed for 5 min before proceeding to IF or stored at 4°C in PBS until ready for use.

#### IF staining

For IF, slides were washed in PBS-T for 5 min. S2 cell slides were blocked in 5% milk in PBS-T for 30 min. Larval squashes were blocked in 1% BSA, PBS, 0.02% sodium azide for 30 min. Primary antibodies anti-CENP-A (larval brain slides: rabbit, 1:500, Active Motif; S2 cell slides: chicken, 1:1,000, [30]) and anti-CENP-C (larval brain slides: guinea pig, 1:500 [34]) were diluted in blocking solution and incubated on slides overnight at 4°C. Slides were washed three times for 5 min in PBS-T and incubated with secondary antibodies (Life Technologies Alexa-488, 546, or 647 conjugated, 1:500) diluted in blocking solution and incubated at room temperature for 1 h or overnight at 4°C. Slides were washed three times for 5 min in PBS-T.

#### Satellite FISH

Satellite FISH was performed following the protocol described in [107] with a few modifications. Slides were postfixed in 3.7% formaldehyde and PBS for 10 min, followed by a rinse in PBS and two 5-min washes in 2xSSC, 0.1% Tween-20 (2xSSC-T). Slides were washed once for 5 min in 50% formamide (Sigma-Aldrich), 2xSSC-T at room temperature, once for 20 min in 50% formamide (Sigma-Aldrich), 2xSSC-T at 60°C, and then cooled to room temperature. For FISH, 25 μL of hybridization mix containing 40 pmol of each probe (S11 and S12 Tables), 2xSSC-T, 10% dextran sulfate (Merck), 50% formamide, and 1 μL of RNase Cocktail (ThermoFisher) was applied to a 22 × 22-mm hybrislip (Electron Microscopy Sciences), mounted on the slide and sealed with paper cement. Slides were denatured at 92°C for 2.5 min and then incubated overnight at 37°C. Slides were washed in 2xSSC-T at 60°C for 20 min, followed by two 5-min washes in 2xSSC-T at room temperature and one 5-min wash in PBS. Slides were mounted in Slowfade Gold Reagent (Invitrogen) containing 1 μg/mL DAPI and sealed with nail polish.

#### Oligopaint FISH

Oligopaint FISH was performed as described above with the following modifications. Hybridization mix (25 μL) containing 10 pmol of Oligopaint, 2xSSC-T, 10% dextran sulfate (Merck), 60%–68% formamide (Sigma-Aldrich), and 1 μL RNase cocktail (ThermoFisher) was applied to a 22 × 22-mm hybrislip (Electron Microscopy Sciences), mounted on the slide, and sealed with paper cement. Slides were denatured at 92°C for 2.5 min in a thermocycler (Eppendorf) and incubated overnight at either 37°C or 42°C (see S10 Table for the percent of formamide and hybridization temperatures used). For fluorescence detection, 10 pmol of Alexa-488-labeled secondary oligos (see S13 Table) were applied either during the overnight hybridization or following posthybridization washes, in which 25 μL of 2xSSC, 30% formamide, 10 pmol of probe was applied to each slide and incubated at room temperature for 30 min. Slides were washed twice in 2xSSC, 40% formamide for 20 min, once in 2xSSC-T for 15 min, and once in PBS for 5 min. Slides were mounted as described above, and successful hybridization was checked under fluorescence microscope. Satellite probes were added after imaging by removing the coverslip with a razor blade; washing slides three times in 2xSSC-T for 5 min; applying 25 μL of 2xSSC, 30% formamide, 40 pmol of satellite probe to each slide; and incubating at 37°C for 1 h. Slides were washed once in 2xSSC-T at 60°C for 20 min, twice in 2xSSC-T for 15 min, and once in PBS for 5 min and mounted as described above.

#### *G2/Jockey-3* FISH

FISH for *G2/Jockey-3* was performed as described by Dimitri et al. [108]. Slides were dehydrated in an ethanol row (successive 3-min washes in 70%, 90%, and 100% ethanol) and allowed to air-dry completely. Probe mix (20 μL) containing 2xSSC, 50% formamide (Sigma-Aldrich), 10% dextran sulfate (Merck), 1 μL RNase cocktail (ThermoFisher), and 100 ng of DIG-labeled *G2* probe was boiled at 80°C for 8 min, incubated on ice for 5 min, and then applied to slides, covered with a glass coverslip, and sealed with paper cement. Sealed slides were denatured on a slide thermocycler for 5 min at 95°C and incubated at 37°C overnight. Slides were then washed three times for 5 min in 2xSSC, 50% formamide at 42°C, three times for 5 min in 0.1xSSC at 60°C, and then blocked in block buffer 1% BSA, 4xSSC, 0.1% Tween-20 at 37°C for 45 min. Slides were incubated with 50 μL of block buffer containing a fluorescein-labeled anti-DIG antibody (sheep, 1:100, Roche) for 60 min at 37°C. Slides were then washed three times for 5 min in 4xSSC, 0.1% Tween-20 at 42°C, and mounted as described above.

#### Preparation of extended chromatin fibers and IF-FISH

Extended chromatin fibers were prepared as described by Sullivan [42], with a few modifications. Three to four brains from third instar iso-1 wandering larvae (females were selected to avoid cross-centromere hybridization of our X^*Maupiti*^ and 4^*Lampedusa*^ Oligopaints with CenY, whereas males were used for Y^*Lipari*^) were dissected in 0.7% NaCl and dissociated in 250 μl 0.5% (w/v) sodium citrate containing 40 μg collagenase/dispase (Sigma-Aldrich) by incubating at 37°C for 10 min. This mixture was briefly vortexed, spun, and loaded into a single-chamber Shandon cytofunnel for centrifugation in a Shandon Cytospin 4 at 1,200 rpm for 5 min onto a clean polysine slide (ThermoFisher). After centrifugation, the slides were immediately immersed in a glass coplin jar containing lysis buffer (500 mM NaCl, 250 mM urea, 25 mM Tris-HCl [pH 7.4], 1% Triton X-100) for 13–15 min, following which the slides were gently removed at a steady speed of about 25–30 s per slide. Fibers were fixed in 4% formaldehyde solution and washed in PBS for 5 min. After washing, the slides were processed for IF-FISH.

Fibers were extracted in PBS-T for 10 min then incubated in a 1.5% BSA, PBS blocking solution for 30 min. Slides were incubated with an anti-CENP-A antibody (rabbit, 1:100, Active Motif) diluted in blocking solution overnight at 4°C in a humidified chamber. Slides were washed three times in PBS for 5 min and then incubated for 45 min with secondary antibodies (Cy5-conjugated donkey anti-rabbit, 1:500, Life Technologies) diluted in blocking buffer at room temperature, followed by three 5-min washes in PBS. Slides were postfixed in 3.7% formaldehyde, PBS for 10 min followed by one quick rinse and two 5-min washes in PBS. FISH was performed as described for 3D-FISH [107] with a few modifications. Slides were washed twice in 2xSSC-T at room temperature for 5 min, followed by denaturation in 50% formamide, 2xSSC-T at room temperature for 5 min, transferred at 60°C for 20 min, and then cooled to room temperature. Primary Oligopaint probes (10 pmol, except X^*Maupiti*^, which was 25 pmol) and 40 pmol of satellite DNA probes were each added to the slides in 25 μL of hybridization solution—2xSSCT, 60% or 68% (v/v) formamide (see S10 Table), 1 μL RNase cocktail (ThermoFisher), 10% dextran sulfate (Merck)—and sealed with a 22 × 22-mm hybrislip (Electron Microscopy Sciences) using rubber cement. Slides were then denatured at 92°C for 3 min on a slide thermocycler and allowed to hybridize overnight at 37°C or 42°C (see S10 Table) in a humidified chamber. Slides were washed once in 2xSSC-T for 15 min at 60°C, once in 2xSSC-T for 10 min at room temperature, and once in 0.2xSSC for 10 min at room temperature. Following the washes, 25 μL of hybridization mix containing 2xSSC, 30% formamide, 40 pmol fluor-labeled secondary oligo probes (see S13 Table) was added on to each slide and incubated for 45 min at room temperature, except for the X^*Maupiti*^ slides, in which the satellite probe was also added with the secondary Oligopaint probe. The slides were then washed once in 2xSSC-T at 60°C for 15 min, followed by one wash in 2xSSC-T and 0.2xSSC for 10 min at room temperature and mounted as described above.

For FISH with only satellite probes, posthybridization washes consisted of one wash in 2xSSC-T for 20 min at 60°C, followed by one wash with 2xSSC-T at room temperature for 10 min and two 5-min washes in 0.2xSSC at room temperature. Slides were mounted as described above.

For fiber measurement calibration, FISH using the 61C7 and 80C4 probes was performed using the conditions for Oligopaint FISH (see S10 Table for percent of formamide and hybridization temperatures used), whereas FISH using the *Rsp* probe was performed using the satellite FISH protocol.

#### Microscopy and image analysis

Image acquisition was done at 25°C using an Inverted Deltavision RT restoration Imaging System (GE) equipped with a Cool Snap HQ^2^ camera (Photometrics) and 100×/1.40 NA oil immersion lens (Olympus). Image acquisition and processing was performed using softWoRx software (GE). For mitotic chromosomes, 20 z-stacks were taken per image at 0.2 μm per slice. For fibers, 12–15 z-stacks were taken per image at 0.15 μm per slice. Images were deconvolved using the conservative method for 5 cycles. Maximum intensity projections were made using 3–5 z-stacks. Images were saved as Photoshop files and were scaled using Adobe Photoshop. Figure assembly was done using Adobe Illustrator.

Maximum intensity projections of individual fibers were analyzed to measure the signal length of various signals on fibers using the “measure distances” tool in Softworks (GE). Three calibration probes of known length (100 kb; see S10 Table for 80C4 and 61C7 Oligopaints; see Dryad repository file 11: https://doi.org/10.5061/dryad.rb1bt3j [37] for *Rsp* probe) were used to determine the degree of stretching in our experiments. At least 20 fibers for each probe were measured in all cases. Length measurements were visualized by scatter plot using Prism. These lengths were then used to determine the average stretching in kb/μm, and Student *t* test was used for statistical analyses.

We noticed that the variation in the measurements for the island Oligopaints was greater than what we observed for the probes used for calibration. We attribute this higher variation to the lower density of island Oligopaint probes (some of the island sequences that are shared among centromeres were not targeted by probes to increase specificity), which causes the signal to be weaker and less consistent than in standard Oligopaint FISH. It is also important to note that we analyzed fibers from a mixed population at different stages of the cell cycle, which could display differences in CENP-A signal. It is also possible that the stretching of the chromatin at the centromere is more variable than at noncentromeric regions.

#### Oligopaints design

Oligopaint libraries were designed using the OligoMiner pipeline [38, 109] with some variations. The genomic regions that showed significant enrichments of CENP-A via MACS and enriched ChIPtigs were targeted for Oligopaint design. The blockparse.py script (v1.3) using overlap mode was used to identify as many candidate probes as possible, with genome-targeting regions 35–41 bp long and a desired melting temperature of 42–47°C. Unlike standard Oligopaints design, the candidate probes were not aligned to the genome using Bowtie2 [110] or filtered with OutputClean.py, so that probes that align multiple times would not be discarded. Candidate probes with partial alignments of 18 bp–long kmers were filtered out using kmerfilter.py (v1.3) and Jellyfish [111], excluding any that matched 6 or more times to the genome. Probes were filtered further for least secondary structures using StructureCheck.py (v1.3) and NUPACK [112]. Finally, coverage and density of probes across the regions of interest and presence of densely clustered off-target alignments were manually checked by Bowtie2 alignment, filtering for different levels of mismatch to assess the effects of hybridization stringency.

For the design of control regions for length standards in chromatin fiber stretching measurements, in loci 80C4 (3L: 23, 047,118..23,147,118) and 61C7 (3L: 626,646..726,646), we used conventional Oligopaint design for nonrepetitive genomic regions. The blockparse.py script (v1.3) was used to identify candidate probes, with genome-targeting regions 35–41 bp long and a desired melting temperature of 42–47°C. Candidate probes were then aligned to the dm6 reference genome (with NNN masking of repetitive regions) using Bowtie2 and its output filtered using outputClean.py (v1.5.4) to keep only those probes that are predicted to thermodynamically only hybridize on target under the specific conditions used. Finally, candidate probes were then further analyzed through kmerFilter.py (v1.3) to reject any probes containing regions of microhomology to off-target sites and through StructureCheck.py (v1.3) to exclude any probes forming restrictive secondary structures.

Each oligo included universal primers at the 5′ and 3′ ends for PCR amplification and a library-specific barcode for both PCR amplification and FISH detection of each individual centromere set. One library per centromere was synthesized as a single chip by Custom Array.

#### Library amplification

Raw Oligopaint libraries were amplified in 100-μl reactions containing 10 μl KAPA Buffer A and 1 μl KAPA Taq from the KAPA Taq PCR Kit (Fisher Scientific), 1 μl of library, 0.4 mM dNTPs (Roche), and 2 μM of each universal primer (S14 Table) and amplified using the following cycles: 95°C for 5 min; 25 cycles of 95°C for 30 s, 58°C for 30 s, and 72°C for 15 s; and a final extension at 72°C for 5 min. Reactions were purified using DNA Clean & Concentrator-5 (Zymo Research) using the manufacturer’s protocol.

Sublibraries were amplified in two 100-μl reactions containing 10 μl KAPA Buffer A and 1 μl KAPA Taq from the KAPA Taq PCR Kit (Fisher Scientific), 0.5 ng of amplified library, 0.4 mM dNTPs, 0.4 μM of each sublibrary-specific primer (forward primers containing a 5′ Sec6 secondary oligo probe adapter sequence and reverse primers containing a 5′ T7 promoter sequence) and amplified using the following cycles: 95°C for 5 min; 35 cycles of 95°C for 30 s, 60°C for 30 s, and 72°C for 15 s; and a final extension at 72°C for 5 min. Reactions for individual sublibraries were pooled and purified as describe above. Sublibrary-specific primers are listed in S15 Table.

#### Oligopaint synthesis and purification

T7 RNA synthesis was performed in 40-μl reactions containing 4 μl 10x T7 Buffer, 4 μl each NTP, and 4 μl T7 Pol Mix from the MEGAscript T7 Transcription Kit (ThermoFisher), 2 μg of amplified sublibrary, and 2 μl RNaseOUT Ribonuclease Inhibitor (ThermoFisher). Reactions were incubated at 37°C for 20 h. cDNA synthesis was performed in 300- μl reactions containing the entire T7 RNA synthesis reaction, 10 μM sublibrary-specific forward primer, 1.6 mM dNTPs (Roche), 60 μl 5x RT Buffer and 4 μl Maxima H Minus Reverse Transcriptase from the Maxima H Minus Reverse Transcriptase kit (ThermoFisher), and 3 μl of RNaseOUT. Reactions were incubated at 50°C for 2 h, followed by heat inactivation at 85°C for 5 min. RNA hydrolysis was performed by adding 300 μl of 0.25 M EDTA, 0.5 M NaOH to cDNA synthesis reactions and incubating at 95°C for 5 min. Reactions were then put on ice. Oligopaints were purified using DNA Clean & Concentrator-100 (Zymo Research) following the manufacturer’s protocol, substituting 4.8 ml of 100% ethanol and 1.2 ml of Oligo Binding Buffer (Zymo Research) instead of the DNA Binding Buffer. Oligopaints were eluted using 150 μl mqH_2_O. The concentration of each Oligopaint was determined using a NanoDrop 2000c Spectrophotometer using the ssDNA setting. The molarity of each Oligopaint was calculated using the following formula:

eluate concentration [ng/μL] × (1 pmol/330 pg) × (1/Oligopaint length in nt) = Oligopaint molarity [μM]

#### *G2/Jockey-3* probe design

We designed a 1,643-bp *G2/Jockey-3* oligo against the consensus of the 3′ region of *G2/Jockey-3* elements found within most centromere contigs. A 5′ addition containing 5′-CAGT-3′ followed by universal forward primer binding sites separated by an XhoI cut site (5 ′-cacactccggtacgcacctgctcgagcagtgctcgttggcccacac-3′). A 3′ addition containing 5′-ACTG-3′ followed by universal reverse primer binding sites separated by a SpeI cut site (5′-agggtagtcgttgtagctcgactagtggtacgcccagaagcatccc-3′). The *G2/Jockey-3* sequence was ordered as a “custom gene” (IDTDNA.com) and synthesized in the pUCIDT (AMP) vector (pUCIDT-G2). The sequence of the insert is as follows (primer binding sites = *italicized*; restriction sites = **bold**; *G2/Jockey-3* sequence = CAPITALIZED).

5′-cagt*cacactccggtacgcacctg***ctcgag***cagtgctcgttggcccacac*CGGACGGCTCTTGGTGCCGCTCTGAAGCCGAAAGAGCTGAAGCGTTTGCAGATCACCTCCAGAATGCATTCACACCATTTGACAGATGCACTGGCGAAGAGCGTGCTGCAACCACCAGGTTCCTAGAGAGTCCATGTCCTCCTAGCCTGCCCATAGAGCCCGTCACCCCAGAAGAGGTTGCGCAAGAGTCGCCTCACTAAAGGCTAGCAAATCCCCAGGACTGGATCGCATCGACGCCACATCCCTTAAAATGCTGCCACCTCCCTGTTCCCAGTTGCTGGCCAACATATACAACAGATGCTTCTCACTAGGGTACTTCCCGAGATCATGGAAACGTGCAGAAGTCATTCTCATCCTCAAACCTGGAAAACCTGAAGCCAATCTTGCCTCATATAGACCGATTAGTCTGCTGGCAATCCTCTCCAAAATACTCGAAAGAGTATTTCTGCGCAGAGTGTTGCCAGTACTGGACGAGGCTGGACTGATCCCTGATCACCAGTTTGGCTTCAGGCGATCCCACGGAACACCCGAGCAATGCCACCGGCTCGTAGCACGCATCCTAGATGCATTCGAGAACAAACGATACTGTTCGGCCGTATTCCTGGATGTCAAGCAGGCGTTCGACAGAGTGTGGCATCCTGGACTCCTCTACAAACTCAAGTCCCACCTTCCCAGTTCCCACTATGCCCTACTCAAATCGTATACTGAAGGAAGAGAGTTCCAAGTGCGATGCGGTTCCTCAACCAGCACGACAAGGCCTATACGAGCCGGAGTACCTCAAGGCAGCGTCCTTGGTCCCATCCTCTACACCCTGTTTACAGCAGACCTCCCTATCATACCCTCCCGTTACCTCACAGCAGCCACCTATGCAGATGACACGGCGTTCCTTGCCACCGCAACAAACCCTCAACTAGCATCAGCCATCATCCAGAGGCAACTGGATGCATTGGATCCATGGCTGAAACGCTGGAACATCGTGATCAACGCTGATAAATCCTCCCACACCACCTTCTCTCTGCGCAGAGGAGAATGCCCCCCGGTCTCACTCGACGGCGACACAATCCCTACCTCCAGCACCCCCAAATATTTAGGGCTGACCCTGGACAGAAGGCTGACTTGGGGCCCCCACATCAACAGAAAGCGTATCCAGGCCAACATACGCCTAAAGCAACTCCACTGGCTCATCGGTAAAAAGTCCAAGCTGCGAGAGAAACTAAAGATTCTCGTCTACAAGACTATTCTCAAGCCAATCTGGACGTACGGAATTCAGCTGTGGGGCACTGCAAGCACATCACATAGAAGGAAGATCCAGCGATTTCAAAACAGATGTTTGAGAATAGTCTCCAACGCCCATCCCTACCACGAAAATTCCGCCATCCACGAGGAGCTCGGGATTCCATGGGTAGACGACGAAATCTACAGACACAGTGTGAGATATGCTAGCAGACTGGAGAACCACCACAACCACCTGGCCGTCAACCTTCTAGACCATAGCCAATCCCTAAGACGCCTGCAGAGAACGCACCCGCTTGACCTTACTCAACATACTTAATCATACTTAACCCCTACCCAAGTACACTCGATGTACTCCCCTTAAGTTAATGTTTCCCTCCAAAAAATTTAATTATTGTCCACTAGGACAG*gggatgcttctgggcgtacc***actagt***cgagctacaacgactaccct*cagt-3′

#### *G2/Jockey-3* DIG probe synthesis

pUCIDT-*G2* (500 ng) was digested using SpeI and XhoI restriction enzymes in 1x Cutsmart Buffer for 1 h at 37°C. The digest was run on a 1.0% SeaPlaque GTG agarose gel (Lonza), and a 1,689-bp band containing the *G2* sequence was gel extracted and purified using the PureLink Quick Gel Extraction Kit (Invitrogen). DIG-labeled *G2* probes were generated via PCR in 50-μl reactions consisting 0.09 ng of gel extracted *G2* DNA, 0.5 μM of forward and reverse primers from the Universal_2 primer set (see S14 Table), 1x HF Buffer, 1 unit of Phusion Polymerase (NEB), 0.2 μM dGTP, 0.2 μM dATP, 0.2 μM dCTP, 0.15 μM dTTP, 5 nM DIG-dUTP (Roche). Probe was synthesized using the following cycles: 98°C for 30 s, 30 cycles of 98°C for 10 s and 72°C for 1 min, and a final extension at 72°C for 5 min. Unpurified PCR product was used as a probe for FISH.

### Hi-C analysis

We used a publicly available Hi-C dataset from embryos (Gene Expression Omnibus accession number GSE103625) to provide additional support for our candidate centromeric contigs [41]. We mapped Hi-C sequence reads to our assembly and processed the output with the HiC-Pro pipeline [113] to obtain informative valid interaction pairs (default settings). We used a customized python script to count interactions between regions of interest and then normalized to the size of the regions (per 100 kb). To count interactions between different-sized windows, we used BEDTools [114] to create windows of specified sizes across the assembly. We established the euchromatin–heterochromatin boundaries in our assembly based on previous studies. For chromosome 2, 3, X, and Y, we transferred the euchromatin–heterochromatin boundary coordinates previously reported for *D*. *melanogaster* [115] to our assembly. For chromosome 4, we assigned the approximately 70 kb closest to the centromere in the assembled chromosome 4 as heterochromatin based on what was previously reported [116] and the rest of it as euchromatin. We then binned the genome into different regions based on their sequence content: centromere, proximal heterochromatin, distal heterochromatin, and euchromatin (S16 Table). We then classified interactions between centromeric contigs and the different categories based on their genomic region (e.g., centromere to proximal heterochromatin, centromere to distal heterochromatin, etc.). We reported the median count for each category and conducted data visualization and statistics in R.

We calculated the significance between different categories using a Kruskal-Wallis test by ranks with Dunn’s test for post hoc analysis and the pairwise Wilcoxon rank sum test with false discovery rate (FDR) correction [117] of type I error rates for multiple comparisons. We deemed a result to be significant only if both tests agree.

### Phylogenetic analyses of IGS and *G2/Jockey-3* elements

We extracted all IGS elements from the genome using BLAST v2.7.1 [91] with settings “-task blastn -num_threads 24 -qcov_hsp_perc 90” and custom scripts. We extracted the *G2/Jockey-3* sequences based on RepeatMasker annotations and custom scripts. We aligned and manually inspected *G2/Jockey-3* and IGS alignments using Geneious v8.1.6 [118] (see Dryad repository files 12 and 13: https://doi.org/10.5061/dryad.rb1bt3j [37]). We constructed maximum-likelihood phylogenetic trees for *G2/Jockey-3* and IGS using RAxML v.8.2.11 with settings “-m GTRGAMMA -T24 -d -p 12345 -# autoMRE -k -x 12345 -f a” [119]. We used the APE phylogenetics package in R [120] to plot the trees.

### *G2/Jockey-3* activity

We investigated whether *G2/Jockey-3* non-LTR retroelements have evidence for recent activity based on insertion polymorphism and expression. We examined RNA-seq reads from testes for evidence of *G2/Jockey-3* because of the enrichment of these elements on the Y chromosome. We mapped poly-A [121] and total RNA [122] (S6 Table) transcriptome data to our repeat library using HISAT 2.1.0 [123] and estimated read depth of uniquely mapped read using samtools (depth–Q10; v1.7 [124]).

## Supporting information

S1 Fig Enrichment of simple tandem repeats in CENP-A ChIP-seq across four replicates. Plot of normalized CENP-A/input for simple tandem repeats for each ChIP-seq replicate, sorted by median (red lines). Shown are only the simple tandem repeats with median CENP-A/input > 1 in all four CENP-A ChIP replicates (see details in S1 Table). The simple tandem repeats with fewer than 10 counts of input reads in any one replicate are not shown. CENP-A, centromere protein A; ChIP, chromatin immunoprecipitation; ChIP-seq, ChIP sequencing.
(TIF)

S2 Fig *G2* and *Jockey-3* correspond to the same non-LTR retroelement. A maximum-likelihood phylogenetic tree showing the relationship between *G2* and *Jockey-3* sequences in *D*. *melanogaster* genome and closely related species in the simulans clade (*D*. *simulans* and *D*. *sechellia*) and *D*. *yakuba*. In *D*. *melanogaster*, *G2* and *Jockey-3* are interleaved across the phylogeny and thus likely correspond to the same repeat type. We therefore refer to these elements collectively as *G2/Jockey-3* throughout the manuscript. (See Dryad repository files 13 and 15: https://doi.org/10.5061/dryad.rb1bt3j [37]). LTR, long terminal repeat.
(TIF)

S3 Fig Reproducibility of CENP-A ChIP enrichment among replicates in embryos and S2 cells. Locations of the top 100 strongest peaks for each ChIP experiment. (A) Plot of the location of top 100 strongest peaks for each ChIP experiment on the diagonal (see details in S4 Table). For the four replicate ChIP experiment in our OreR embryos, we examined the reproducibility of our experiments by first applying the IDR test and only keeping peaks with IDR ≤ 0.05. The number of these peaks is plotted below the diagonal. Between replicates 2 and 3, we found a total of 16,870 overlapping peaks, but 16,833 were weakly enriched relative to the overlapping peaks between other datasets because they are technical repeats with a shared library bias (Accel, see Materials and methods). We therefore only report the 37 strongest peaks (the average peak number of other comparisons between replicates). The IDR dataset comparisons are in S5 Table. We show the correlation between the CENP-A ChIP replicates above the diagonal. Plotted are the signal strength after IDR tests (normalized ChIP over input ratio from 1 to 1,000 on a log10 scale) with Spearman’s rho. The five contigs with the most consistent peaks within and among replicates correspond to the five centromeric candidates. (B) Plot of ChIP-seq data from S2 cells (this paper, [16, 82]) and an independent embryo CID–GFP (i.e., CENP-A–GFP) ChIP-seq dataset (see details in S4 Table; [16]; “5m” and “15m” represent different MNase treatments). The centromeric contigs are also CENP-A enriched in these independent datasets, with the exception of the X chromosome centromere contig. S2 cells lack a Y and are therefore not expected to have peaks on the Y candidate centromere contig. CENP-A, centromere protein A; ChIP, chromatin immunoprecipitation; ChIP-seq, ChIP sequencing; CID, centromere identifier; GFP, green fluorescent protein; IDR, irreproducible discovery rate; OreR, Oregon-R; S2, Schneider 2.
(TIF)

S4 Fig CENP-A occupies DNA sequences within putative centromere contigs. Organization of each CENP-A-enriched island corresponding to centromere candidates: (A) X centromere, (B) centromere 4; (C) Y centromere; (D) centromere 3; (E) centromere 2. Different repeat families are color coded (see legend; note that *Jockey* elements are shown in one color even though they are distinct elements). The normalized CENP-A enrichment over input (plotted on a log scale) is shown for three replicates (replicate 2 is in Fig 2) colored in gray for simple repeats and black for complex island sequences. Although the mapping quality scores are high in simple repeat regions, we do not use these data to make inferences about CENP-A distribution (see main text for details). The coordinates of the significantly CENP-A-enriched ChIPtigs mapped to these contigs (black) and the predicted ChIP peaks (orange) are shown below each plot. See Fig 2 and S3 and S4 Tables. CENP-A, centromere protein A; ChIP, chromatin immunoprecipitation.
(TIF)

S5 Fig ChIP-qPCR validation of CENP-A-enriched regions. (A) Diagram showing putative centromere contigs showing the locations of CENP-A ChIPtigs in black and CENP-A MACS peaks in orange as in Fig 2. Locations of contig-specific qPCR primer binding sites are shown by magenta arrows. (B) Graph showing our ChIP-qPCR results using these primers. The enrichment is calculated relative to the input and is normalized by the *RpL32* promoter region as a noncentromeric control. (C) Graph showing our ChIP-qPCR results using primers targeting other regions that showed CENP-A enrichment but that were not in our contigs. Again, the enrichment is calculated relative to the input and is normalized by *RpL32* promoter as a noncentromeric control. We did not observe a robust CENP-A enrichment at these sites. The underlying data can be found in S2 Data. CENP-A, centromere protein A; ChIP, chromatin immunoprecipitation; qPCR, quantitative PCR.
(TIF)

S6 Fig Relative depth of PacBio reads across centromeric contigs. PacBio reads were mapped to the genome using Minimap (v 2.11) and the setting “-ax map-pb.” Shown are (A) X centromere, (B) centromere 4, (C) Y centromere, (D) centromere 3, and (E) centromere 2. The depth of only the high-quality mapped reads (mapped Q ≥ 30) was estimated for each position and normalized by the median depth of other genomic regions (98.32× for autosomes and 49.16× for sex chromosomes) to get relative depth. The relative depths of the TE-rich islands are close to 1, whereas the depth of the flanking simple satellites is uneven, with some regions > 1 and some < 1. We therefore exclude simple repeats from any assembly-based analyses and color these regions gray in Fig 2 and S4 Fig to indicate that caution should be used in interpreting these regions of the assembly. The underlying data can be found in S2 Data. TE, transposable element.
(TIF)

S7 Fig Satellite FISH on iso-1 larval brain mitotic spreads. IF-FISH using an anti-CENP-C antibody (green) and satellite FISH probes in the following combinations: (A) AAGAT (magenta) and AAGAG (blue) with a high-contrast inset of AAGAT on the X chromosome; (B) *Prodsat* (magenta) and AAGAG (blue); (C) AATAG (magenta) and AAGAG (blue) with AATAG blocks identified by white (small block) and yellow (large block) arrows; (D) *Prodsat* (magenta) and *dodeca* (blue); (E) AATAT (magenta) and *SATIII* (blue); (F) AATAT (magenta) and AAGAG (blue); (G) AATAG (magenta) and *Prodsat* (blue) with AATAG blocks identified by white (small block) and yellow (large block) arrows. DAPI is shown in gray. The underlying data can be found in S2 Data. Bar 5 μm. CENP-C, centromere protein C; FISH, fluorescence in situ hybridization; IF, immunofluorescence; *Prodsat*, Prod satellite.
(TIF)

S8 Fig Satellite FISH on S2 cell mitotic spreads. IF-FISH using an anti-CENP-A antibody (green) and satellite FISH probes in the following combinations: (A) AATAT (magenta) and *SATIII* (blue); (B) *dodeca* (magenta) and *Prodsat* (blue); (C) AATAG (magenta) and *Prodsat* (blue) with a high-contrast inset of AATAG and *Prodsat* on cf(2R); (D) AAGAG (magenta) and *Prodsat* (blue); (E) AAGAG (magenta) and AATAG (blue) with a high-contrast inset of AATAG on chromosome 3; (F) AAGAT (magenta) and AAGAG (blue). DAPI is shown in gray. See also S18 Table. Bar 5 μm. CENP-A, centromere protein A; cf(2R), centric fragment of chromosome 2R; FISH, fluorescence in situ hybridization; IF, immunofluorescence; *Prodsat*, Prod satellite; S2, Schneider 2.
(TIF)

S9 Fig Transcription of *G2/Jockey-3* elements. (A) Shown is the plot of the normalized reads depth from uniquely mapped reads (mapping quality ≥ 10) across the *G2/Jockey-3* consensus element obtained from mapping total and poly-A RNA-seq data from testes [121, 122] to our repeat library. (B) Quantitative RT-PCR analysis of total RNA extracted from three independent overnight embryo collections. Expression levels were compared to the negative control gene Mst84Da (testis-specific). The *G2/Jockey-3* copies surveyed on centromere (“Cen”) X, 4, and 3 but not Y and 2 show low levels of transcription compared to the housekeeping gene Actin. Although the primers (S7 Table) are specific for each centromere, the primer sets could amplify *G2/Jockey-3* copies not included in our assembly. Error bars = SD. The underlying data for this figure can be found in S2 Data. Mst84Da, Male-specific RNA 84Da; RNA-seq, RNA sequencing; RT-PCR, reverse-transcription PCR.
(TIF)

S10 Fig Relationship of IGS in *D. melanogaster* and closely related species of the simulans clade (*D. simulans* and *D. sechellia*) and *D. yakuba*. Maximum-likelihood phylogenetic tree of all individual IGS sequences found in the *D*. *melanogaster* genome with related outgroups (sequence alignment is in the Dryad repository file 12: https://doi.org/10.5061/dryad.rb1bt3j [37]). Node support is only shown for key nodes in the tree (complete tree is in the Dryad repository file 14: https://doi.org/10.5061/dryad.rb1bt3j [37]). All centromeric IGS sequences appear to have a single origin: they duplicated from sex-linked IGS interspersed at the rDNA loci at some time near the divergence of the simulans clade and *D*. *melanogaster*. IGS repeats in blue (extra) are similar to the IGS at 3^*Giglio*^ but are on small contigs, tig00022795 and id = 102159_0. Contig tig00022795 is also moderately enriched in CENP-A. CENP-A, centromere protein A; IGS, intergenic spacer of the ribosomal genes.
(TIF)

S11 Fig Genomic TE distribution across chromosomes. Distribution of TEs (represented by different colors) along the following chromosomes: (A) chromosome 2, (B) chromosome 3, (C) chromosome 4, (D) chromosome X, and (E) chromosome Y. Contigs from each chromosome were concatenated in order with an arbitrary insertion of 100 kb of “N.” Distances along the x-axis are approximate. The order and orientation of the Y chromosome contigs are based on gene order (see [19]). Each triangle corresponds to one TE, for which filled shapes indicate full-length TEs and open shapes indicate truncated TEs. The vertical gray bars represent the arbitrary 100-kb window inserted between contigs, indicating where we have gaps in our assembly. The centromere positions are set to 0 for each chromosome. Chromosomes are not drawn to scale (chromosome 4 and Y are enlarged). We show the genomic distribution of a sample of TEs enriched in CENP-A according to our ChIP-seq analysis (all except *PROTOP*). *PROTOP* are DNA transposons that have not been recently active, and their distribution is primarily in heterochromatin. *TART* elements are non-LTR retroelements highly enriched at telomeres and are also moderately CENP-A enriched. *DM1731* is a retroelement moderately enriched for CENP-A but not enriched in the centromere islands. *Doc2*, *G*, *Jockey-1*, and *G2/Jockey-3* are CENP-A enriched non-LTR retroelements abundant in the centromere islands (see S2 and S9 Tables). CENP-A, centromere protein A; ChIP, chromatin immunoprecipitation; ChIP-seq, ChIP sequencing; FISH, fluorescence in situ hybridization; IF, immunofluorescence; LTR, long terminal repeat; *TART*, Telomere-associated retrotransposon; TE, transposable element.
(TIF)

S12 Fig Oligopaint FISH on larval brain mitotic spreads from iso-1 flies. IF-FISH using an antibody for CENP-C (green), centromere Oligopaint FISH probes (magenta), and FISH probes for centromeric satellites (blue) in the following combinations: (A) *Maupiti* (X; magenta) and AAGAG (blue); (B) *Lampedusa* (4; magenta) and AAGAT (blue); (C) *Lipari* (Y; magenta) and AATAT (blue); (D) *Giglio* (3; magenta) and *dodeca* (blue). White boxes show the separate signals at the targeted centromeres. Yellow boxes show centromeric hybridizations at other centromeres. DAPI is shown in gray. Bar 5 μm. The underlying data for this figure can be found in S2 Data. CENP-C, centromere protein C; FISH, fluorescence in situ hybridization; IF, immunofluorescence.
(TIF)

S13 Fig Oligopaint FISH on S2 cell mitotic spreads. IF-FISH using an antibody for CENP-A (green) and centromere Oligopaint FISH probes designed to target centromere contigs (magenta). (A) *Maupiti* (X), (B) *Lampedusa* (4), (C) *Lipari* (Y), (D) *Giglio* (3). The “Signal Adjusted” panels in (A) and (B) show high-contrast Oligopaint hybridization for visualization of weak foci. Bar 5 μm. See also S18 Table. CENP-A, centromere protein A; FISH, fluorescence in situ hybridization; IF, immunofluorescence; S2, Schneider 2.
(TIF)

S14 Fig Quantification of interactions between centromeres and different genomic regions by Hi-C. Plots showing intra- and interchromosomal interactions between regions in Hi-C data from: (A) stage 16 embryos (end of embryogenesis) and (B) embryonic cycles 1–8 (before zygotic genome activation; data from [41]). The different colors indicate interactions with individual centromeres of all chromosomes. Centromere–centromere interactions are significantly more frequent than interactions between centromeres and distal heterochromatin, interdistal heterochromatin, and euchromatin and marginally more significant than centromere–interproximal heterochromatin interactions. ****adjusted *P* < 0.0001; *adjusted *P* < 0.02, pairwise Wilcoxon rank sum test with FDR correction; Kruskal-Wallis test by ranks with Dunn’s test for post hoc analysis. The underlying data for this figure can be found in S2 Data. FDR, false discovery rate.
(TIF)

S15 Fig Calibration of extended chromatin fiber stretching. Stretched chromatin fibers from female third instar larval brain cells using the following probes: (A) *Rsp* locus (heterochromatic; approximately 100 kb; green); (B) 100-kb Oligopaint for a heterochromatic region on chromosome 3L (80C4; magenta); (C) 100-kb Oligopaint for a euchromatic region approximately 600 kb from the telomere of chromosome 3L (61C7; cyan). Arrows show the region of the fiber that was measured. Bar 5 μm. (D) Scatterplot showing the quantification of fiber lengths. Mean lengths were used to estimate the size in kb (approximately 10 kb/1 μm). Error bars show the standard deviation. *P* = 0.085 (n.s.) for each pair of measurements compared (two-tailed *t* test). The underlying data can be found in S2 Data. n.s., not significant; *Rsp*, *Responder*.
(TIF)

S16 Fig Organization of the X centromere. (A-G) Examples of fibers visualized with IF with anti-CENP-A antibody (green), FISH with Oligopaints for *Maupiti* (magenta), and AAGAG probe (cyan) on female third instar larval brain cells. DAPI is shown in gray. CENP-A occupies *Maupiti* and the AAGAG satellite. We observed some variation in FISH signals and *Maupiti* and CENP-A domain lengths, likely because of the efficiency of Oligopaint binding and variable stretching in this region. Arrows show the region of the fiber that was measured. (H) Scatterplot showing the quantification of the length of *Maupiti* FISH and CENP-A IF signals. Error bars show the standard deviation. *N* = 24 fibers. Bar 5 μm. The underlying data for this figure can be found in S2 Data. CENP-A, centromere protein A; FISH, fluorescence in situ hybridization; IF, immunofluorescence.
(TIF)

S17 Fig Organization of centromere 4. (A-G) Examples of fibers visualized by IF with anti-CENP-A antibody (green), FISH Oligopaint FISH for *Lampedusa* (magenta), and AAGAT probe (cyan). DAPI is shown in gray. CENP-A occupies predominantly the island *Lampedusa*. Arrows show the region of the fiber that was measured. (H) Scatterplot showing the quantification of the length of *Lampedusa* FISH and CENP-A IF signals. Error bars show the standard deviation. *N* = 25 fibers. Bar 5 μm. The underlying data for this figure can be found in S2 Data. CENP-A, centromere protein A; FISH, fluorescence in situ hybridization; IF, immunofluorescence.
(TIF)

S18 Fig Organization of the Y centromere. (A-E) Examples of fibers visualized by IF with anti-CENP-A antibody (green), FISH with Oligopaints for *Lipari* (magenta). DAPI is shown in gray. We did not include satellite FISH because no centromeric satellites are known for the Y. Note that the Oligopaints only target part of *Lipari* (see Fig 5). CENP-A is observed occupying sequences beyond the Oligopaint region, likely over the remaining part of the island. Arrows show the region of the fiber that was measured. (F) Scatterplot showing the quantification of the length of *Lipari* FISH and CENP-A IF signals. Error bars show the standard deviation. *N* = 19 fibers. Bar 5 μm. The underlying data can be found in S2 Data. CENP-A, centromere protein A; FISH, fluorescence in situ hybridization; IF, immunofluorescence.
(TIF)

S19 Fig Organization of centromere 3. (A-E) Examples of fibers visualized by IF with anti-CENP-A antibody (green), FISH with Oligopaints for *Giglio* (magenta), and a probe for the centromere 3–specific *dodeca* satellite (cyan). DAPI is shown in gray. CENP-A occupies primarily *Giglio* and a small stretch of *dodeca* satellite. Note that the binding of the *dodeca* (an LNA probe) is quite variable between fibers and results in several gaps that could be a result of the higher stringency conditions needed for *Giglio* Oligopaint FISH. Arrows show the region of the fiber that was measured. (F) Scatterplot showing the quantification of the length of *Giglio* FISH and CENP-A IF signals. Error bars show the standard deviation. *N* = 30 fibers. Bar 5 μm. The underlying data can be found in S2 Data. CENP-A, centromere protein A; FISH, fluorescence in situ hybridization; IF, immunofluorescence; LNA, locked nucleic acid.
(TIF)

S20 Fig Tracking of longer centromere 3 fibers reveals a second region containing CENP-A on dodeca. (A-D) Examples of longer fibers tracked along *dodeca* from the experiment in S19 Fig, visualized by IF with anti-CENPA antibody (green), Oligopaint FISH for *Giglio* (magenta), and FISH with *dodeca* probe (cyan). DAPI is shown in gray. Note the presence of *Giglio* signal on the *dodeca* CENP-A region. Multiple, overlapping panels were often acquired to follow an individual fiber. Panels were then cropped and juxtaposed in the figure, with white lines showing the separate images. White boxes show the CENP-A domain on *Giglio*, and yellow boxes show the smaller domain on *dodeca*. *N* = 5 (these are rare fibers to find in our preparations because of their length). Bar 5 μm. CENP-A, centromere protein A; FISH, fluorescence in situ hybridization; IF, immunofluorescence.
(TIF)

S21 Fig Organization of the centromere 2. (A-D) Examples of fibers visualized with IF with anti-CENP-A antibody (green) and FISH with satellites. DAPI is shown in gray. (A-B) Examples of fibers showing colocalization of CENP-A (green) with *Prodsat* (magenta) and AATAG (cyan). (C-D) Examples of fibers with AAGAG (cyan) and *Prodsat* (magenta). (E) Example of fiber with AAGAG (magenta) and AATAG (cyan). We propose that *Capri* is located between flanking blocks of AAGAG and AATAG satellites that reside very close to where the *Prodsat* begins. Arrows show the region that was measured for each fiber. (F) Scatterplot of CENP-A IF signal lengths. (G) Model for the organization of centromere 2 showing a possible location of *Capri*. Error bars show the standard deviation. *N* = 18 fibers. Bar 5 μm. The underlying data can be found in S2 Data. CENP-A, centromere protein A; FISH, fluorescence in situ hybridization; IF, immunofluorescence; *Prodsat*, Prod satellite.
(TIF)

S1 Table Enrichment of simple tandem repeats in kseek analyses. We used kseek [125] to estimate read counts for each kmer and normalized these read counts using the total mapped reads for each dataset (ChIP and input). We identified CENP-A-enriched kmers using the ratio of normalized counts for each ChIP experiment and its corresponding input. The enriched kmers reflect simple tandem repeats enriched in CENP-A discussed in the main text and S1 Fig. Fig 1 summarizes kmers with satellite repeats associated with centromeres. CENP-A, centromere protein A; ChIP, chromatin immunoprecipitation.
(XLSX)

S2 Table Raw and normalized counts of reads mapped to the complex repeats. Rows correspond to complex repeat families (TEs and complex satellites), with the counts per family in the ChIP and input reads from every dataset. We calculated enrichment for each repeat type by normalizing by total mapped reads for each dataset and taking the ratio of normalized values for each ChIP and its corresponding input. ChIP, chromatin immunoprecipitation; TE, transposable element.
(XLSX)

S3 Table ChIPtigs with peaks from MACS. We mapped all ChIP-seq data to the de novo assembled ChIPtigs and called peaks using MACS with high-quality reads (mapping quality ≥ 30 and masked PCR duplicates). We also mapped ChIPtigs to the genome to determine its genomic location and assigned repeat IDs based on BLAST results.
(XLSX)

S4 Table Peaks called by mapping to the genome assembly and MACS. We mapped the ChIP and input reads to our genome assembly and used the high-quality reads (mapping quality ≥ 30 and masked PCR duplicates) to call ChIP peaks with MACS. We show the peak locations for each dataset. ChIP, chromatin immunoprecipitation.
(XLSX)

S5 Table IDR tests between different replicates from OreR ChIP-seq. We used IDR to compare MACS peaks from different ChIP-seq replicates. We show the statistics for shared peaks from each comparison. ChIP-seq, chromatin immunoprecipitation sequencing; IDR, irreproducible discovery rate; OreR, Oregon-R.
(XLSX)

S6 Table Summary of all sequencing datasets used in this study. We list reads and mapping summaries of all Illumina and long-read datasets generated in this paper or downloaded from NCBI’s SRA.
(XLSX)

S7 Table List of qPCR primers. List of primers used for qPCR in this study. The centromere contig that each target is associated with (X, 4, Y, 3, and 2) is designated in the “Centromere” column. Note that in silico PCR for the 3_G2 primers predicted three specific products from centromere 3 as well as two products on contig tig00022795 and additional nonspecific products from the X chromosome when three or more mismatches are allowed all of the same 145-bp size. qPCR, quantitative PCR.
(XLSX)

S8 Table Noncentromeric overlapping peaks from MACS in the OreR embryo ChIP replicates. We listed peaks outside canonical centromeres with any agreement between replicate ChIP experiments (IDR ≤ 0.05). We also report any genes or repeat annotations that overlap the MACS peaks. Note that there is no general enrichment in *G2/Jockey-3* outside of the centromeric islands. ChIP, chromatin immunoprecipitation; IDR, irreproducible discovery rate; OreR, Oregon-R.
(XLSX)

S9 Table Statistical analysis of TE distributions. We show the copy numbers of TEs in different genomic regions. The sums of base pairs in the assembly size in centromeres (432,440 bp), pericentromeric heterochromatin (37,089,066 bp), and other regions (118,457,213 bp) were used to compute the distribution statistics of TEs. We created a 2-by-2 contingency table for each TE comparing observed to expected (based on the sum of bp) for each comparison: centromere to heterochromatin (“cen-het”) regions or centromeres to whole genome (“cen-genome”). We computed a Fisher’s exact test with FDR correction to get adjusted *P* values. *G2/Jockey-3*, *G*, *Doc2*, and *Jockey-1* are significantly enriched in centromeres relative to other heterochromatic regions and to the whole genome. Asterisk signs show that *TART* and *ProtoP* are significantly underrepresented in centromeres relative to other heterochromatic regions. FDR, false discovery rate; FISH, fluorescence in situ hybridization; *TART*, Telomere-associated retrotransposon; TE, transposable element.
(XLSX)

S10 Table Oligopaint hybridization conditions. Hybridization conditions used for FISH with specific Oligopaints. FISH, fluorescence in situ hybridization.
(XLSX)

S11 Table Labeled satellite probes. Information on the fluors used and sequences of satellite FISH probes used in this report. * = “+N” designates the incorporation of an LNA. FISH, fluorescence in situ hybridization; LNA, locked nucleic acid.
(XLSX)

S12 Table Unlabeled satellite probes. Information on the 5′ secondary oligo adapter site and sequence of satellite probes used in this report.
(XLSX)

S13 Table Secondary Oligo probes. Sequence and fluors of secondary oligo probes used for fluorescence detection of Oligopaints and unlabeled satellite probes.
(XLSX)

S14 Table Universal primers. List of primer sets used for library amplification and G2 probe synthesis.
(XLSX)

S15 Table Sublibrary-specific primers. List of primer sets used for sublibrary amplification and Oligopaint synthesis.
(XLSX)

S16 Table Chromatin status assignments for contigs. We assigned contigs from the assembly to a chromosome and a chromatin status (heterochromatin/euchromatin, etc., based on [115, 116]; see Materials and methods). Blank cells indicate that a region could not be assigned.
(XLSX)

S17 Table Overlap between normal and CENP-A overexpression S2 cells. We compared the MACS peaks shared between “normal” S2 (this study) and S2 with CENP-A overexpression using the IDR test. Some noncentromeric regions should have more CENP-A enrichment after CENP-A overexpression; however, only four peaks have IDR ≤ 0.05. None of these peaks have *G2/Jockey-3*. CENP-A, centromere protein A; IDR, irreproducible discovery rate; S2, Schneider 2.
(XLSX)

S18 Table S2 cell FISH quantification. Percentage of probe signals that overlap with different cytological locations (“C”: centromere; “P”: pericentromere; “H”: heterochromatin, and “N”: number of spreads analyzed) in S2 cells. The underlying data can be found in S2 Data. FISH, fluorescence in situ hybridization; S2, Schneider 2.
(XLSX)

S19 Table S2 cell satellite locations. Summary of the locations of satellite repeats determined by IF-FISH on S2 cell chromosomes X, X;4, 2, cf(2R), cf(2L), 3, 4, and 4^s^, using an anti-CENP-A antibody to mark the centromere. Locations were designated as centromeric (“Cen”), pericentric (“Peri”), or heterochromatic (“Het”). See also S18 Table. 4^s^, small chromosome 4; CENP-A, centromere protein A; cf(2L), centric fragment of chromosome 2L; cf(2R), centric fragment of chromosome 2R; S2, Schneider 2; X;4, Robertsonian translocation between chromosomes X and 4.
(XLSX)

S1 Data Underlying data for all main figures. (XLSX)

S2 Data Underlying data for all figures in Supporting information. (XLSX)

S3 Data Oligopaints sequences and information for centromeres X, 3, 4, and Y. The columns indicate the centromere contig ID, start and end coordinates of sequence, followed by the oligo sequence, and the melting temperature (all.oligos.cen.islands). Included are also the same Oligopaint sequences with 5' and 3' extensions containing the universal primer followed by library-specific barcodes (oligos.with.adaptors).
(XLSX)

S1 Text Description of results related to the figures in Supporting information. (DOCX)

## Abbreviations

BAC: bacterial artificial chromosome
Cen2: centromere 2
Cen3: centromere 3
Cen4: centromere 4
CenH3: centromeric histone H3
CENP-A: centromere protein A
CENP-C: centromere protein C
CenX: X centromere
CenY: Y centromere
ChIP: chromatin immunoprecipitation
ChIP-seq: ChIP sequencing
Cid: centromere identifier
CRM: centromeric retrotransposons of maize
CRR: centromeric retrotransposons of rice
CRW: centromeric retrotransposons of wheat
ETS: external transcribed spacer
FISH: fluorescence in situ hybridization
GFP: green fluorescent protein
IDR: irreproducible discovery rate
IF: immunofluorescence
IGS: intergenic spacer of the ribosomal genes
ITS: internal transcribed spacer
LAVA: LINE-Alu-VNTR-Alu-like
LINE: long interspersed nuclear element
LTR: long terminal repeat
n/a: not applicable
*Prodsat*: Prod satellite
qPCR: quantitative PCR
rDNA: ribosomal DNA
*Rsp*: *Responder*
RT-qPCR: reverse-transcription qPCR
S2: Schneider 2
TE: transposable element

## References

[1] Mendiburo MJ, Padeken J, Fulop S, Schepers A, Heun P. Drosophila CENH3 is sufficient for centromere formation. Science. 2011;334(6056):686–90. 10.1126/science.1206880 . 22053052

[2] McKinley KL, Cheeseman IM. The molecular basis for centromere identity and function. Nat Rev Mol Cell Biol. 2016;17(1):16–29. 10.1038/nrm.2015.5 . 26601620 PMC8603311

[3] Allshire RC, Karpen GH. Epigenetic regulation of centromeric chromatin: old dogs, new tricks? Nat Rev Genet. 2008;9(12):923–37. 10.1038/nrg2466 19002142 PMC2586333

[4] Pidoux AL, Allshire RC. Kinetochore and heterochromatin domains of the fission yeast centromere. Chromosome Res. 2004;12(6):521–34. 10.1023/B:CHRO.0000036586.81775.8b . 15289660

[5] Ohzeki J, Bergmann JH, Kouprina N, Noskov VN, Nakano M, Kimura H, et al Breaking the HAC Barrier: histone H3K9 acetyl/methyl balance regulates CENP-A assembly. EMBO J. 2012;31(10):2391–402. 10.1038/emboj.2012.82 22473132 PMC3364751

[6] McNulty SM, Sullivan LL, Sullivan BA. Human Centromeres Produce Chromosome-Specific and Array-Specific Alpha Satellite Transcripts that Are Complexed with CENP-A and CENP-C. Dev Cell. 2017;42(3):226–40 e6. 10.1016/j.devcel.2017.07.001 28787590 PMC5568664

[7] Kasinathan S, Henikoff S. Non-B-Form DNA Is Enriched at Centromeres. Mol Biol Evol. 2018;35(4):949–62. 10.1093/molbev/msy010 29365169 PMC5889037

[8] Jain M, Olsen HE, Turner DJ, Stoddart D, Bulazel KV, Paten B, et al Linear assembly of a human centromere on the Y chromosome. Nat Biotechnol. 2018;36(4):321–3. 10.1038/nbt.4109 29553574 PMC5886786

[9] Wolfgruber TK, Nakashima MM, Schneider KL, Sharma A, Xie Z, Albert PS, et al High Quality Maize Centromere 10 Sequence Reveals Evidence of Frequent Recombination Events. Front Plant Sci. 2016;7:308 10.3389/fpls.2016.00308 27047500 PMC4806543

[10] Hoskins RA, Carlson JW, Wan KH, Park S, Mendez I, Galle SE, et al The Release 6 reference sequence of the *Drosophila melanogaster* genome. Genome research. 2015 10.1101/gr.185579.114 . 25589440 PMC4352887

[11] Blower MD, Sullivan BA, Karpen GH. Conserved organization of centromeric chromatin in flies and humans. Dev Cell. 2002;2(3):319–30. 11879637 10.1016/s1534-5807(02)00135-1 PMC3192492

[12] Garavis M, Mendez-Lago M, Gabelica V, Whitehead SL, Gonzalez C, Villasante A. The structure of an endogenous Drosophila centromere reveals the prevalence of tandemly repeated sequences able to form i-motifs. Sci Rep. 2015;5:13307 10.1038/srep13307 26289671 PMC4542561

[13] Le MH, Duricka D, Karpen GH. Islands of complex DNA are widespread in Drosophila centric heterochromatin. Genetics. 1995;141(1):283–303. 8536977 10.1093/genetics/141.1.283 PMC1206727

[14] Sun X, Le HD, Wahlstrom JM, Karpen GH. Sequence analysis of a functional Drosophila centromere. Genome research. 2003;13(2):182–94. 10.1101/gr.681703 12566396 PMC420369

[15] Sun X, Wahlstrom J, Karpen G. Molecular structure of a functional Drosophila centromere. Cell. 1997;91(7):1007–19. Epub 1998/01/15. 9428523 10.1016/s0092-8674(00)80491-2 PMC3209480

[16] Talbert P, Kasinathan S, Henikoff S. Simple and Complex Centromeric Satellites in Drosophila Sibling Species. Genetics. 2018 Epub 2018/01/07. 10.1534/genetics.117.300620 . 29305387 PMC5844345

[17] Cleveland DW, Mao Y, Sullivan KF. Centromeres and Kinetochores. Cell. 2003;112(4):407–21. 10.1016/s0092-8674(03)00115-6 12600307

[18] Klein SJ, O'Neill RJ. Transposable elements: genome innovation, chromosome diversity, and centromere conflict. Chromosome Res. 2018;26(1–2):5–23. 10.1007/s10577-017-9569-5 29332159 PMC5857280

[19] Chang CH, Larracuente AM. Heterochromatin-Enriched Assemblies Reveal the Sequence and Organization of the *Drosophila melanogaster* Y Chromosome. Genetics. 2019;211(1):333–48. 10.1534/genetics.118.301765 . 30420487 PMC6325706

[20] He X, Cicek AE, Wang Y, Schulz MH, Le HS, Bar-Joseph Z. De novo ChIP-seq analysis. Genome Biol. 2015;16:205 10.1186/s13059-015-0756-4 26400819 PMC4579611

[21] Hanlon SL, Miller DE, Eche S, Hawley RS. Origin, Composition, and Structure of the Supernumerary B Chromosome of *Drosophila melanogaster*. Genetics. 2018 10.1534/genetics.118.301478 . 30249684 PMC6283169

[22] Koryakov DE, Zhimulev IF, Dimitri P. Cytogenetic analysis of the third chromosome heterochromatin of *Drosophila melanogaster*. Genetics. 2002;160(2):509–17. 11861557 10.1093/genetics/160.2.509 PMC1461961

[23] Andreyeva EN, Kolesnikova TD, Demakova OV, Mendez-Lago M, Pokholkova GV, Belyaeva ES, et al High-resolution analysis of Drosophila heterochromatin organization using SuUR Su(var)3-9 double mutants. Proc Natl Acad Sci U S A. 2007;104(31):12819–24. 10.1073/pnas.0704690104 17640911 PMC1937550

[24] Lohe AR, Hilliker AJ, Roberts PA. Mapping simple repeated DNA sequences in heterochromatin of *Drosophila melanogaster*. Genetics. 1993;134(4):1149–74. Epub 1993/08/01. 8375654 10.1093/genetics/134.4.1149 PMC1205583

[25] Jagannathan M, Warsinger-Pepe N, Watase GJ, Yamashita YM. Comparative Analysis of Satellite DNA in the *Drosophila melanogaster* Species Complex. G3 (Bethesda). 2017;7(2):693–704. Epub 2016/12/23. 10.1534/g3.116.035352 28007840 PMC5295612

[26] Tolchkov EV, Rasheva VI, Bonaccorsi S, Westphal T, Gvozdev VA. The size and internal structure of a heterochromatic block determine its ability to induce position effect variegation in *Drosophila melanogaster*. Genetics. 2000;154(4):1611–26. 10747057 10.1093/genetics/154.4.1611 PMC1461014

[27] Abad JP, Carmena M, Baars S, Saunders RD, Glover DM, Ludena P, et al Dodeca satellite: a conserved G+C-rich satellite from the centromeric heterochromatin of *Drosophila melanogaster*. Proc Natl Acad Sci U S A. 1992;89(10):4663–7. Epub 1992/05/15. 1584802 10.1073/pnas.89.10.4663 PMC49143

[28] Torok T, Harvie PD, Buratovich M, Bryant PJ. The product of proliferation disrupter is concentrated at centromeres and required for mitotic chromosome condensation and cell proliferation in Drosophila. Genes & Development. 1997;11(2):213–25. 10.1101/gad.11.2.213 9009204

[29] Torok T, Gorjanacz M, Bryant PJ, Kiss I. Prod is a novel DNA-binding protein that binds to the 1.686 g/cm(3) 10 bp satellite repeat of *Drosophila melanogaster*. Nucleic Acids Res. 2000;28(18):3551–7. 10982875 10.1093/nar/28.18.3551 PMC110743

[30] Blower MD, Karpen GH. The role of Drosophila CID in kinetochore formation, cell-cycle progression and heterochromatin interactions. Nat Cell Biol. 2001;3(8):730–9. 10.1038/35087045 11483958 PMC3229202

[31] Wei KH, Lower SE, Caldas IV, Sless TJS, Barbash DA, Clark AG. Variable Rates of Simple Satellite Gains across the Drosophila Phylogeny. Mol Biol Evol. 2018;35(4):925–41. 10.1093/molbev/msy005 29361128 PMC5888958

[32] Chaisson MJ, Huddleston J, Dennis MY, Sudmant PH, Malig M, Hormozdiari F, et al Resolving the complexity of the human genome using single-molecule sequencing. Nature. 2014 10.1038/nature13907 . 25383537 PMC4317254

[33] Jain D, Baldi S, Zabel A, Straub T, Becker PB. Active promoters give rise to false positive 'Phantom Peaks' in ChIP-seq experiments. Nucleic Acids Res. 2015;43(14):6959–68. 10.1093/nar/gkv637 26117547 PMC4538825

[34] Erhardt S, Mellone BG, Betts CM, Zhang W, Karpen GH, Straight AF. Genome-wide analysis reveals a cell cycle-dependent mechanism controlling centromere propagation. J Cell Biol. 2008;183(5):805–18. 10.1083/jcb.200806038 19047461 PMC2592830

[35] Lee H, McManus CJ, Cho DY, Eaton M, Renda F, Somma MP, et al DNA copy number evolution in Drosophila cell lines. Genome Biol. 2014;15(8):R70 10.1186/gb-2014-15-8-r70 25262759 PMC4289277

[36] Kofler R, Betancourt AJ, Schlotterer C. Sequencing of pooled DNA samples (Pool-Seq) uncovers complex dynamics of transposable element insertions in *Drosophila melanogaster*. PLoS Genet. 2012;8(1):e1002487 10.1371/journal.pgen.1002487 22291611 PMC3266889

[37] Chang C-H, Chavan A, Palladino J, Wei X, Martins NMC, Santinello B, et al Data from: Islands of retroelements are major components of Drosophila centromeres. Dryad Digital Repository. 2019 10.5061/dryad.rb1bt3j. PMC6516634 31086362

[38] Beliveau BJ, Joyce EF, Apostolopoulos N, Yilmaz F, Fonseka CY, McCole RB, et al Versatile design and synthesis platform for visualizing genomes with Oligopaint FISH probes. Proc Natl Acad Sci U S A. 2012;109(52):21301–6. 10.1073/pnas.1213818110 23236188 PMC3535588

[39] Mellone B, Erhardt S, Karpen GH. The ABCs of centromeres. Nat Cell Biol. 2006;8(5):427–9. 10.1038/ncb0506-427 . 16691204

[40] Sexton T, Yaffe E, Kenigsberg E, Bantignies F, Leblanc B, Hoichman M, et al Three-dimensional folding and functional organization principles of the Drosophila genome. Cell. 2012;148(3):458–72. 10.1016/j.cell.2012.01.010 . 22265598

[41] Ogiyama Y, Schuettengruber B, Papadopoulos GL, Chang JM, Cavalli G. Polycomb-Dependent Chromatin Looping Contributes to Gene Silencing during Drosophila Development. Mol Cell. 2018;71(1):73–88 e5. 10.1016/j.molcel.2018.05.032 . 30008320

[42] Sullivan BA. Optical mapping of protein-DNA complexes on chromatin fibers. Methods Mol Biol. 2010;659:99–115. 10.1007/978-1-60761-789-1_7 . 20809306

[43] Khost DE, Eickbush DG, Larracuente AM. Single-molecule sequencing resolves the detailed structure of complex satellite DNA loci in *Drosophila melanogaster*. Genome research. 2017;27(5):709–21. 10.1101/gr.213512.116 28373483 PMC5411766

[44] Garrigan D, Kingan SB, Geneva AJ, Andolfatto P, Clark AG, Thornton KR, et al Genome sequencing reveals complex speciation in the *Drosophila simulans* clade. Genome research. 2012;22(8):1499–511. Epub 2012/04/27. 10.1101/gr.130922.111 22534282 PMC3409263

[45] Lohe AR, Brutlag DL. Identical satellite DNA sequences in sibling species of Drosophila. J Mol Biol. 1987;194(2):161–70. . 3112413 10.1016/0022-2836(87)90365-2

[46] Rosin L, Mellone BG. Co-evolving CENP-A and CAL1 Domains Mediate Centromeric CENP-A Deposition across Drosophila Species. Dev Cell. 2016;37(2):136–47. 10.1016/j.devcel.2016.03.021 27093083 PMC4861639

[47] Luo S, Mach J, Abramson B, Ramirez R, Schurr R, Barone P, et al The cotton centromere contains a Ty3-gypsy-like LTR retroelement. PLoS ONE. 2012;7(4):e35261 10.1371/journal.pone.0035261 22536361 PMC3334964

[48] Gent JI, Wang N, Dawe RK. Stable centromere positioning in diverse sequence contexts of complex and satellite centromeres of maize and wild relatives. Genome Biol. 2017;18(1):121 10.1186/s13059-017-1249-4 28637491 PMC5480163

[49] Nagaki K, Cheng Z, Ouyang S, Talbert PB, Kim M, Jones KM, et al Sequencing of a rice centromere uncovers active genes. Nat Genet. 2004;36(2):138–45. 10.1038/ng1289 . 14716315

[50] Nagaki K, Neumann P, Zhang D, Ouyang S, Buell CR, Cheng Z, et al Structure, divergence, and distribution of the CRR centromeric retrotransposon family in rice. Mol Biol Evol. 2005;22(4):845–55. 10.1093/molbev/msi069 . 15616142

[51] Cheng Z, Dong F, Langdon T, Ouyang S, Buell CR, Gu M, et al Functional rice centromeres are marked by a satellite repeat and a centromere-specific retrotransposon. Plant Cell. 2002;14(8):1691–704. 10.1105/tpc.003079 12172016 PMC151459

[52] Liu Z, Yue W, Li D, Wang RR, Kong X, Lu K, et al Structure and dynamics of retrotransposons at wheat centromeres and pericentromeres. Chromosoma. 2008;117(5):445–56. 10.1007/s00412-008-0161-9 . 18496705

[53] Yadav V, Sun S, Billmyre RB, Thimmappa BC, Shea T, Lintner R, et al RNAi is a critical determinant of centromere evolution in closely related fungi. Proc Natl Acad Sci U S A. 2018;115(12):3108–13. 10.1073/pnas.1713725115 29507212 PMC5866544

[54] de Sotero-Caio CG, Cabral-de-Mello DC, Calixto MDS, Valente GT, Martins C, Loreto V, et al Centromeric enrichment of LINE-1 retrotransposons and its significance for the chromosome evolution of Phyllostomid bats. Chromosome Res. 2017;25(3–4):313–25. 10.1007/s10577-017-9565-9 . 28916913

[55] Carbone L, Harris RA, Mootnick AR, Milosavljevic A, Martin DI, Rocchi M, et al Centromere remodeling in *Hoolock leuconedys* (Hylobatidae) by a new transposable element unique to the gibbons. Genome Biol Evol. 2012;4(7):648–58. 10.1093/gbe/evs048 22593550 PMC3606032

[56] Miga KH, Newton Y, Jain M, Altemose N, Willard HF, Kent WJ. Centromere reference models for human chromosomes X and Y satellite arrays. Genome research. 2014;24(4):697–707. 10.1101/gr.159624.113 24501022 PMC3975068

[57] Chueh AC, Wong LH, Wong N, Choo KH. Variable and hierarchical size distribution of L1-retroelement-enriched CENP-A clusters within a functional human neocentromere. Hum Mol Genet. 2005;14(1):85–93. 10.1093/hmg/ddi008 . 15537667

[58] Longo MS, Carone DM, Program NCS, Green ED, O'Neill MJ, O'Neill RJ. Distinct retroelement classes define evolutionary breakpoints demarcating sites of evolutionary novelty. BMC Genomics. 2009;10:334 10.1186/1471-2164-10-334 19630942 PMC2736999

[59] Ferreri GC, Brown JD, Obergfell C, Jue N, Finn CE, O'Neill MJ, et al Recent amplification of the kangaroo endogenous retrovirus, KERV, limited to the centromere. J Virol. 2011;85(10):4761–71. 10.1128/JVI.01604-10 21389136 PMC3126163

[60] Renfree MB, Papenfuss AT, Deakin JE, Lindsay J, Heider T, Belov K, et al Genome sequence of an Australian kangaroo, *Macropus eugenii*, provides insight into the evolution of mammalian reproduction and development. Genome Biol. 2011;12(8):R81 10.1186/gb-2011-12-8-r81 21854559 PMC3277949

[61] Johnson RN, O'Meally D, Chen Z, Etherington GJ, Ho SYW, Nash WJ, et al Adaptation and conservation insights from the koala genome. Nat Genet. 2018;50(8):1102–11. 10.1038/s41588-018-0153-5 29967444 PMC6197426

[62] Kumar S, Stecher G, Suleski M, Hedges SB. TimeTree: A Resource for Timelines, Timetrees, and Divergence Times. Mol Biol Evol. 2017;34(7):1812–9. 10.1093/molbev/msx116 . 28387841

[63] O'Neill RJ, O'Neill MJ, Graves JA. Undermethylation associated with retroelement activation and chromosome remodelling in an interspecific mammalian hybrid. Nature. 1998;393(6680):68–72. 10.1038/29985 . 9590690

[64] Schneider KL, Xie Z, Wolfgruber TK, Presting GG. Inbreeding drives maize centromere evolution. Proc Natl Acad Sci U S A. 2016;113(8):E987–96. 10.1073/pnas.1522008113 26858403 PMC4776452

[65] Nergadze SG, Piras FM, Gamba R, Corbo M, Cerutti F, McCarter JGW, et al Birth, evolution, and transmission of satellite-free mammalian centromeric domains. Genome research. 2018;28(6):789–99. 10.1101/gr.231159.117 . 29712753 PMC5991519

[66] Chueh AC, Northrop EL, Brettingham-Moore KH, Choo KH, Wong LH. LINE retrotransposon RNA is an essential structural and functional epigenetic component of a core neocentromeric chromatin. PLoS Genet. 2009;5(1):e1000354 10.1371/journal.pgen.1000354 19180186 PMC2625447

[67] Sharma A, Wolfgruber TK, Presting GG. Tandem repeats derived from centromeric retrotransposons. BMC Genomics. 2013;14:142 10.1186/1471-2164-14-142 23452340 PMC3648361

[68] Zhang H, Koblizkova A, Wang K, Gong Z, Oliveira L, Torres GA, et al Boom-Bust Turnovers of Megabase-Sized Centromeric DNA in Solanum Species: Rapid Evolution of DNA Sequences Associated with Centromeres. Plant Cell. 2014;26(4):1436–47. 10.1105/tpc.114.123877 24728646 PMC4036563

[69] Presting GG. Centromeric retrotransposons and centromere function. Curr Opin Genet Dev. 2018;49:79–84. 10.1016/j.gde.2018.03.004 . 29597064

[70] Choi ES, Stralfors A, Catania S, Castillo AG, Svensson JP, Pidoux AL, et al Factors that promote H3 chromatin integrity during transcription prevent promiscuous deposition of CENP-A(Cnp1) in fission yeast. PLoS Genet. 2012;8(9):e1002985 10.1371/journal.pgen.1002985 23028377 PMC3447972

[71] Choi ES, Strålfors A, Castillo AG, Durand-Dubief M, Ekwall K, Allshire RC. Identification of Noncoding Transcripts from within CENP-A Chromatin at Fission Yeast Centromeres. Journal of Biological Chemistry. 2011;286(26):23600–7. 10.1074/jbc.M111.228510 21531710 PMC3123123

[72] Carone DM, Zhang C, Hall LE, Obergfell C, Carone BR, O'Neill MJ, et al Hypermorphic expression of centromeric retroelement-encoded small RNAs impairs CENP-A loading. Chromosome Res. 2013;21(1):49–62. 10.1007/s10577-013-9337-0 . 23392618

[73] Chan FL, Marshall OJ, Saffery R, Kim BW, Earle E, Choo KH, et al Active transcription and essential role of RNA polymerase II at the centromere during mitosis. Proc Natl Acad Sci U S A. 2012;109(6):1979–84. 10.1073/pnas.1108705109 22308327 PMC3277563

[74] Bobkov GOM, Gilbert N, Heun P. Centromere transcription allows CENP-A to transit from chromatin association to stable incorporation. J Cell Biol. 2018;217(6):1957–72. 10.1083/jcb.201611087 29626011 PMC5987708

[75] Rosic S, Kohler F, Erhardt S. Repetitive centromeric satellite RNA is essential for kinetochore formation and cell division. J Cell Biol. 2014 10.1083/jcb.201404097 . 25365994 PMC4226727

[76] Stupar RM, Song J, Tek AL, Cheng Z, Dong F, Jiang J. Highly condensed potato pericentromeric heterochromatin contains rDNA-related tandem repeats. Genetics. 2002;162(3):1435–44. 12454086 10.1093/genetics/162.3.1435 PMC1462313

[77] Lim KY, Skalicka K, Koukalova B, Volkov RA, Matyasek R, Hemleben V, et al Dynamic changes in the distribution of a satellite homologous to intergenic 26-18S rDNA spacer in the evolution of Nicotiana. Genetics. 2004;166(4):1935–46. 15126410 10.1534/genetics.166.4.1935 PMC1470824

[78] Jo SH, Koo DH, Kim JF, Hur CG, Lee S, Yang TJ, et al Evolution of ribosomal DNA-derived satellite repeat in tomato genome. BMC Plant Biol. 2009;9:42 10.1186/1471-2229-9-42 19351415 PMC2679016

[79] Falquet J, Creusot F, Dron M. Molecular analysis of *Phaseolus vulgaris* rDNA unit and characterization of a satellite DNA homologous to IGS subrepeats. Plant Physiology and Biochemistry. 1997;35(8):611–22. WOS:A1997XQ73100005.

[80] Catania S, Pidoux AL, Allshire RC. Sequence features and transcriptional stalling within centromere DNA promote establishment of CENP-A chromatin. PLoS Genet. 2015;11(3):e1004986 10.1371/journal.pgen.1004986 25738810 PMC4349457

[81] Garavis M, Escaja N, Gabelica V, Villasante A, Gonzalez C. Centromeric Alpha-Satellite DNA Adopts Dimeric i-Motif Structures Capped by AT Hoogsteen Base Pairs. Chemistry. 2015;21(27):9816–24. 10.1002/chem.201500448 . 26013031

[82] Chen CC, Bowers S, Lipinszki Z, Palladino J, Trusiak S, Bettini E, et al Establishment of Centromeric Chromatin by the CENP-A Assembly Factor CAL1 Requires FACT-Mediated Transcription. Dev Cell. 2015;34(1):73–84. 10.1016/j.devcel.2015.05.012 26151904 PMC4495351

[83] Sandmann T, Jakobsen JS, Furlong EE. ChIP-on-chip protocol for genome-wide analysis of transcription factor binding in *Drosophila melanogaster* embryos. Nat Protoc. 2006;1(6):2839–55. 10.1038/nprot.2006.383 . 17406543

[84] Bonn S, Zinzen RP, Perez-Gonzalez A, Riddell A, Gavin AC, Furlong EE. Cell type-specific chromatin immunoprecipitation from multicellular complex samples using BiTS-ChIP. Nat Protoc. 2012;7(5):978–94. 10.1038/nprot.2012.049 . 22538849

[85] Bonn S, Zinzen RP, Girardot C, Gustafson EH, Perez-Gonzalez A, Delhomme N, et al Tissue-specific analysis of chromatin state identifies temporal signatures of enhancer activity during embryonic development. Nat Genet. 2012;44(2):148–56. 10.1038/ng.1064 . 22231485

[86] Landt SG, Marinov GK, Kundaje A, Kheradpour P, Pauli F, Batzoglou S, et al ChIP-seq guidelines and practices of the ENCODE and modENCODE consortia. Genome research. 2012;22(9):1813–31. 10.1101/gr.136184.111 22955991 PMC3431496

[87] Kharchenko PV, Tolstorukov MY, Park PJ. Design and analysis of ChIP-seq experiments for DNA-binding proteins. Nat Biotechnol. 2008;26(12):1351–9. 10.1038/nbt.1508 19029915 PMC2597701

[88] Bao W, Kojima KK, Kohany O. Repbase Update, a database of repetitive elements in eukaryotic genomes. Mob DNA. 2015;6:11 Epub 2015/06/06. 10.1186/s13100-015-0041-9 26045719 PMC4455052

[89] Nurk S, Bankevich A, Antipov D, Gurevich A, Korobeynikov A, Lapidus A, et al, editors. Assembling Genomes and Mini-metagenomes from Highly Chimeric Reads. Berlin, Heidelberg: Springer Berlin Heidelberg; 2013.

[90] Zhang Y, Liu T, Meyer CA, Eeckhoute J, Johnson DS, Bernstein BE, et al Model-based analysis of ChIP-Seq (MACS). Genome Biol. 2008;9(9):R137 Epub 2008/09/19. 10.1186/gb-2008-9-9-r137 18798982 PMC2592715

[91] Altschul SF, Gish W, Miller W, Myers EW, Lipman DJ. Basic local alignment search tool. J Mol Biol. 1990;215(3):403–10. Epub 1990/10/05. 10.1016/S0022-2836(05)80360-2 . 2231712

[92] Kim K, Peluso P, Babayan P, Yeadon PJ, Yu C, Fisher WW, et al Long-read, whole-genome shotgun sequence data for five model organisms. Scientific data. 2014;1(140045). Epub 11/25/2014. 10.1038/sdata.2014.45 25977796 PMC4365909

[93] Solares EA, Chakraborty M, Miller DE, Kalsow S, Hall K, Perera AG, et al Rapid Low-Cost Assembly of the *Drosophila melanogaster* Reference Genome Using Low-Coverage, Long-Read Sequencing. G3 (Bethesda). 2018;8(10):3143–54. 10.1534/g3.118.200162 30018084 PMC6169397

[94] Koren S, Walenz BP, Berlin K, Miller JR, Bergman NH, Phillippy AM. Canu: scalable and accurate long-read assembly via adaptive k-mer weighting and repeat separation. Genome research. 2017;27(5):722–36. Epub 2017/03/17. 10.1101/gr.215087.116 28298431 PMC5411767

[95] Smit A, Hubley R, Green P. RepeatMasker. 2013 Available from: http://www.repeatmasker.org.

[96] Benson G. Tandem repeats finder: a program to analyze DNA sequences. Nucleic Acids Res. 1999;27(2):573–80. Epub 1998/12/24. 9862982 10.1093/nar/27.2.573 PMC148217

[97] Walker BJ, Abeel T, Shea T, Priest M, Abouelliel A, Sakthikumar S, et al Pilon: an integrated tool for comprehensive microbial variant detection and genome assembly improvement. PLoS ONE. 2014;9(11):e112963 10.1371/journal.pone.0112963 25409509 PMC4237348

[98] Gutzwiller F, Carmo CR, Miller DE, Rice DW, Newton IL, Hawley RS, et al Dynamics of Wolbachia pipientis Gene Expression Across the *Drosophila melanogaster* Life Cycle. G3 (Bethesda). 2015;5(12):2843–56. Epub 2015/10/27. 10.1534/g3.115.021931 26497146 PMC4683655

[99] Yarosh W, Spradling AC. Incomplete replication generates somatic DNA alterations within Drosophila polytene salivary gland cells. Genes Dev. 2014;28(16):1840–55. Epub 2014/08/17. 10.1101/gad.245811.114 25128500 PMC4197960

[100] McCoy RC, Taylor RW, Blauwkamp TA, Kelley JL, Kertesz M, Pushkarev D, et al Illumina TruSeq synthetic long-reads empower de novo assembly and resolve complex, highly-repetitive transposable elements. PLoS ONE. 2014;9(9):e106689 Epub 2014/09/05. 10.1371/journal.pone.0106689 25188499 PMC4154752

[101] Kent WJ. BLAT—the BLAST-like alignment tool. Genome research. 2002;12(4):656–64. Epub 2002/04/05. 10.1101/gr.229202 Article published online before March 2002. 11932250 PMC187518

[102] Zhao H, Sun Z, Wang J, Huang H, Kocher JP, Wang L. CrossMap: a versatile tool for coordinate conversion between genome assemblies. Bioinformatics. 2014;30(7):1006–7. Epub 2013/12/20. 10.1093/bioinformatics/btt730 24351709 PMC3967108

[103] Li H, Durbin R. Fast and accurate long-read alignment with Burrows-Wheeler transform. Bioinformatics. 2010;26(5):589–95. Epub 2010/01/19. 10.1093/bioinformatics/btp698 20080505 PMC2828108

[104] Li Q, Brown JB, Huang H, Bickel PJ. Measuring reproducibility of high-throughput experiments. Ann Appl Stat. 2011;5(3):1752–79. 10.1214/11-AOAS466

[105] Schmittgen TD, Livak KJ. Analyzing real-time PCR data by the comparative CT method. Nature Protocols. 2008;3(6):1101–8. 10.1038/nprot.2008.73 18546601

[106] Pimpinelli S, Bonaccorsi S, Fanti L, Gatti M. Immunostaining of mitotic chromosomes from Drosophila larval brain. Cold Spring Harb Protoc. 2011;2011(9). 10.1101/pdb.prot065524 . 21880821

[107] Beliveau BJ, Apostolopoulos N, Wu CT. Visualizing genomes with Oligopaint FISH probes. Curr Protoc Mol Biol. 2014;105:Unit 14 23. Epub 2014/02/11. 10.1002/0471142727.mb1423s105 24510436 PMC3928790

[108] Dimitri P. Fluorescent in situ hybridization with transposable element probes to mitotic chromosomal heterochromatin of Drosophila. Methods Mol Biol. 2004;260:29–39. 10.1385/1-59259-755-6:029 . 15020800

[109] Beliveau BJ, Kishi JY, Nir G, Sasaki HM, Saka SK, Nguyen SC, et al OligoMiner provides a rapid, flexible environment for the design of genome-scale oligonucleotide in situ hybridization probes. Proc Natl Acad Sci U S A. 2018;115(10):E2183–E92. 10.1073/pnas.1714530115 29463736 PMC5877937

[110] Langmead B, Salzberg SL. Fast gapped-read alignment with Bowtie 2. Nat Methods. 2012;9(4):357–9. Epub 2012/03/06. 10.1038/nmeth.1923 22388286 PMC3322381

[111] Marcais G, Kingsford C. A fast, lock-free approach for efficient parallel counting of occurrences of k-mers. Bioinformatics. 2011;27(6):764–70. 10.1093/bioinformatics/btr011 21217122 PMC3051319

[112] Dirks RM, Pierce NA. A partition function algorithm for nucleic acid secondary structure including pseudoknots. J Comput Chem. 2003;24(13):1664–77. 10.1002/jcc.10296 . 12926009

[113] Servant N, Varoquaux N, Lajoie BR, Viara E, Chen CJ, Vert JP, et al HiC-Pro: an optimized and flexible pipeline for Hi-C data processing. Genome Biol. 2015;16:259 10.1186/s13059-015-0831-x 26619908 PMC4665391

[114] Quinlan AR, Hall IM. BEDTools: a flexible suite of utilities for comparing genomic features. Bioinformatics. 2010;26(6):841–2. 10.1093/bioinformatics/btq033 20110278 PMC2832824

[115] Riddle NC, Minoda A, Kharchenko PV, Alekseyenko AA, Schwartz YB, Tolstorukov MY, et al Plasticity in patterns of histone modifications and chromosomal proteins in Drosophila heterochromatin. Genome research. 2011;21(2):147–63. 10.1101/gr.110098.110 21177972 PMC3032919

[116] Riddle NC, Jung YL, Gu T, Alekseyenko AA, Asker D, Gui H, et al Enrichment of HP1a on Drosophila chromosome 4 genes creates an alternate chromatin structure critical for regulation in this heterochromatic domain. PLoS Genet. 2012;8(9):e1002954 10.1371/journal.pgen.1002954 23028361 PMC3447959

[117] Benjamini Y, Hochberg Y. Controlling the False Discovery Rate—a Practical and Powerful Approach to Multiple Testing. J Roy Stat Soc B Met. 1995;57(1):289–300. WOS:A1995QE45300017.

[118] Kearse M, Moir R, Wilson A, Stones-Havas S, Cheung M, Sturrock S, et al Geneious Basic: an integrated and extendable desktop software platform for the organization and analysis of sequence data. Bioinformatics. 2012;28(12):1647–9. Epub 2012/05/01. 10.1093/bioinformatics/bts199 22543367 PMC3371832

[119] Stamatakis A. RAxML version 8: a tool for phylogenetic analysis and post-analysis of large phylogenies. Bioinformatics. 2014;30(9):1312–3. Epub 2014/01/24. 10.1093/bioinformatics/btu033 24451623 PMC3998144

[120] Paradis E, Claude J, Strimmer K. APE: Analyses of Phylogenetics and Evolution in R language. Bioinformatics. 2004;20(2):289–90. Epub 2004/01/22. . 14734327 10.1093/bioinformatics/btg412

[121] Laktionov PP, Maksimov DA, Romanov SE, Antoshina PA, Posukh OV, White-Cooper H, et al Genome-wide analysis of gene regulation mechanisms during Drosophila spermatogenesis. Epigenetics Chromatin. 2018;11(1):14 10.1186/s13072-018-0183-3 29609617 PMC5879934

[122] Gerstein MB, Rozowsky J, Yan KK, Wang D, Cheng C, Brown JB, et al Comparative analysis of the transcriptome across distant species. Nature. 2014;512(7515):445–8. 10.1038/nature13424 25164755 PMC4155737

[123] Kim D, Langmead B, Salzberg SL. HISAT: a fast spliced aligner with low memory requirements. Nat Methods. 2015;12(4):357–60. Epub 2015/03/10. 10.1038/nmeth.3317 25751142 PMC4655817

[124] Li H, Handsaker B, Wysoker A, Fennell T, Ruan J, Homer N, et al The Sequence Alignment/Map format and SAMtools. Bioinformatics. 2009;25(16):2078–9. 10.1093/bioinformatics/btp352 19505943 PMC2723002

[125] Wei KHC, Grenier JK, Barbash DA, Clark AG. Correlated variation and population differentiation in satellite DNA abundance among lines of *Drosophila melanogaster*. P Natl Acad Sci USA. 2014;111(52):18793–8. 10.1073/pnas.1421951112 WOS:000347444400085. 25512552 PMC4284603

