# Supplementary figures and images for: Islands of retroelements are major components of Drosophila centromeres
Source: PLoS Biol. 2019 May 14;17(5):e3000241. doi: 10.1371/journal.pbio.3000241 (PMC6516634; doi:10.1371/journal.pbio.3000241)

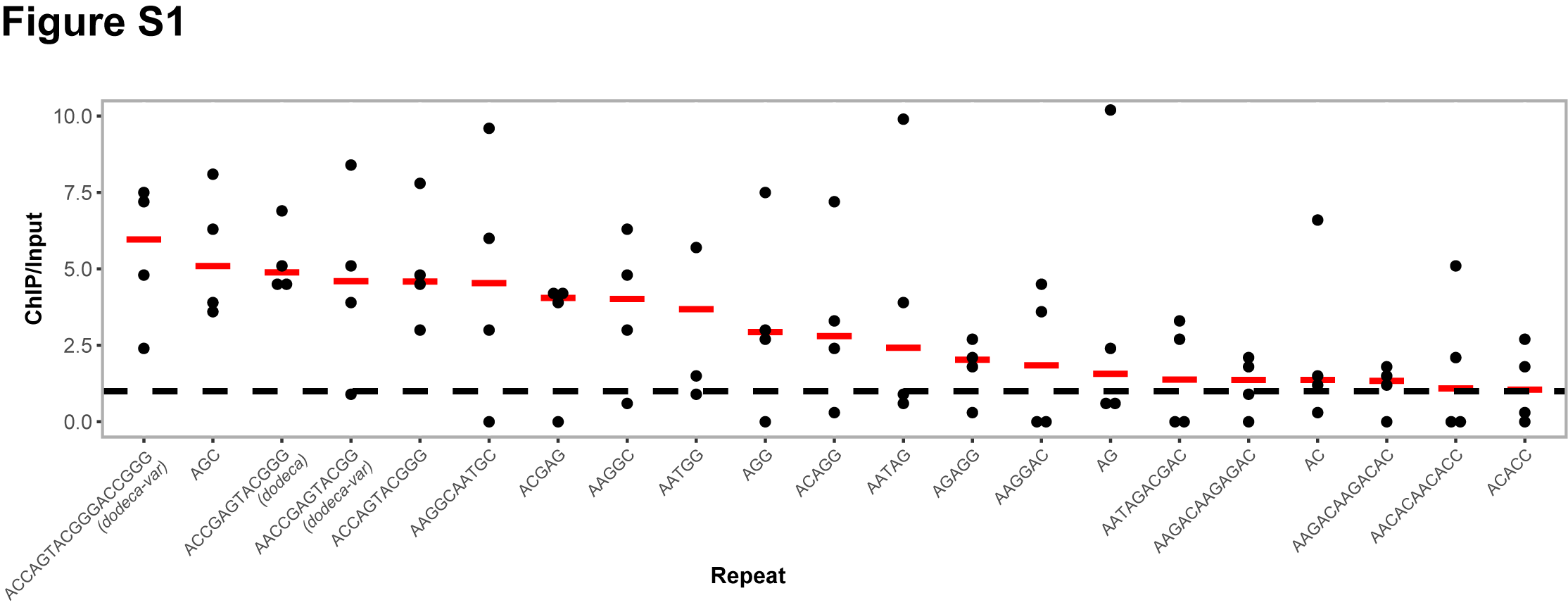

Supplement: S1 Fig — Plot of normalized CENP-A/input for simple tandem repeats for each ChIP-seq replicate, sorted by median (red lines). Shown are only the simple tandem repeats with median CENP-A/input > 1 in all four CENP-A ChIP replicates (see details in S1 Table). The simple tandem repeats with fewer than 10 counts of input reads in any one replicate are not shown. CENP-A, centromere protein A; ChIP, chromatin immunoprecipitation; ChIP-seq, ChIP sequencing. (TIF) [file pbio.3000241.s001.tif]

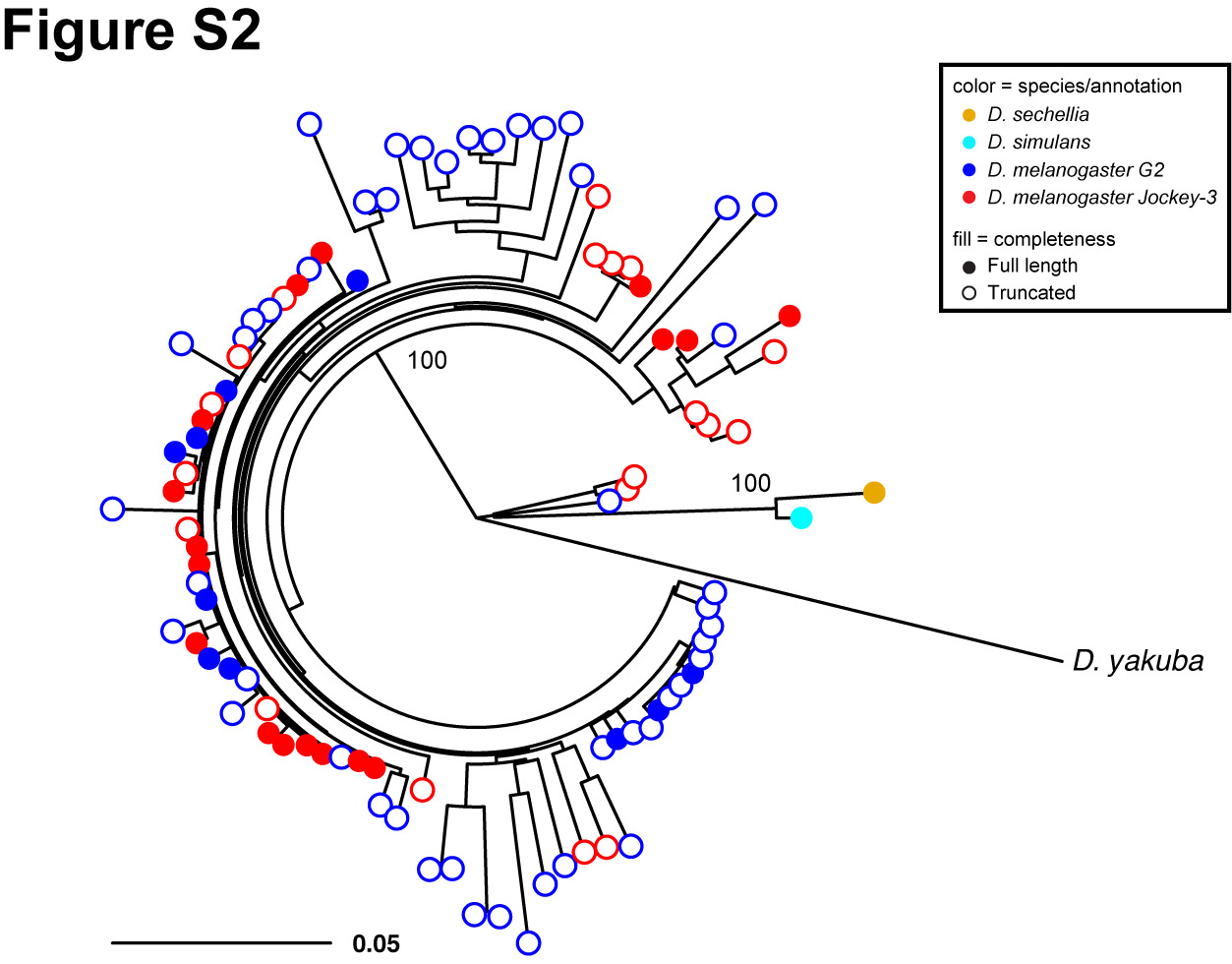

Supplement: S2 Fig — A maximum-likelihood phylogenetic tree showing the relationship between G2 and Jockey-3 sequences in D. melanogaster genome and closely related species in the simulans clade (D. simulans and D. sechellia) and D. yakuba. In D. melanogaster, G2 and Jockey-3 are interleaved across the phylogeny and thus likely correspond to the same repeat type. We therefore refer to these elements collectively as G2/Jockey-3 throughout the manuscript. (See Dryad repository files 13 and 15: https://doi.org/10.5061/dryad.rb1bt3j [37]). LTR, long terminal repeat. (TIF) [file pbio.3000241.s002.tif]

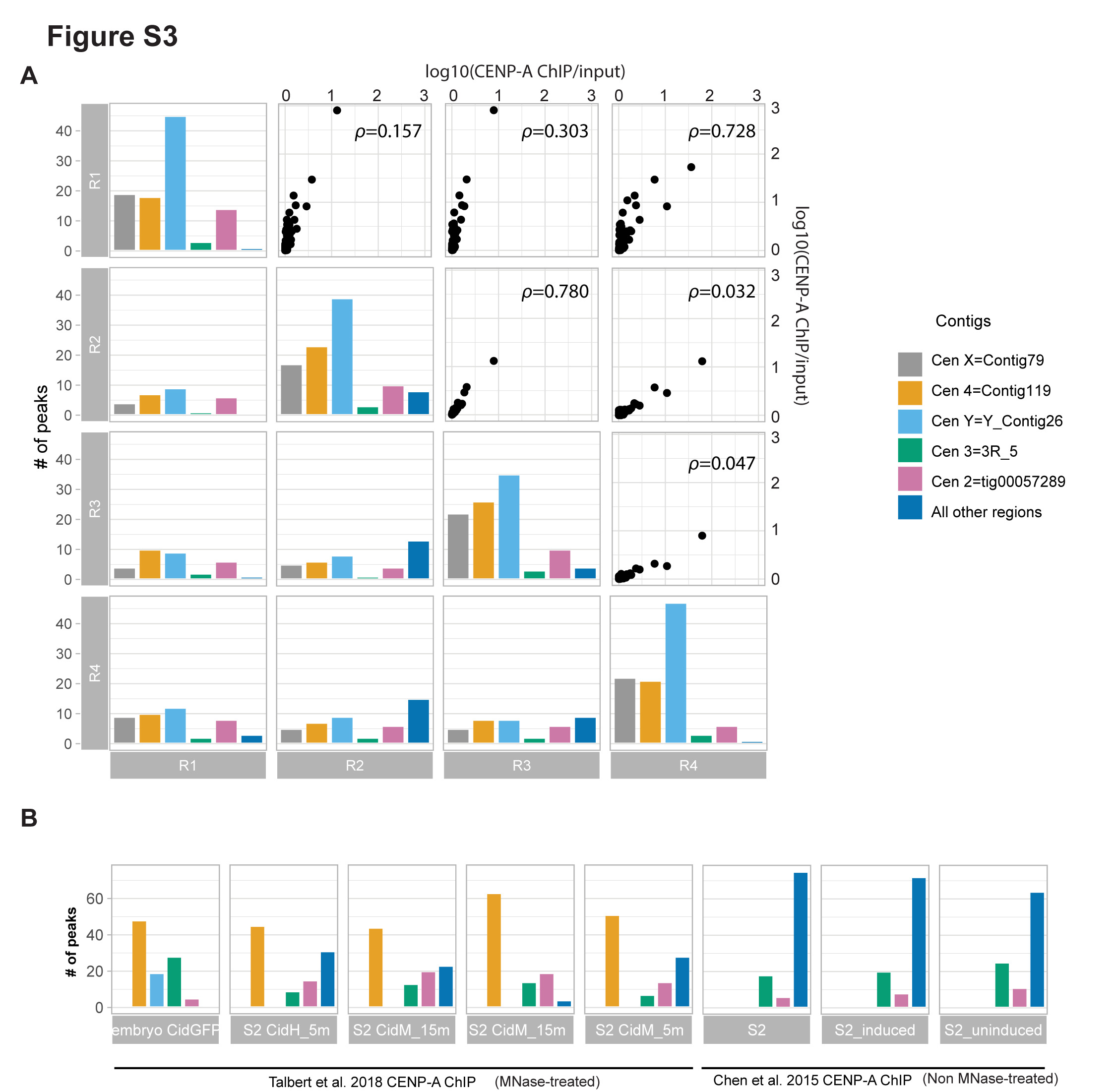

Supplement: S3 Fig — Locations of the top 100 strongest peaks for each ChIP experiment. (A) Plot of the location of top 100 strongest peaks for each ChIP experiment on the diagonal (see details in S4 Table). For the four replicate ChIP experiment in our OreR embryos, we examined the reproducibility of our experiments by first applying the IDR test and only keeping peaks with IDR ≤ 0.05. The number of these peaks is plotted below the diagonal. Between replicates 2 and 3, we found a total of 16,870 overlapping peaks, but 16,833 were weakly enriched relative to the overlapping peaks between other datasets because they are technical repeats with a shared library bias (Accel, see Materials and methods). We therefore only report the 37 strongest peaks (the average peak number of other comparisons between replicates). The IDR dataset comparisons are in S5 Table. We show the correlation between the CENP-A ChIP replicates above the diagonal. Plotted are the signal strength after IDR tests (normalized ChIP over input ratio from 1 to 1,000 on a log10 scale) with Spearman’s rho. The five contigs with the most consistent peaks within and among replicates correspond to the five centromeric candidates. (B) Plot of ChIP-seq data from S2 cells (this paper, [16, 82]) and an independent embryo CID–GFP (i.e., CENP-A–GFP) ChIP-seq dataset (see details in S4 Table; [16]; “5m” and “15m” represent different MNase treatments). The centromeric contigs are also CENP-A enriched in these independent datasets, with the exception of the X chromosome centromere contig. S2 cells lack a Y and are therefore not expected to have peaks on the Y candidate centromere contig. CENP-A, centromere protein A; ChIP, chromatin immunoprecipitation; ChIP-seq, ChIP sequencing; CID, centromere identifier; GFP, green fluorescent protein; IDR, irreproducible discovery rate; OreR, Oregon-R; S2, Schneider 2. (TIF) [file pbio.3000241.s003.tif]

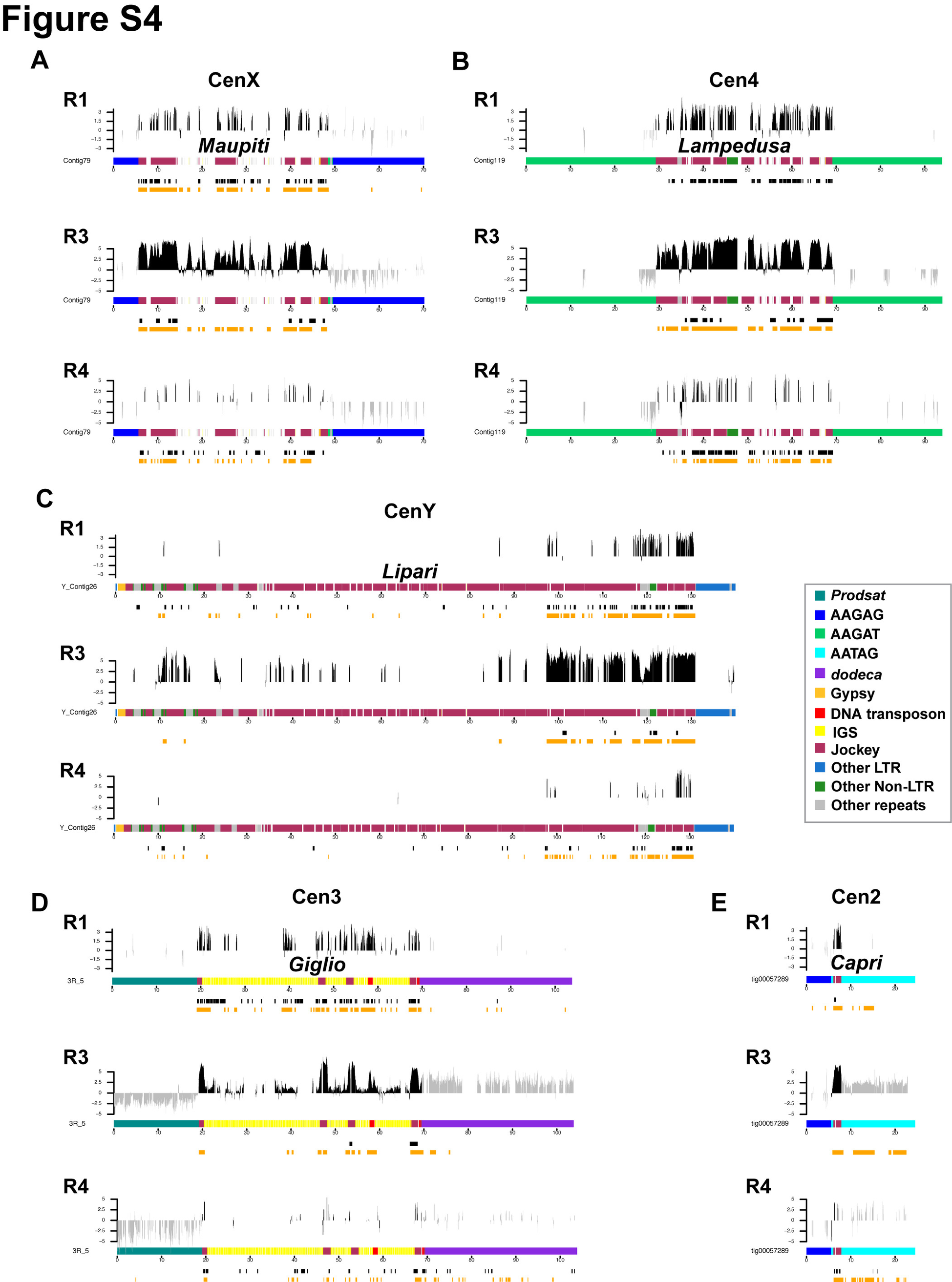

Supplement: S4 Fig — Organization of each CENP-A-enriched island corresponding to centromere candidates: (A) X centromere, (B) centromere 4; (C) Y centromere; (D) centromere 3; (E) centromere 2. Different repeat families are color coded (see legend; note that Jockey elements are shown in one color even though they are distinct elements). The normalized CENP-A enrichment over input (plotted on a log scale) is shown for three replicates (replicate 2 is in Fig 2) colored in gray for simple repeats and black for complex island sequences. Although the mapping quality scores are high in simple repeat regions, we do not use these data to make inferences about CENP-A distribution (see main text for details). The coordinates of the significantly CENP-A-enriched ChIPtigs mapped to these contigs (black) and the predicted ChIP peaks (orange) are shown below each plot. See Fig 2 and S3 and S4 Tables. CENP-A, centromere protein A; ChIP, chromatin immunoprecipitation. (TIF) [file pbio.3000241.s004.tif]

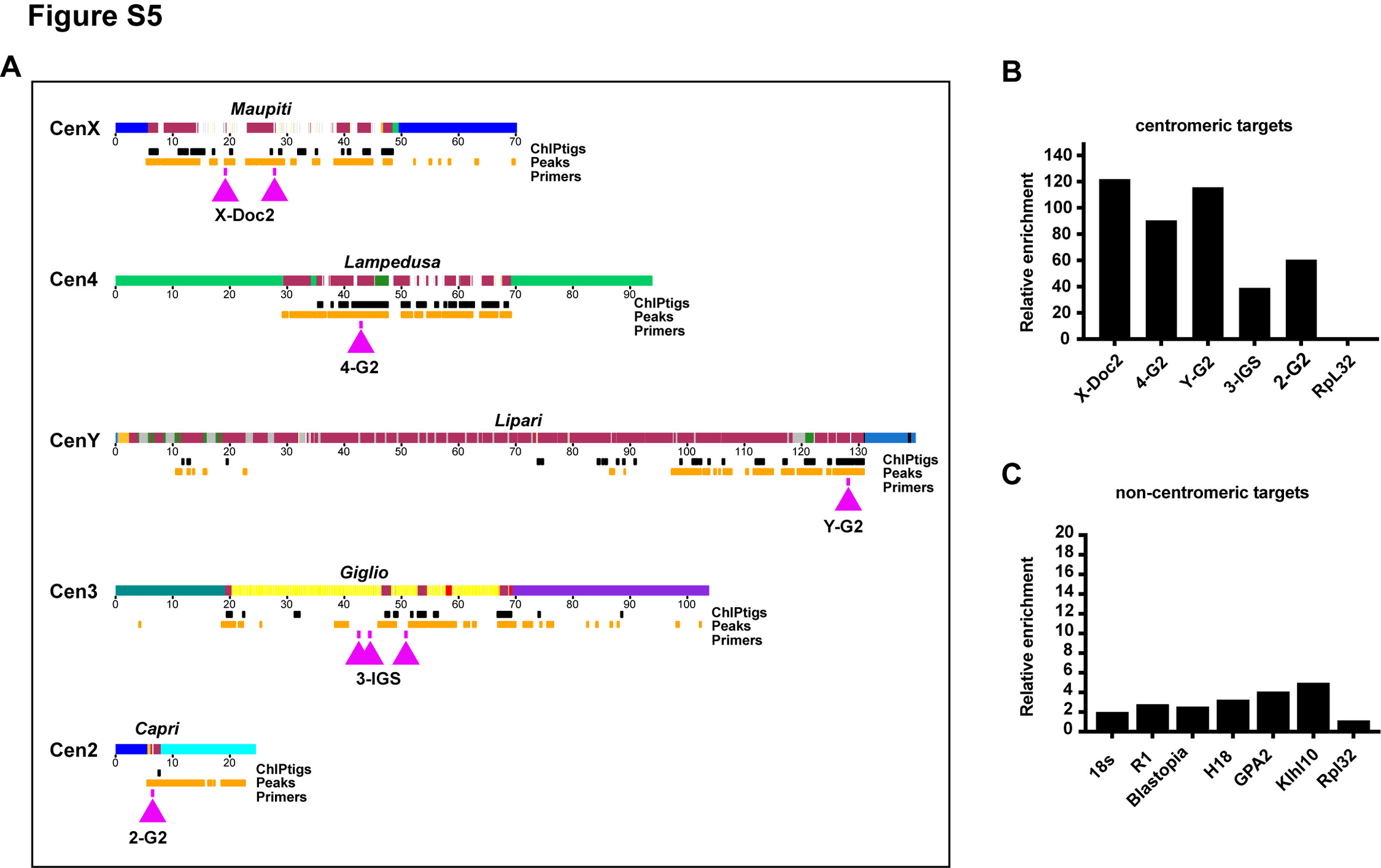

Supplement: S5 Fig — (A) Diagram showing putative centromere contigs showing the locations of CENP-A ChIPtigs in black and CENP-A MACS peaks in orange as in Fig 2. Locations of contig-specific qPCR primer binding sites are shown by magenta arrows. (B) Graph showing our ChIP-qPCR results using these primers. The enrichment is calculated relative to the input and is normalized by the RpL32 promoter region as a noncentromeric control. (C) Graph showing our ChIP-qPCR results using primers targeting other regions that showed CENP-A enrichment but that were not in our contigs. Again, the enrichment is calculated relative to the input and is normalized by RpL32 promoter as a noncentromeric control. We did not observe a robust CENP-A enrichment at these sites. The underlying data can be found in S2 Data. CENP-A, centromere protein A; ChIP, chromatin immunoprecipitation; qPCR, quantitative PCR. (TIF) [file pbio.3000241.s005.tif]

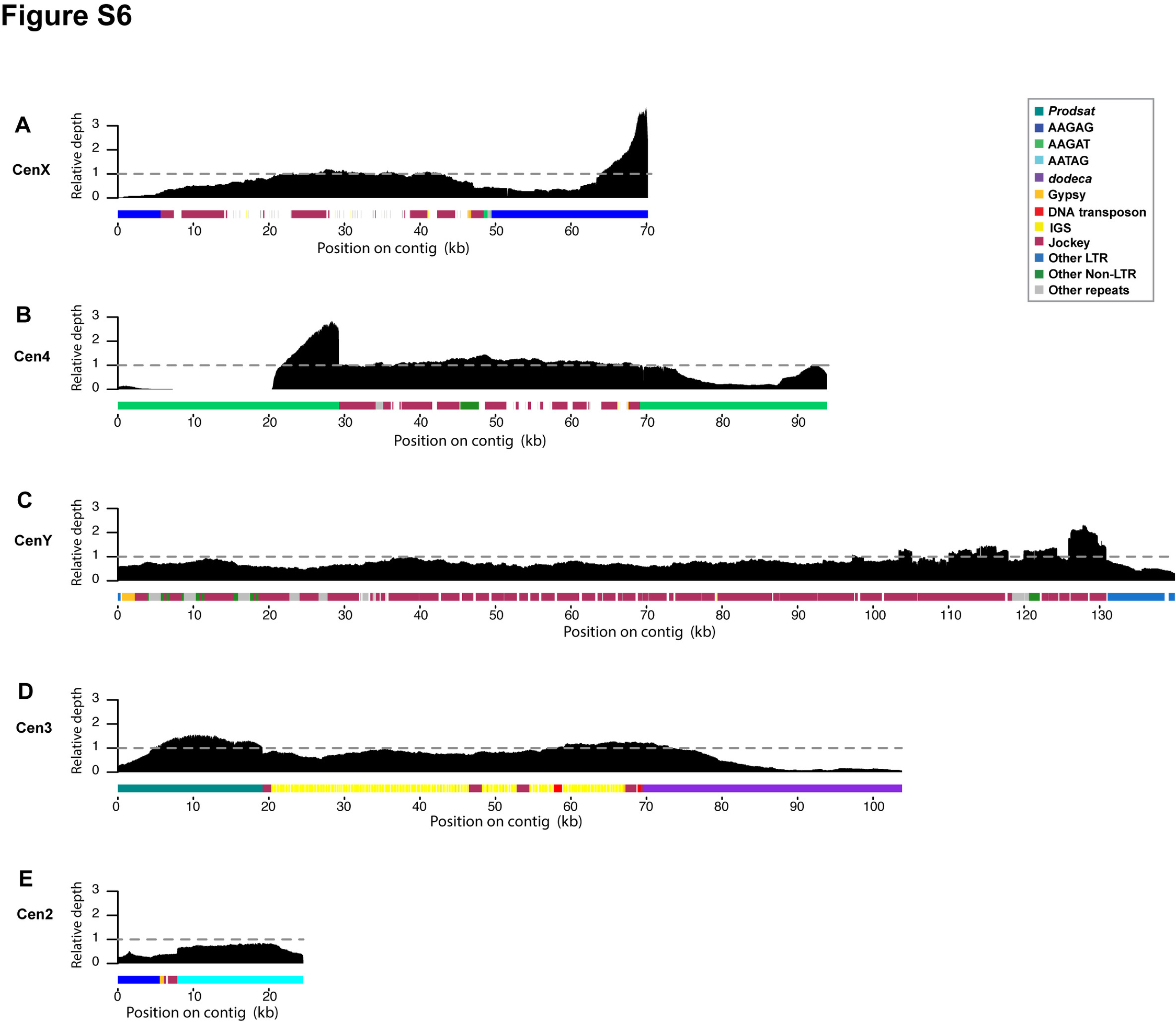

Supplement: S6 Fig — PacBio reads were mapped to the genome using Minimap (v 2.11) and the setting “-ax map-pb.” Shown are (A) X centromere, (B) centromere 4, (C) Y centromere, (D) centromere 3, and (E) centromere 2. The depth of only the high-quality mapped reads (mapped Q ≥ 30) was estimated for each position and normalized by the median depth of other genomic regions (98.32× for autosomes and 49.16× for sex chromosomes) to get relative depth. The relative depths of the TE-rich islands are close to 1, whereas the depth of the flanking simple satellites is uneven, with some regions > 1 and some < 1. We therefore exclude simple repeats from any assembly-based analyses and color these regions gray in Fig 2 and S4 Fig to indicate that caution should be used in interpreting these regions of the assembly. The underlying data can be found in S2 Data. TE, transposable element. (TIF) [file pbio.3000241.s006.tif]

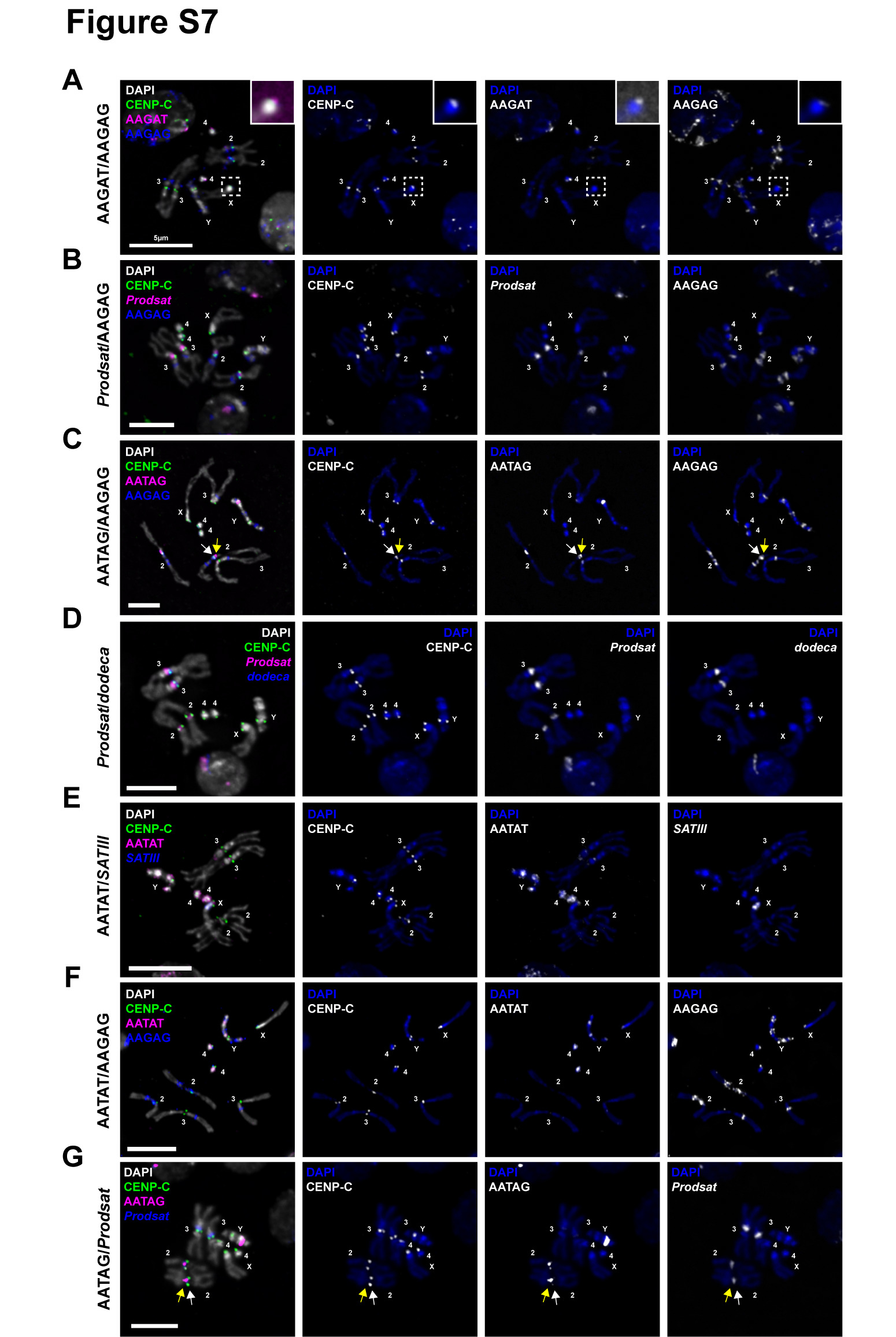

Supplement: S7 Fig — IF-FISH using an anti-CENP-C antibody (green) and satellite FISH probes in the following combinations: (A) AAGAT (magenta) and AAGAG (blue) with a high-contrast inset of AAGAT on the X chromosome; (B) Prodsat (magenta) and AAGAG (blue); (C) AATAG (magenta) and AAGAG (blue) with AATAG blocks identified by white (small block) and yellow (large block) arrows; (D) Prodsat (magenta) and dodeca (blue); (E) AATAT (magenta) and SATIII (blue); (F) AATAT (magenta) and AAGAG (blue); (G) AATAG (magenta) and Prodsat (blue) with AATAG blocks identified by white (small block) and yellow (large block) arrows. DAPI is shown in gray. The underlying data can be found in S2 Data. Bar 5 μm. CENP-C, centromere protein C; FISH, fluorescence in situ hybridization; IF, immunofluorescence; Prodsat, Prod satellite. (TIF) [file pbio.3000241.s007.tif]

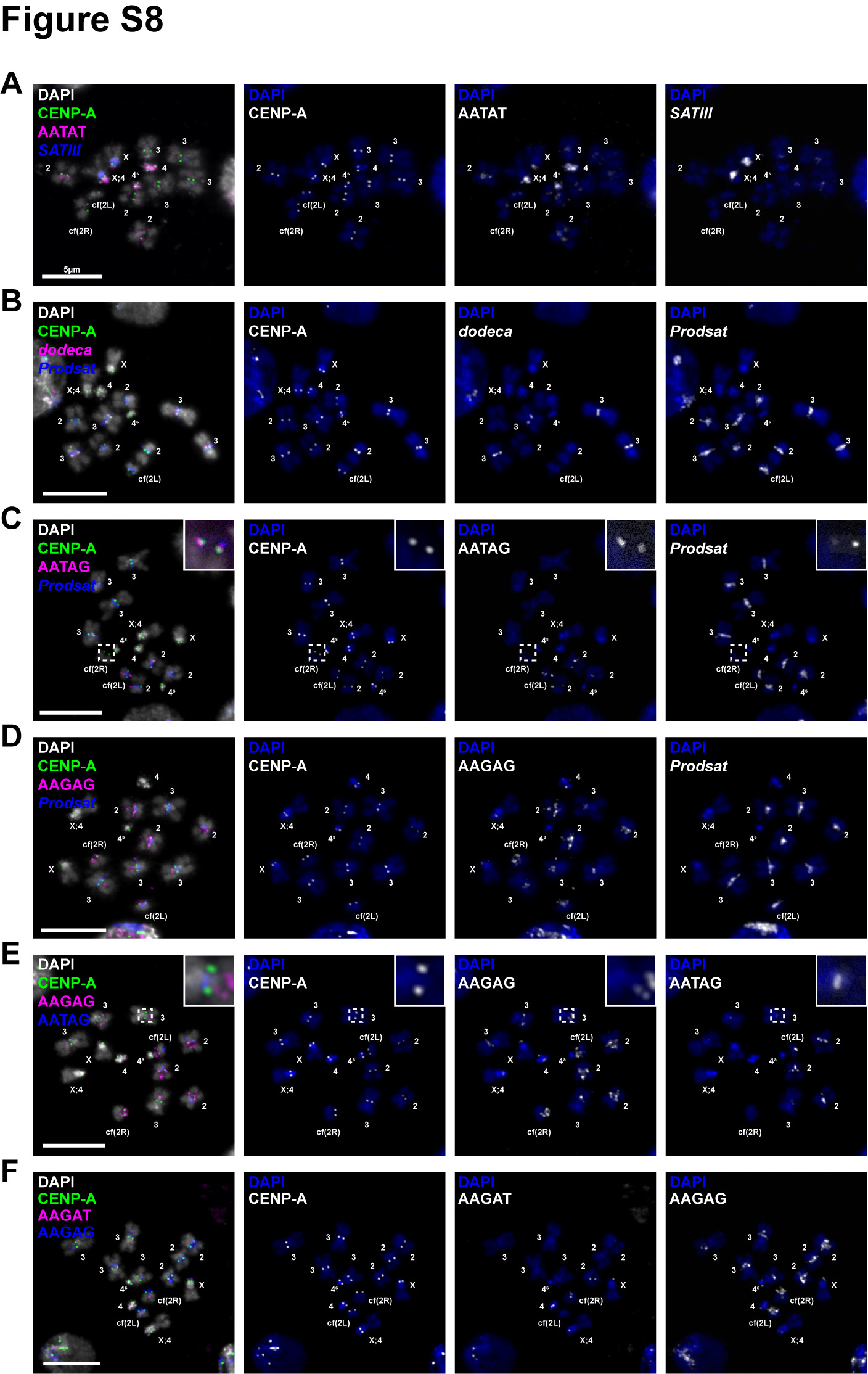

Supplement: S8 Fig — IF-FISH using an anti-CENP-A antibody (green) and satellite FISH probes in the following combinations: (A) AATAT (magenta) and SATIII (blue); (B) dodeca (magenta) and Prodsat (blue); (C) AATAG (magenta) and Prodsat (blue) with a high-contrast inset of AATAG and Prodsat on cf(2R); (D) AAGAG (magenta) and Prodsat (blue); (E) AAGAG (magenta) and AATAG (blue) with a high-contrast inset of AATAG on chromosome 3; (F) AAGAT (magenta) and AAGAG (blue). DAPI is shown in gray. See also S18 Table. Bar 5 μm. CENP-A, centromere protein A; cf(2R), centric fragment of chromosome 2R; FISH, fluorescence in situ hybridization; IF, immunofluorescence; Prodsat, Prod satellite; S2, Schneider 2. (TIF) [file pbio.3000241.s008.tif]

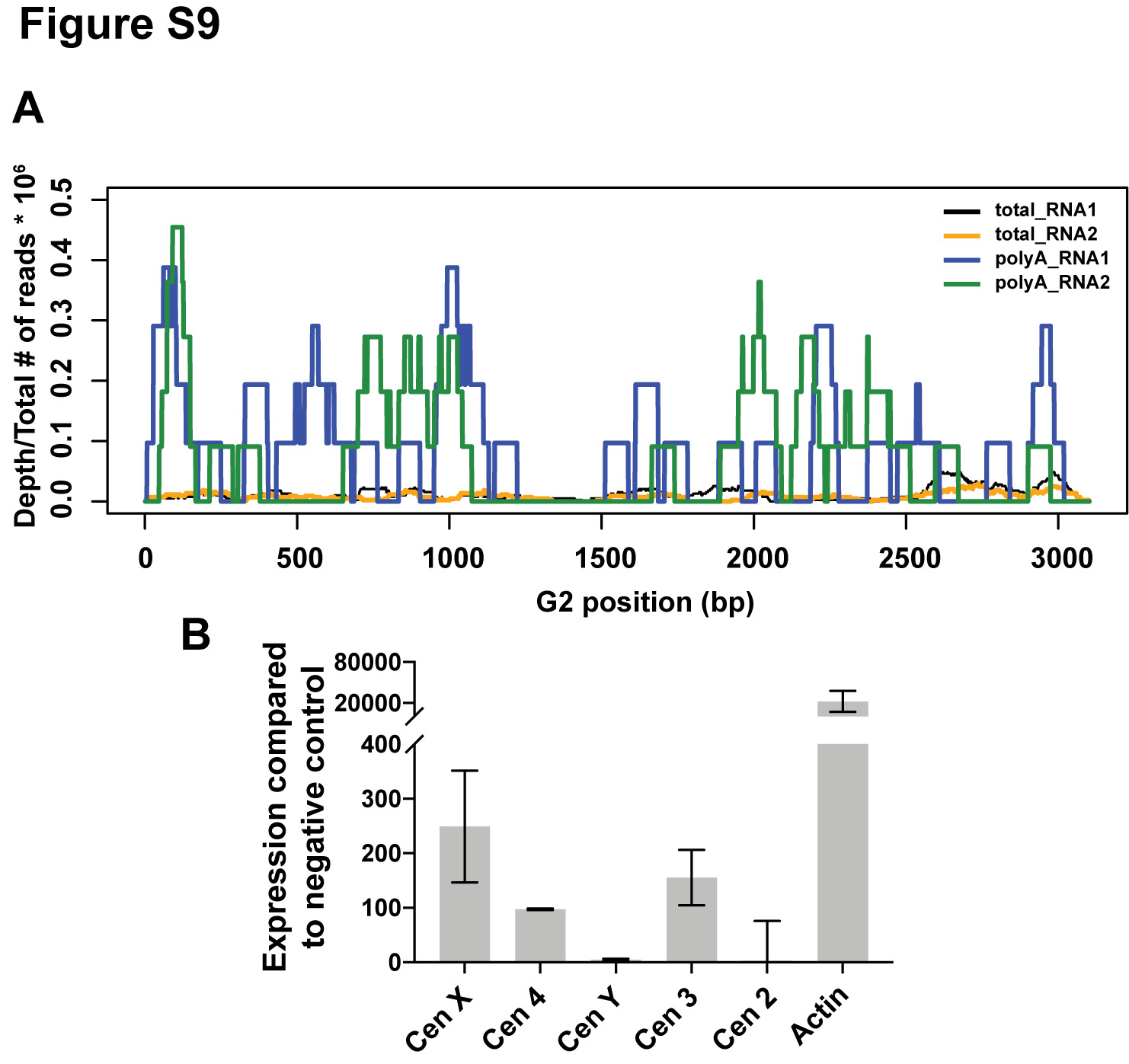

Supplement: S9 Fig — (A) Shown is the plot of the normalized reads depth from uniquely mapped reads (mapping quality ≥ 10) across the G2/Jockey-3 consensus element obtained from mapping total and poly-A RNA-seq data from testes [121, 122] to our repeat library. (B) Quantitative RT-PCR analysis of total RNA extracted from three independent overnight embryo collections. Expression levels were compared to the negative control gene Mst84Da (testis-specific). The G2/Jockey-3 copies surveyed on centromere (“Cen”) X, 4, and 3 but not Y and 2 show low levels of transcription compared to the housekeeping gene Actin. Although the primers (S7 Table) are specific for each centromere, the primer sets could amplify G2/Jockey-3 copies not included in our assembly. Error bars = SD. The underlying data for this figure can be found in S2 Data. Mst84Da, Male-specific RNA 84Da; RNA-seq, RNA sequencing; RT-PCR, reverse-transcription PCR. (TIF) [file pbio.3000241.s009.tif]

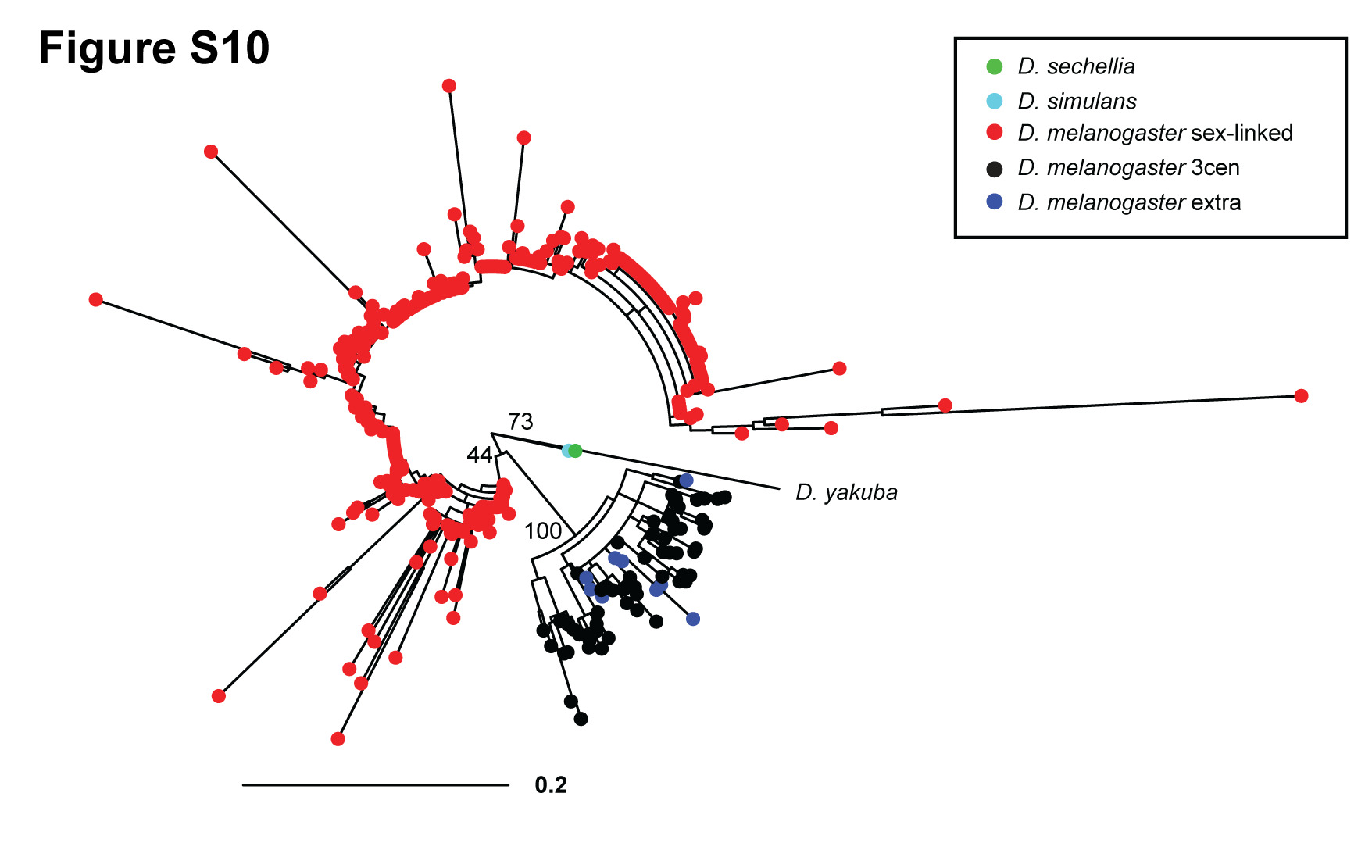

Supplement: S10 Fig — Maximum-likelihood phylogenetic tree of all individual IGS sequences found in the D. melanogaster genome with related outgroups (sequence alignment is in the Dryad repository file 12: https://doi.org/10.5061/dryad.rb1bt3j [37]). Node support is only shown for key nodes in the tree (complete tree is in the Dryad repository file 14: https://doi.org/10.5061/dryad.rb1bt3j [37]). All centromeric IGS sequences appear to have a single origin: they duplicated from sex-linked IGS interspersed at the rDNA loci at some time near the divergence of the simulans clade and D. melanogaster. IGS repeats in blue (extra) are similar to the IGS at 3Giglio but are on small contigs, tig00022795 and id = 102159_0. Contig tig00022795 is also moderately enriched in CENP-A. CENP-A, centromere protein A; IGS, intergenic spacer of the ribosomal genes. (TIF) [file pbio.3000241.s010.tif]

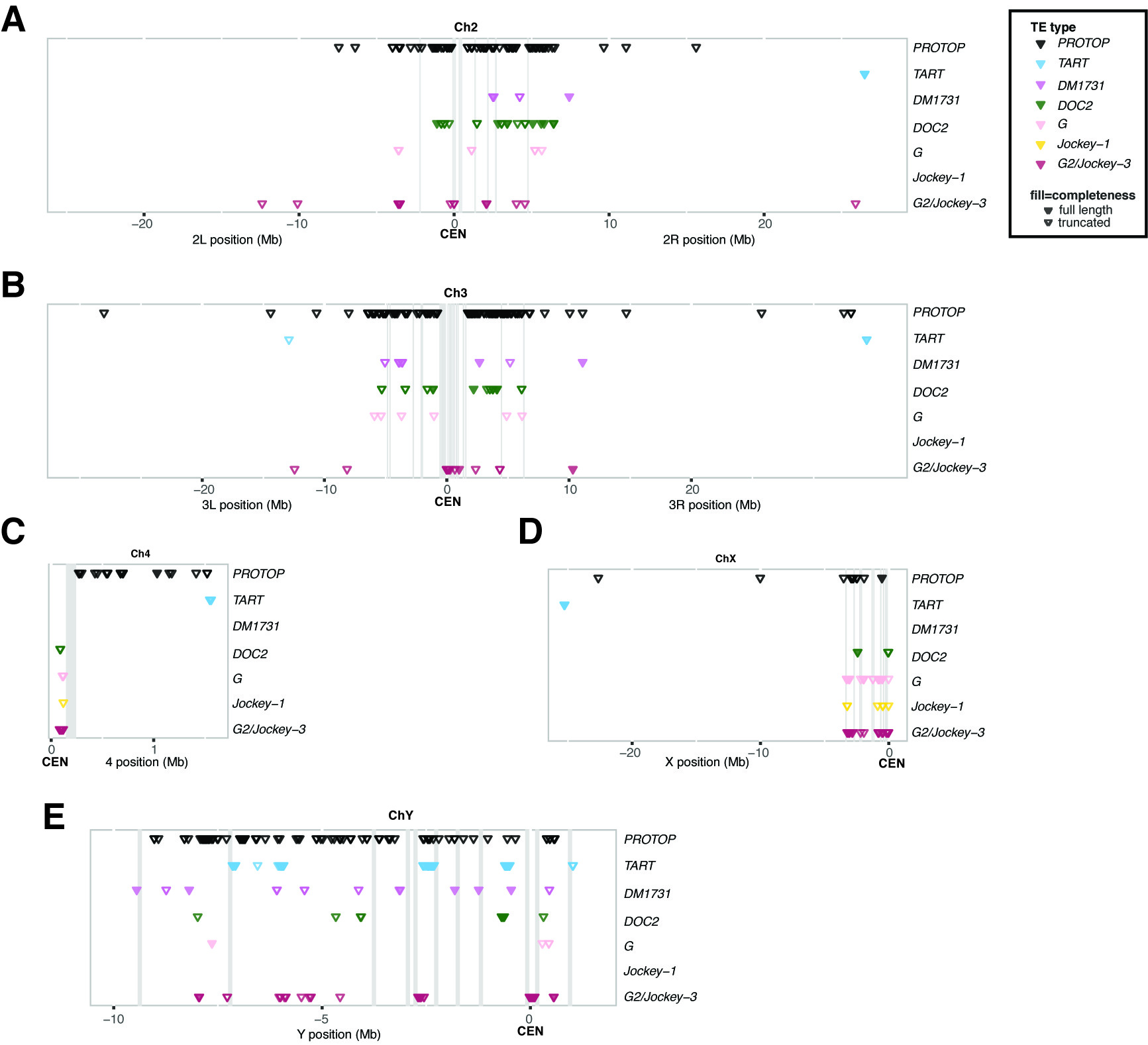

Supplement: S11 Fig — Distribution of TEs (represented by different colors) along the following chromosomes: (A) chromosome 2, (B) chromosome 3, (C) chromosome 4, (D) chromosome X, and (E) chromosome Y. Contigs from each chromosome were concatenated in order with an arbitrary insertion of 100 kb of “N.” Distances along the x-axis are approximate. The order and orientation of the Y chromosome contigs are based on gene order (see [19]). Each triangle corresponds to one TE, for which filled shapes indicate full-length TEs and open shapes indicate truncated TEs. The vertical gray bars represent the arbitrary 100-kb window inserted between contigs, indicating where we have gaps in our assembly. The centromere positions are set to 0 for each chromosome. Chromosomes are not drawn to scale (chromosome 4 and Y are enlarged). We show the genomic distribution of a sample of TEs enriched in CENP-A according to our ChIP-seq analysis (all except PROTOP). PROTOP are DNA transposons that have not been recently active, and their distribution is primarily in heterochromatin. TART elements are non-LTR retroelements highly enriched at telomeres and are also moderately CENP-A enriched. DM1731 is a retroelement moderately enriched for CENP-A but not enriched in the centromere islands. Doc2, G, Jockey-1, and G2/Jockey-3 are CENP-A enriched non-LTR retroelements abundant in the centromere islands (see S2 and S9 Tables). CENP-A, centromere protein A; ChIP, chromatin immunoprecipitation; ChIP-seq, ChIP sequencing; FISH, fluorescence in situ hybridization; IF, immunofluorescence; LTR, long terminal repeat; TART, Telomere-associated retrotransposon; TE, transposable element. (TIF) [file pbio.3000241.s011.tif]

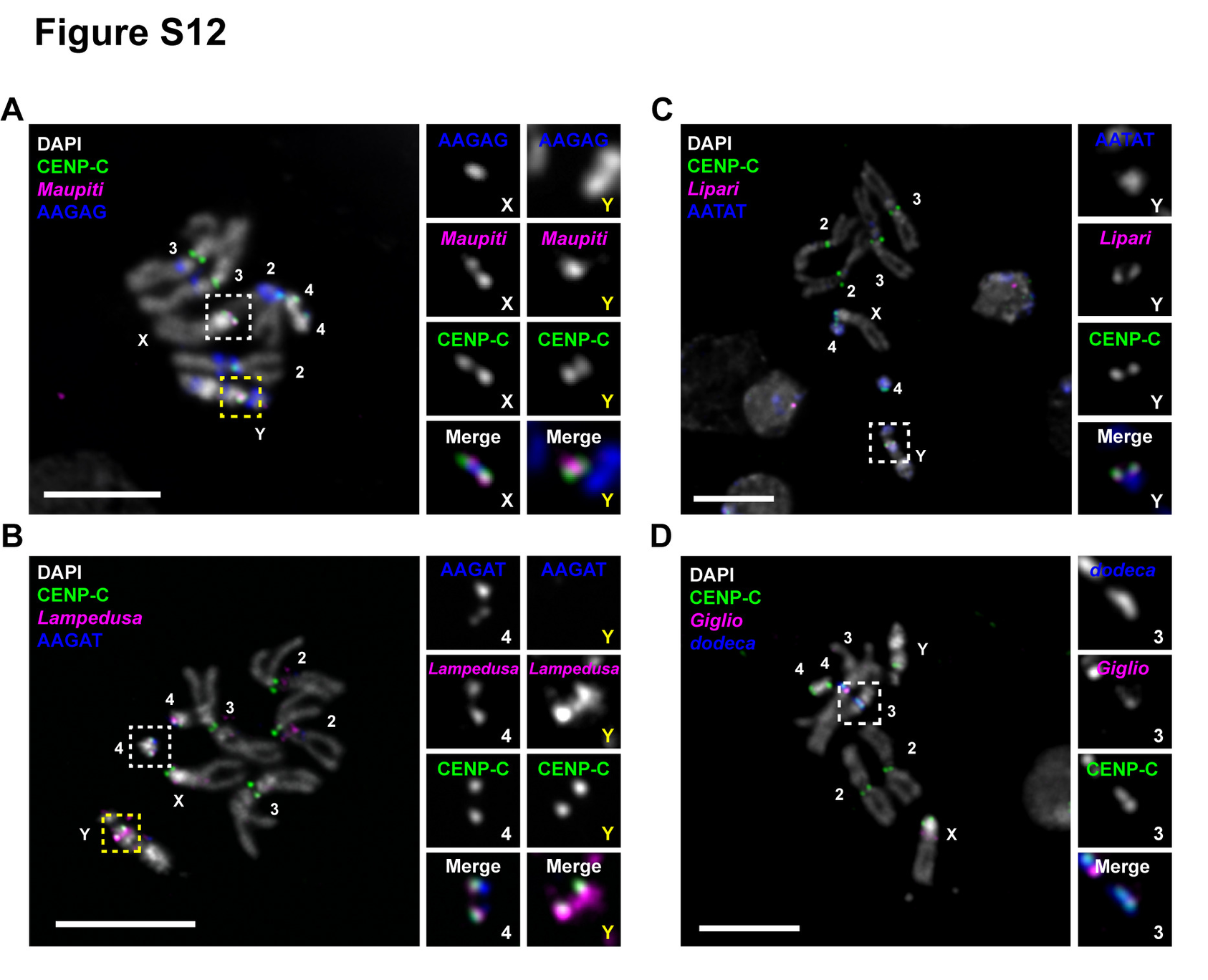

Supplement: S12 Fig — IF-FISH using an antibody for CENP-C (green), centromere Oligopaint FISH probes (magenta), and FISH probes for centromeric satellites (blue) in the following combinations: (A) Maupiti (X; magenta) and AAGAG (blue); (B) Lampedusa (4; magenta) and AAGAT (blue); (C) Lipari (Y; magenta) and AATAT (blue); (D) Giglio (3; magenta) and dodeca (blue). White boxes show the separate signals at the targeted centromeres. Yellow boxes show centromeric hybridizations at other centromeres. DAPI is shown in gray. Bar 5 μm. The underlying data for this figure can be found in S2 Data. CENP-C, centromere protein C; FISH, fluorescence in situ hybridization; IF, immunofluorescence. (TIF) [file pbio.3000241.s012.tif]

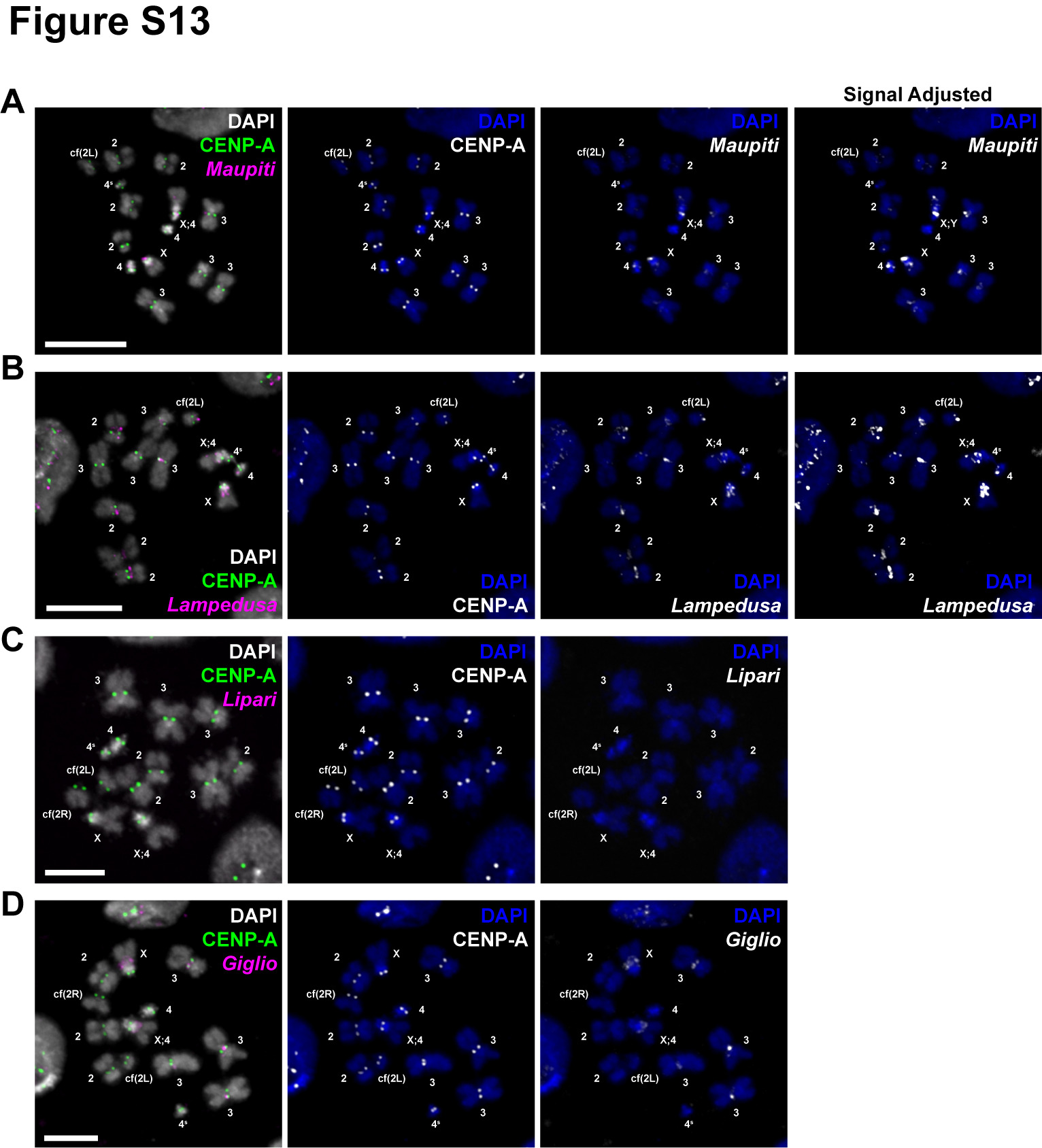

Supplement: S13 Fig — IF-FISH using an antibody for CENP-A (green) and centromere Oligopaint FISH probes designed to target centromere contigs (magenta). (A) Maupiti (X), (B) Lampedusa (4), (C) Lipari (Y), (D) Giglio (3). The “Signal Adjusted” panels in (A) and (B) show high-contrast Oligopaint hybridization for visualization of weak foci. Bar 5 μm. See also S18 Table. CENP-A, centromere protein A; FISH, fluorescence in situ hybridization; IF, immunofluorescence; S2, Schneider 2. (TIF) [file pbio.3000241.s013.tif]

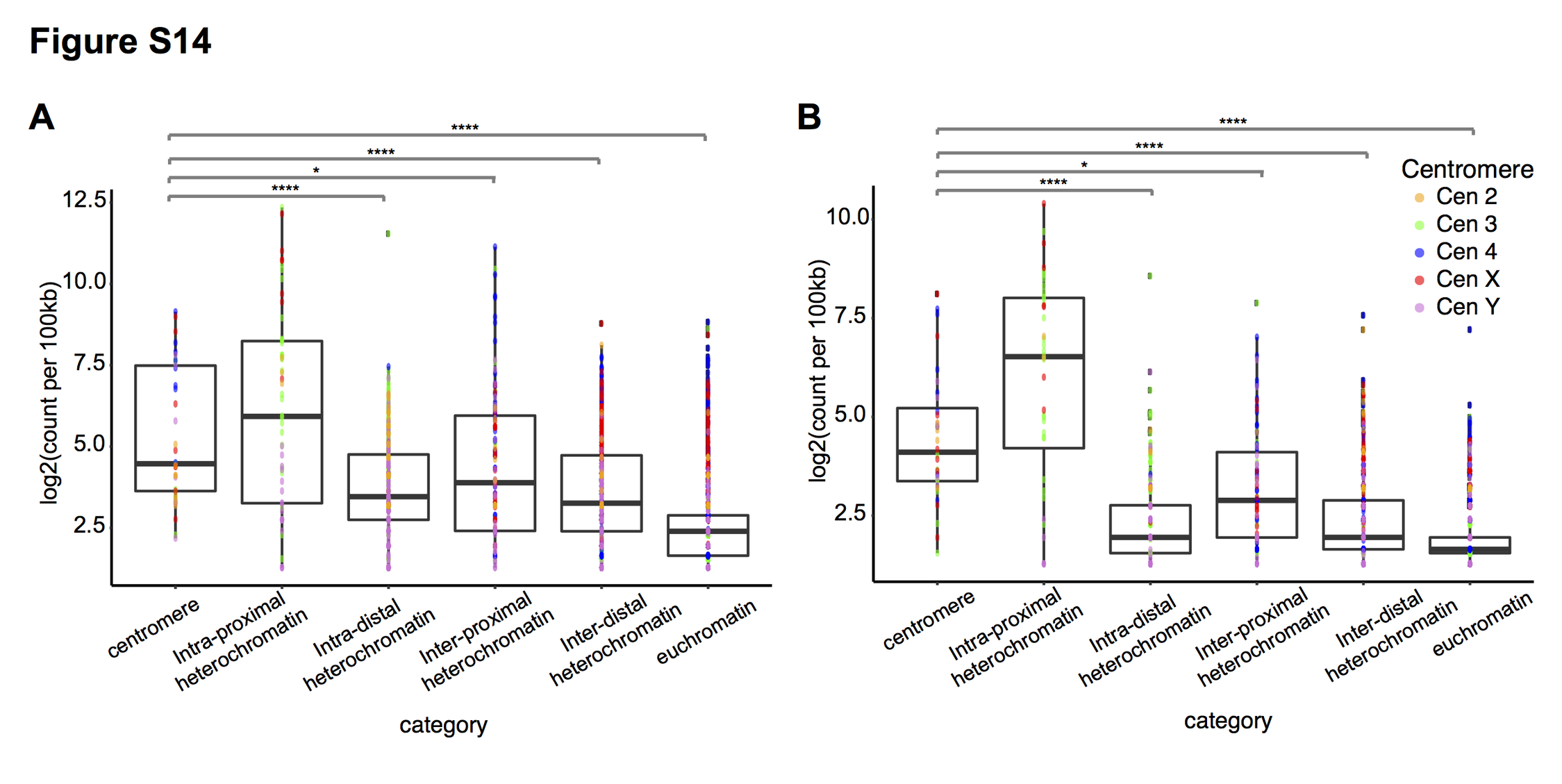

Supplement: S14 Fig — Plots showing intra- and interchromosomal interactions between regions in Hi-C data from: (A) stage 16 embryos (end of embryogenesis) and (B) embryonic cycles 1–8 (before zygotic genome activation; data from [41]). The different colors indicate interactions with individual centromeres of all chromosomes. Centromere–centromere interactions are significantly more frequent than interactions between centromeres and distal heterochromatin, interdistal heterochromatin, and euchromatin and marginally more significant than centromere–interproximal heterochromatin interactions. ****adjusted P < 0.0001; *adjusted P < 0.02, pairwise Wilcoxon rank sum test with FDR correction; Kruskal-Wallis test by ranks with Dunn’s test for post hoc analysis. The underlying data for this figure can be found in S2 Data. FDR, false discovery rate. (TIF) [file pbio.3000241.s014.tif]

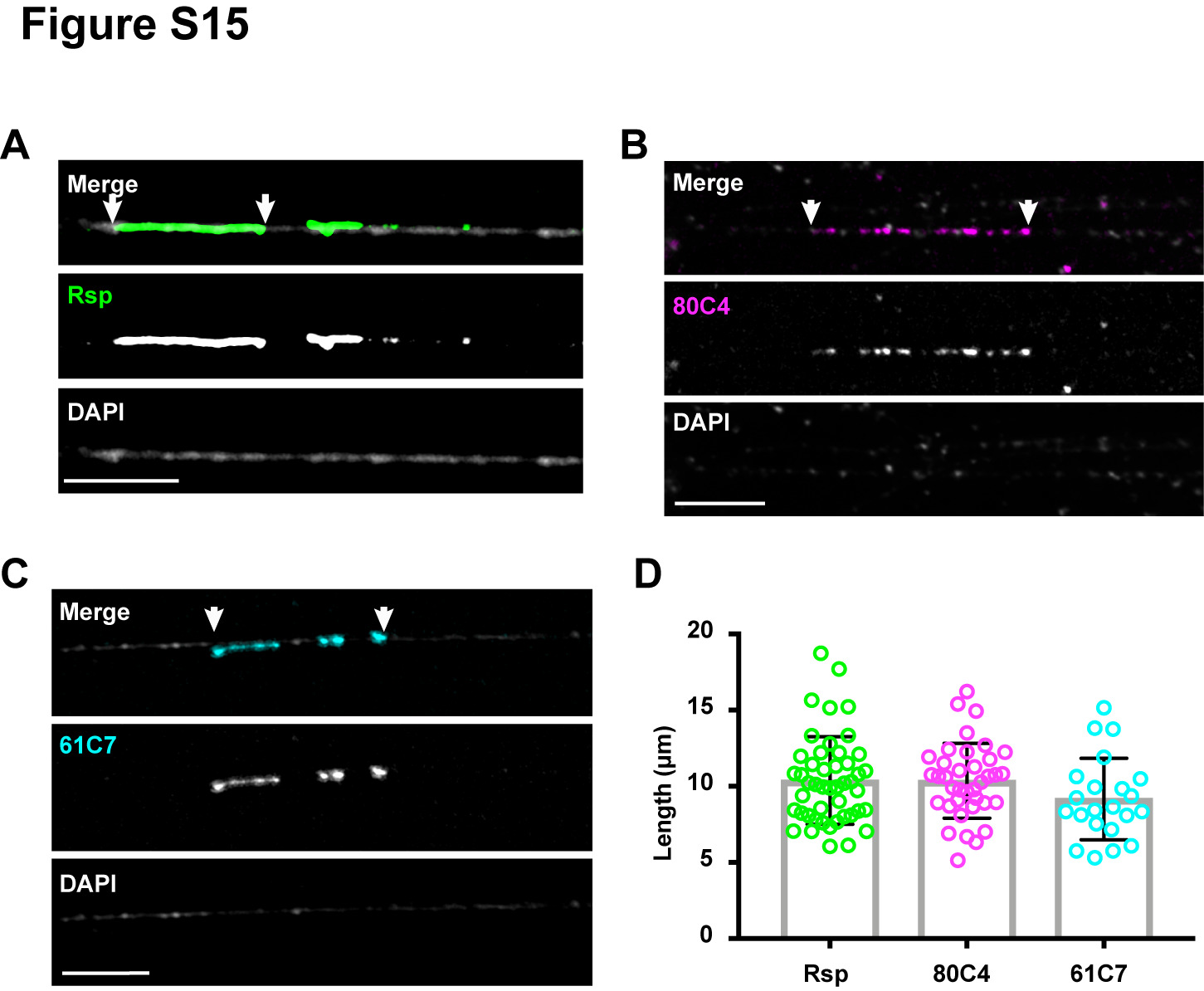

Supplement: S15 Fig — Stretched chromatin fibers from female third instar larval brain cells using the following probes: (A) Rsp locus (heterochromatic; approximately 100 kb; green); (B) 100-kb Oligopaint for a heterochromatic region on chromosome 3L (80C4; magenta); (C) 100-kb Oligopaint for a euchromatic region approximately 600 kb from the telomere of chromosome 3L (61C7; cyan). Arrows show the region of the fiber that was measured. Bar 5 μm. (D) Scatterplot showing the quantification of fiber lengths. Mean lengths were used to estimate the size in kb (approximately 10 kb/1 μm). Error bars show the standard deviation. P = 0.085 (n.s.) for each pair of measurements compared (two-tailed t test). The underlying data can be found in S2 Data. n.s., not significant; Rsp, Responder. (TIF) [file pbio.3000241.s015.tif]

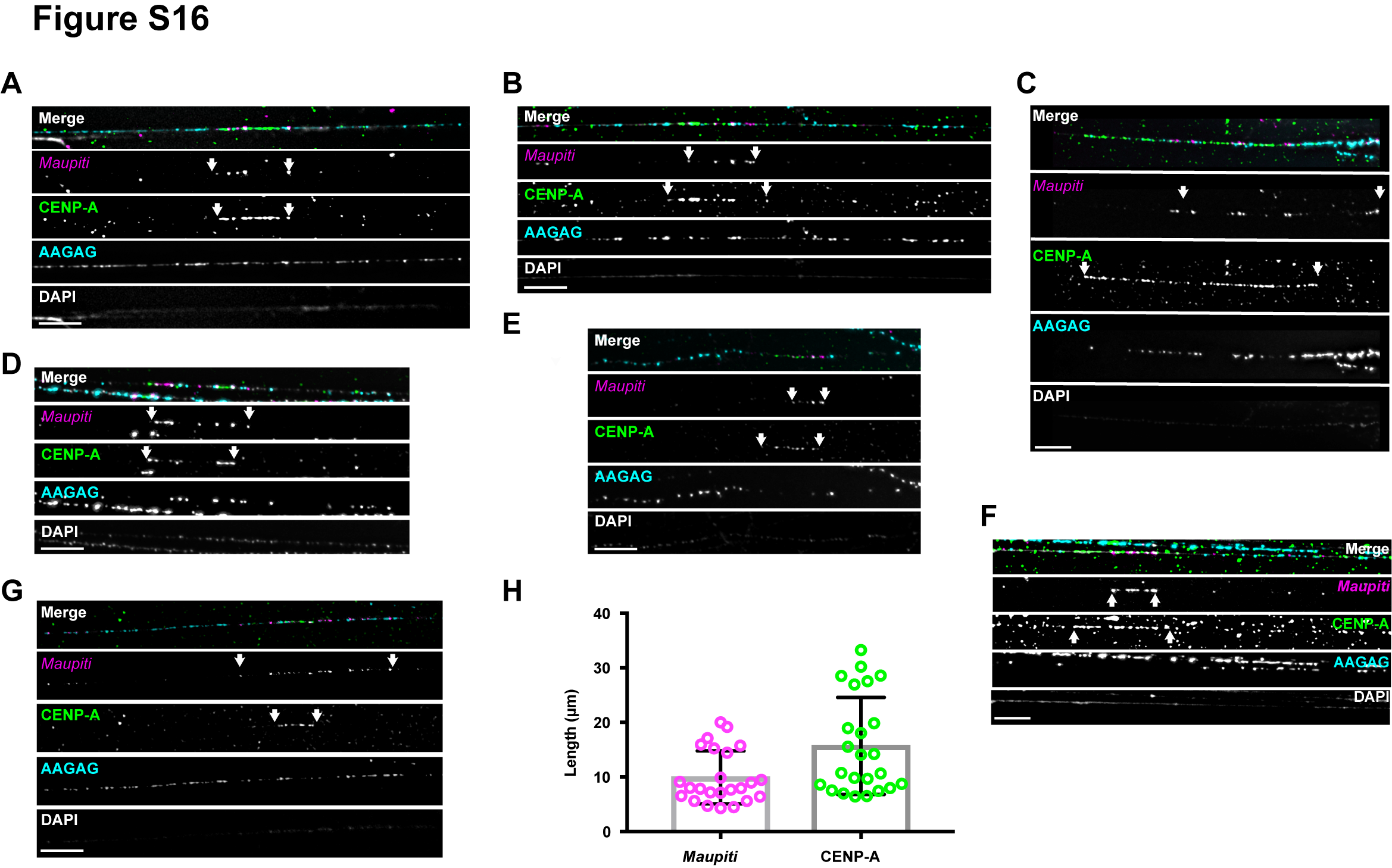

Supplement: S16 Fig — (A-G) Examples of fibers visualized with IF with anti-CENP-A antibody (green), FISH with Oligopaints for Maupiti (magenta), and AAGAG probe (cyan) on female third instar larval brain cells. DAPI is shown in gray. CENP-A occupies Maupiti and the AAGAG satellite. We observed some variation in FISH signals and Maupiti and CENP-A domain lengths, likely because of the efficiency of Oligopaint binding and variable stretching in this region. Arrows show the region of the fiber that was measured. (H) Scatterplot showing the quantification of the length of Maupiti FISH and CENP-A IF signals. Error bars show the standard deviation. N = 24 fibers. Bar 5 μm. The underlying data for this figure can be found in S2 Data. CENP-A, centromere protein A; FISH, fluorescence in situ hybridization; IF, immunofluorescence. (TIF) [file pbio.3000241.s016.tif]

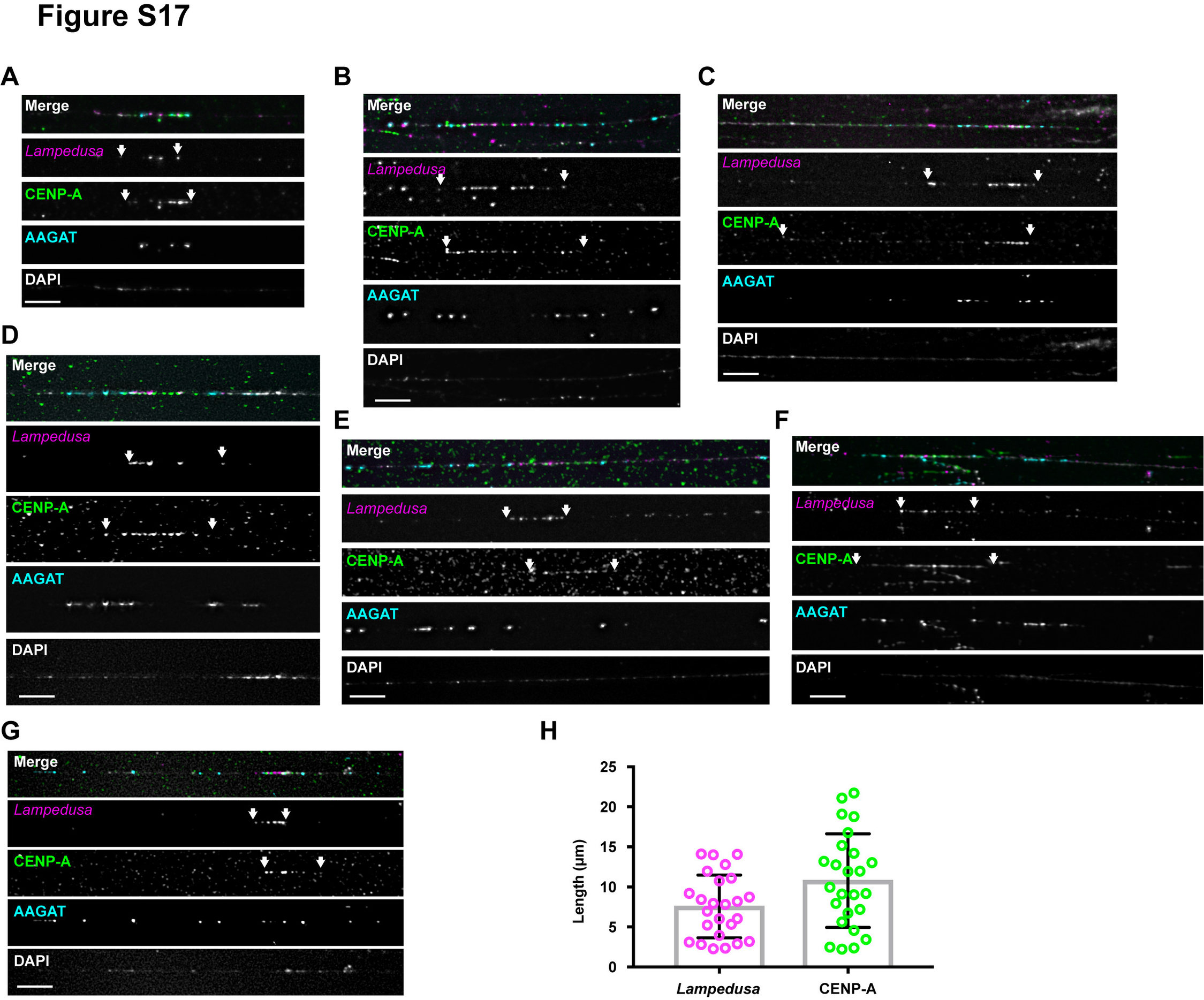

Supplement: S17 Fig — (A-G) Examples of fibers visualized by IF with anti-CENP-A antibody (green), FISH Oligopaint FISH for Lampedusa (magenta), and AAGAT probe (cyan). DAPI is shown in gray. CENP-A occupies predominantly the island Lampedusa. Arrows show the region of the fiber that was measured. (H) Scatterplot showing the quantification of the length of Lampedusa FISH and CENP-A IF signals. Error bars show the standard deviation. N = 25 fibers. Bar 5 μm. The underlying data for this figure can be found in S2 Data. CENP-A, centromere protein A; FISH, fluorescence in situ hybridization; IF, immunofluorescence. (TIF) [file pbio.3000241.s017.tif]

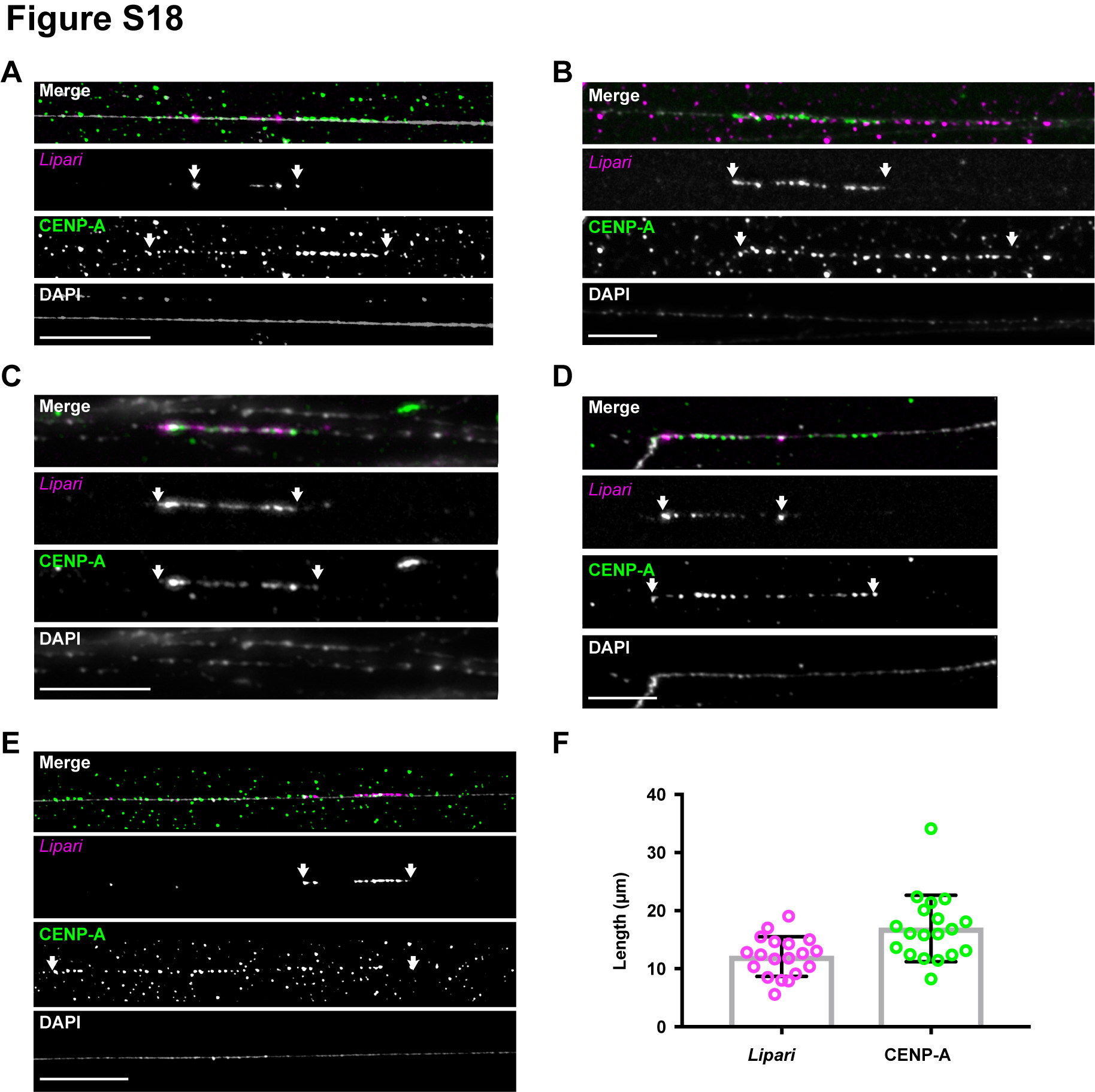

Supplement: S18 Fig — (A-E) Examples of fibers visualized by IF with anti-CENP-A antibody (green), FISH with Oligopaints for Lipari (magenta). DAPI is shown in gray. We did not include satellite FISH because no centromeric satellites are known for the Y. Note that the Oligopaints only target part of Lipari (see Fig 5). CENP-A is observed occupying sequences beyond the Oligopaint region, likely over the remaining part of the island. Arrows show the region of the fiber that was measured. (F) Scatterplot showing the quantification of the length of Lipari FISH and CENP-A IF signals. Error bars show the standard deviation. N = 19 fibers. Bar 5 μm. The underlying data can be found in S2 Data. CENP-A, centromere protein A; FISH, fluorescence in situ hybridization; IF, immunofluorescence. (TIF) [file pbio.3000241.s018.tif]

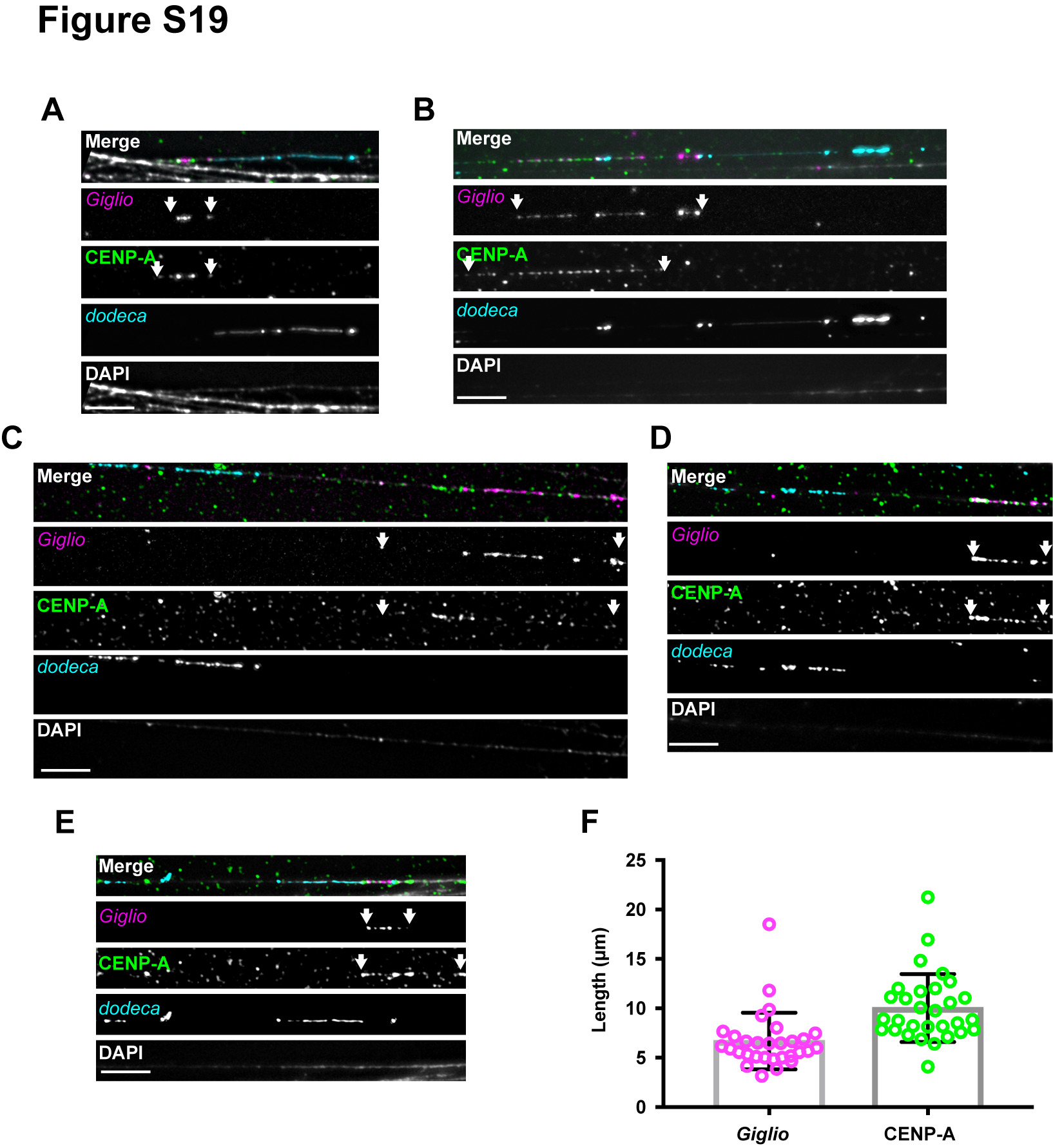

Supplement: S19 Fig — (A-E) Examples of fibers visualized by IF with anti-CENP-A antibody (green), FISH with Oligopaints for Giglio (magenta), and a probe for the centromere 3–specific dodeca satellite (cyan). DAPI is shown in gray. CENP-A occupies primarily Giglio and a small stretch of dodeca satellite. Note that the binding of the dodeca (an LNA probe) is quite variable between fibers and results in several gaps that could be a result of the higher stringency conditions needed for Giglio Oligopaint FISH. Arrows show the region of the fiber that was measured. (F) Scatterplot showing the quantification of the length of Giglio FISH and CENP-A IF signals. Error bars show the standard deviation. N = 30 fibers. Bar 5 μm. The underlying data can be found in S2 Data. CENP-A, centromere protein A; FISH, fluorescence in situ hybridization; IF, immunofluorescence; LNA, locked nucleic acid. (TIF) [file pbio.3000241.s019.tif]

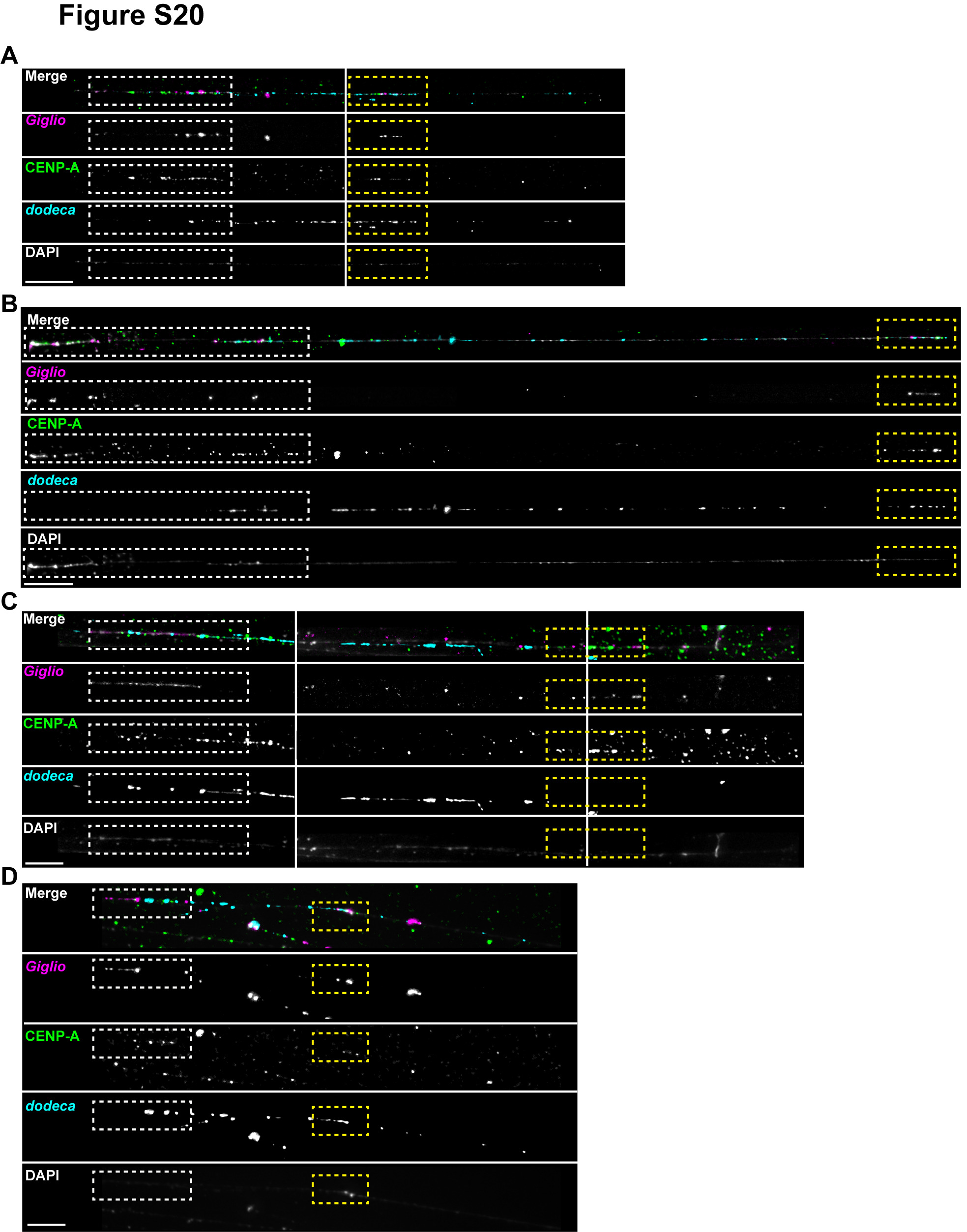

Supplement: S20 Fig — (A-D) Examples of longer fibers tracked along dodeca from the experiment in S19 Fig, visualized by IF with anti-CENPA antibody (green), Oligopaint FISH for Giglio (magenta), and FISH with dodeca probe (cyan). DAPI is shown in gray. Note the presence of Giglio signal on the dodeca CENP-A region. Multiple, overlapping panels were often acquired to follow an individual fiber. Panels were then cropped and juxtaposed in the figure, with white lines showing the separate images. White boxes show the CENP-A domain on Giglio, and yellow boxes show the smaller domain on dodeca. N = 5 (these are rare fibers to find in our preparations because of their length). Bar 5 μm. CENP-A, centromere protein A; FISH, fluorescence in situ hybridization; IF, immunofluorescence. (TIF) [file pbio.3000241.s020.tif]

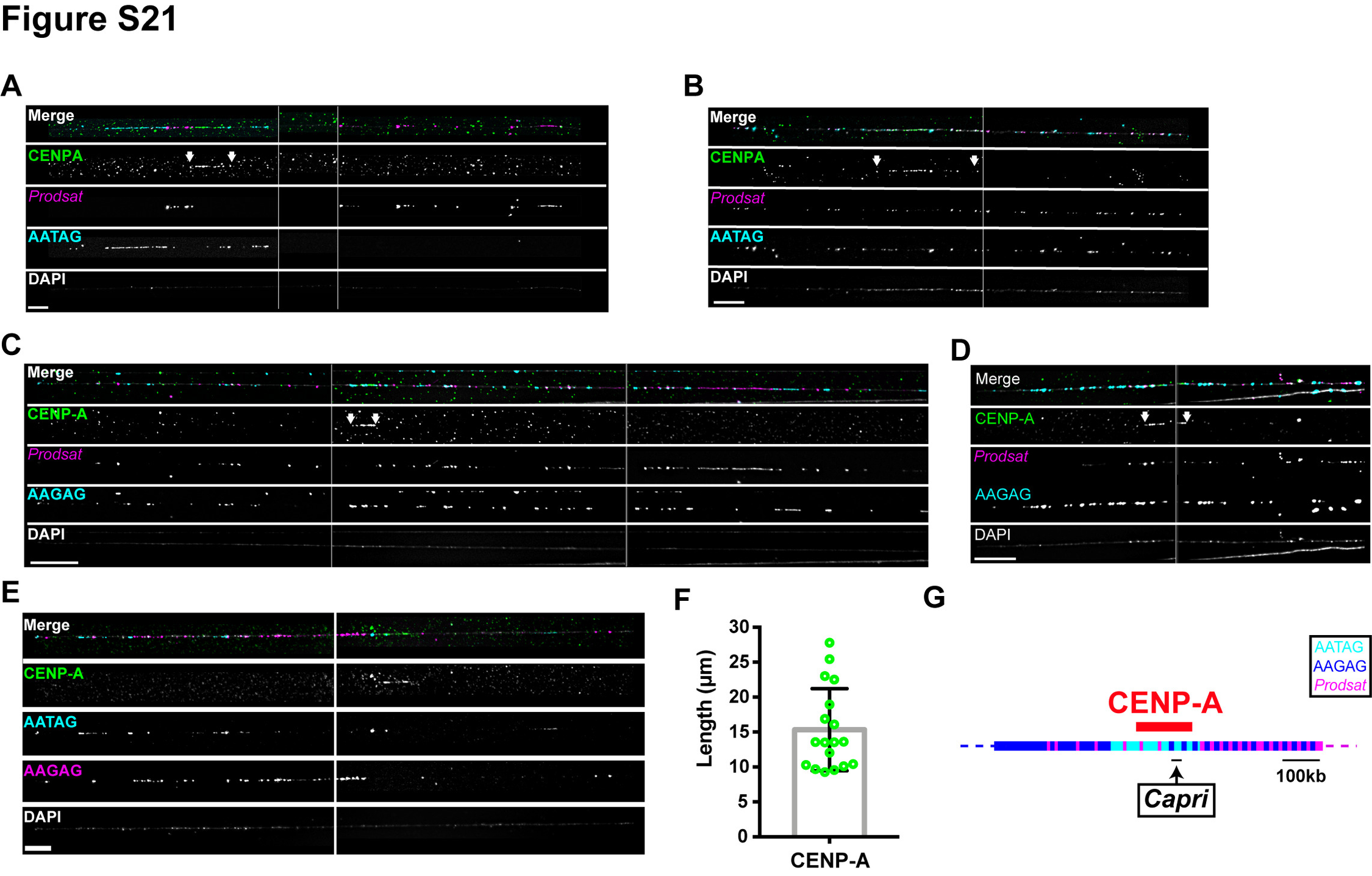

Supplement: S21 Fig — (A-D) Examples of fibers visualized with IF with anti-CENP-A antibody (green) and FISH with satellites. DAPI is shown in gray. (A-B) Examples of fibers showing colocalization of CENP-A (green) with Prodsat (magenta) and AATAG (cyan). (C-D) Examples of fibers with AAGAG (cyan) and Prodsat (magenta). (E) Example of fiber with AAGAG (magenta) and AATAG (cyan). We propose that Capri is located between flanking blocks of AAGAG and AATAG satellites that reside very close to where the Prodsat begins. Arrows show the region that was measured for each fiber. (F) Scatterplot of CENP-A IF signal lengths. (G) Model for the organization of centromere 2 showing a possible location of Capri. Error bars show the standard deviation. N = 18 fibers. Bar 5 μm. The underlying data can be found in S2 Data. CENP-A, centromere protein A; FISH, fluorescence in situ hybridization; IF, immunofluorescence; Prodsat, Prod satellite. (TIF) [file pbio.3000241.s021.tif]
