# Supplementary material for: Islands of retroelements are major components of Drosophila centromeres
Source: PLoS Biol. 2019 May 14;17(5):e3000241. doi: 10.1371/journal.pbio.3000241 (PMC6516634; doi:10.1371/journal.pbio.3000241)
Supplement: S1 Text — (DOCX) [file pbio.3000241.s044.docx]

**S1 Text**

**Table of contents**

Effects of library preparation 48

Non-centromeric CENP-A associations 49

Centromere-associated repeat evolutionary genomics 49

Centromeric IGS 49

G2/Jockey-3 50

Analysis of inter-centromere interactions by Hi-C 51

Localization of satellites on iso-1 mitotic chromosomes 51

Localization of Oligopaint FISH probes on iso-1 mitotic chromosomes 52

Localization of Oligopaint FISH probes on S2 cells mitotic chromosomes 52

Localization of satellites on S2 cells mitotic chromosomes 53

*Drosophila simulans* centromeric *G2/Jockey-3* 54

## Effects of library preparation

Centromeres are enriched in repetitive sequences, therefore CENP-A ChIP-seq may be particularly sensitive to biases due to different library preparations. We carried out four replicate ChIP-seq experiments: two with TruSeq (R1 and R4) and two with Accel-NGS 2S Plus (R2 and R3). The Accel-NGS 2S plus library kit requires less starting material, requires fewer PCR cycles, and is reported to lead to higher library complexity. Our mapping rate to the genome is >98% except for R1 (57%), which has bacterial read contamination from *Brevundimonas*. R2 and R3 have a higher reproduction rate in MACS ChIP peak calls than the R1 and R4 TruSeq libraries (S3 Fig and S4 Table). To determine if the non-centromeric peaks were truly enriched in CENP-A, we used ChIP-qPCR. We did not detect an enrichment of CENP-A over a subset of the strongest non-centromeric peaks using qPCR (S5 Fig). We therefore think that most of these non-centromeric peaks represent noise from library bias rather than true signal. The signal-to-noise ratio in the TruSeq libraries was higher than the Accel libraries (S3 Fig). The CENP-A ChIP data from Talbert et al. 2018 [1] were obtained from independent sources (S2 cells and CID/CENP-A-GFP embryos), with a different ChIP method (*i.e.* MNase digestion) and library preparation. The results largely agree between these independent datasets (S4 Table and S3B Fig).

Among centromeres and across replicates, Contig119 (Cen4 candidate) and Y_Contig26 (CenY candidate) consistently appear in the top three contigs with the strongest signals in the IDR tests (S4 Table). This is consistent with published cytological data showing the strongest CENP-A signal on the 4^th^ and Y chromosomes [2]. S2 cells lack a Y chromosome, therefore it is reassuring that we cannot detect CenY in the top 100 strongest signals in any S2 dataset. Outside of missing CenY, the CENP-A ChIP in S2 cells differs from the embryo ChIP, suggesting possible centromere rearrangements in S2 cells. We only detect weak signals from Contig79 (CenX candidate; *Maupiti*) in S2 cells and the MNase treated CENP-A ChIP data from Talbert et al. [1] (S4 Table and S3B Fig). We are unsure of the reasons for the weaker CenX signal in some datasets, however it is possible that our results are sensitive to ChIP and library preparation methods. Additionally, S2 cells have large-scale genomic rearrangements [3] that likely affect their centromeres.

## Non-centromeric CENP-A associations

While most of the strongest CENP-A peaks in embryos are in our centromere islands, we do find significant peaks at other sites throughout the genome. The two replicate Accel libraries show far more peaks outside of the centromere islands than the two replicate TruSeq libraries (S4 Table and S3A Fig). However, many of the non-centromeric peaks were not reproducible between replicates using different library methods (S8 Table). We therefore only focused on peaks overlapping in two of four replicate CENP-A ChIP-seq experiments as potentially interesting sites (S4 Table). We do not consistently find CENP-A enrichment on non-centromeric *G2/Jockey-3* elements (S8 Table). Notably, some transposable elements not found on the centromere islands were consistently enriched in the CENP-A ChIP replicates (Fig 1C and S2 Table)—among enriched non-centromeric sites are telomeric non-LTR retroelements and R1 elements. While many of these associations could represent non-specific peaks [4], there may be precedent for an association with the rDNA. The CENP-A assembly factor CAL1 [5] localizes at the nucleolus as well as at the centromere [6], therefore these reads could represent the fraction of CENP-A associated with nucleolar CAL1.

While functional kinetochores are unlikely to form on the regions of non-centromeric CENP-A enrichment, these sites might be poised for neo-centromere function. We hypothesized that a subset of the non-centromeric CENP-A-enriched regions would also be identified in CENP-A overexpression data. We therefore compared published CENP-A ChIP in CENP-A overexpression data from S2 cells [7] with our S2 cells ChIP data. Overall, there are only four contigs that overlap with our S2 data using standard peak-calling methods: Cen2, Cen3, one contig with *dodeca* and *Prodsat,* and one contig with mostly *1.688* satellite and a *Max* transposon (IDR ≤ 0.05; S17 Table). The original study used custom peak calling analysis that binned regions to call large, lowly enriched peaks, which could explain these differences. Most of the regions of CENP-A enrichment in the S2 cells overexpression data were in contigs almost exclusively filled with *dodeca* (*e.g.* Contig86) and variants of the *1.688* family of repeats (*e.g.* Contig102 and 96). These contigs are proximal to canonical centromeres. Because *dodeca* is also part of the canonical 3^rd^ chromosome centromere and the pericentromeric *1.688* repeats associate with the kinetochore-specific protein BUB1, in prometaphase [8], these repeats may have some properties of centromeric DNA. In addition, both *dodeca* and *1.688* repeats can assume non-B form DNA structures [9]. We therefore hypothesize that when CENP-A is overexpressed, the centromeres expand to include proximal pericentric heterochromatin and non-B form DNA [9, 10].

## Centromere-associated repeat evolutionary genomics

### Centromeric IGS

Centromeres are enriched for repetitive elements whose locations are not exclusive to centromeres (e.g. IGS and *G2/Jockey-3*). To determine if there are centromere-specific variants or if CENP-A recognizes and binds to a particular part of those repeats, we explored the evolutionary relationships between each repeat copy across the genome. The IGS^3cen^ sequences represent a duplication from the IGS sequences that are spacers between the rDNA genes on the sex chromosomes. The IGS^3ce^*^n^* repeats at 3*^Giglio^* and IGS^extra^ repeats at two contigs, tig00022795 and id=102159_0 form their own distinct clade (S10 Fig and S4 Table; see Dryad repository File 14: https://doi.org/10.5061/dryad.rb1bt3j [11]). These two contigs also contain *Prodsat* and tig00022795 contains a *G2/Jockey-3* element, suggesting they are either pericentric or partly centromeric. The contig tig00022795 is moderately enriched for CENP-A in our ChIP-seq analyses. Interestingly, we also detected CENP-A signal in extended fiber-FISH analyses near 3^Giglio^ (S20 Fig). This signal may correspond to tig00022795. The IGS duplication leading to the centromere-enriched IGS variants happened near the divergence between *D. melanogaster* and the *D. simulans* clade. However, IGS^3cen^ is not found in the simulans clade. We then infer that IGS^3cen^ is a derived centromere and the expansion of IGS^3cen^ is associated with the acquisition of its CENP-A binding ability.

### *G2/Jockey-3*

We looked for orthologs of *G2* in other Drosophila species. We find orthologs of *G2* in the simulans clade, *D. erecta,* and *D. yakuba,* but not more distantly related species. Reciprocal best BLAST hits suggest that *G2* is a *Jockey*-type element orthologous to *Jockey-3* in *D. simulans*. *Jockey-3* is not annotated in *D. melanogaster* (according to Repbase), but RepeatMasker does annotate *Jockey-3* in our genome using the *D. simulans* *Jockey-3* consensus. We inferred the phylogeny of the 36 *Jockey-3* and 57 *G2* annotations >1 kb in *D. melanogaster* and the consensus of *Jockey-3* from *D. simulans, D. sechellia,* and *D. yakuba* using maximum likelihood methods (see Materials and Methods). Our phylogenetic analysis suggests that in *D. melanogaster* *G2* and *Jockey-3* are the same element (S2 Fig; see Dryad repository File 15: https://doi.org/10.5061/dryad.rb1bt3j [11]). Most of the *G2* elements are truncated at the 5’ end. CENP-A does not pileup over a particular part of *G2* (based on the ratio of ChIP/Input across the full length of a *G2* element; Fig 6A and 6B). *D. melanogaster*’s version of *Jockey-3* contains an additional 1,102 bp with a predicted ORF upstream of the 5’ end of *G2* annotations. We do not know what is required for *G2/Jockey-3* activity, we therefore define ‘complete’ elements as those containing intact ORFs encoding an endonuclease and a reverse transcriptase regardless of length and combine the 235 *G2* annotations and 63 *Jockey-3* annotations in our analyses giving us a total of 298 *G2/Jockey-3* partial and full-length elements. We believe that the first 487 bp of *D. simulans Jockey-3* consensus sequence is misannotated and instead belongs to a different repeat (see section on *D. simulans* centromeric *Jockey-3* below).

*G2/Jockey-3* elements are not randomly distributed in the genome, they are concentrated at centromeres [enrichment in centromeric contigs (63%) relative to other heterochromatin (31%; FET with FDR correction P < 10^-15^; S9 Table, Fig 4 and S11 Fig]. We find evidence for *G2/Jockey-3* transcription among poly-A and total RNA-seq reads (S9 Fig; see Materials and Methods). *G2/Jockey-3* insertions are polymorphic in populations of *D. melanogaster* [12]. Moreover, most *G2/Jockey-3* elements are truncated at the 5’ end but are otherwise intact, which is a hallmark of non-LTR insertion (see Dryad repository File 13: https://doi.org/10.5061/dryad.rb1bt3j [11]). Taken together, these results suggest that *G2/Jockey-3* are recently or currently active elements, consistent with a recent study of active elements in oogenesis [13]. The enrichment of *G2/Jockey-3* at centromeres is not a typical genomic distribution for recently active TEs. We plotted the distribution of other TEs enriched in CENP-A according to our ChIP-seq analysis (*Jockey*-1; *G*; *Doc2*; *DM1731; R1; TART*), and inactive TE (*ProtoP*) (S11 Fig). Besides *G2/Jockey-3*, *G* (FET with FDR correction P = 0.013), *Doc2* (FET with FDR correction P < 10^-7^) and *Jockey-1* (FET with FDR correction *P* = 0.013) are also enriched at centromeres compared to other heterochromatin regions (S9 Table).

## Analysis of inter-centromere interactions by Hi-C

We asked how often centromere island contigs make contacts with other compartments of the genome in the 3D nucleus using Hi-C data from two different embryonic stages (cycle 1-8 and stage 16). We first assigned each contig to one of the following genomic regions: centromere, centromere-proximal heterochromatin, centromere-distal heterochromatin, and euchromatin. We recorded the number of contacts between centromere contigs and other centromere contigs (centromere-centromere category). We then recorded the number of contacts between centromeres and euchromatin, proximal, or distal heterochromatin on the same chromosome; and between centromeres and other chromosome’s euchromatin, proximal, or distal heterochromatin. We compared the distribution of contacts in the centromere-centromere category to the other types of intra- and inter-chromosomal interaction categories involving centromeres (S16 Table). We tested for differences between the numbers of contacts in each of these categories using a Kruskal Wallis test, with a post-hoc Dunn’s test to ask if the centromere-centromere interaction category differ from each of the other intra- and inter-chromosome categories involving centromeres. We report P values adjusted based on false discovery rate (FDR) [14]. We find significant differences across the inter- and intra-chromosomal interactions categories involving centromeres (Kruskal Wallis P < 10^-16^; S14 Fig). The categories with the most frequent centromere interactions are centromere-centromere and intra-chromosomal centromere to proximal heterochromatin, although these two categories do not significantly differ from each other (Dunn’s P_adjusted_ = 0.59 for embryonic cycle 1-8; Dunn’s P_adjusted_ = 0.43 for embryonic stage 16). Intra-chromosomal centromere to distal heterochromatin (Dunn’s P_adjusted_ < 10^-11^ for embryonic cycle 1–8, Dunn’s P_adjusted_ < 10^-3^ for embryonic stage 16), inter-chromosomal centromere to proximal heterochromatin interactions (Dunn’s P_adjusted_ = 0.0088 for embryonic cycle 1–8; Dunn’s P_adjusted_ = 0.017 for embryonic stage 16), and inter-chromosomal centromere to distal heterochromatin interactions (Dunn’s P_adjusted_ < 10^-8^ for embryonic cycle 1–8; Dunn’s P_adjusted_<10^-4^ for embryonic stage 16) show similar frequencies. The least frequent interactions are between centromeres and euchromatin (Dunn’s P_adjusted_ < 10^-19^ for embryonic cycle 1–8; Dunn’s P_adjusted_ < 10^-19^ for embryonic stage 16).

## Localization of satellites on iso-1 mitotic chromosomes

We determined the position and organization of centromere-associated satellite DNA within the iso-1 genome using satellite specific FISH probes (see Materials and Methods and S11 and S12 Tables) and an anti-CENP-C antibody to mark the centromeres (S7 Fig). On the X chromosome, CENP-C overlaps with AATAT (100%, N = 27 spreads) and partially with AAGAG (56.3%, N = 16 spreads) (S7E and S7F Fig). *SATIII* (100%, N = 34 spreads) (S7E Fig) and a small block of AAGAT (26.7%, N = 30 spreads) (see high contrast inset on S7A Fig) are adjacent to CENP-C on the X, thus they are categorized as pericentric. On chromosome 2, AAGAG (100%, N = 16 spreads) and *Prodsat* (100%, N = 12 spreads) overlap with CENP-C (S7B Fig). Chromosome 2 also contains two blocks of AATAG, with one small block overlapping with CENP-C (100%, N = 38 spreads) (S7C and S7G Fig; white arrow) and one large block in heterochromatin (100%, N = 38 spreads) (S7C and, S7G Fig; yellow arrow). On chromosome 3, CENP-C overlaps with *dodeca* (100%, N = 12 spreads) (S7D Fig) and is adjacent to *Prodsat* (100%, N = 12 spreads) (S7B, S7D and S7G Fig). A pericentric block of AATAG can also be found on chromosome 3 (100%, N = 38 spreads) when FISH is performed without the *Prodsat* FISH probe (S7C Fig). On chromosome 4, CENP-C overlaps with AAGAT (100%, N = 30 spreads) (S7A Fig) and is flanked by AAGAG (100%, N = 16 spreads) (S7A, S7B, S7C and S7F Fig) and AATAT (100%, N = 27 spreads) (S7E and S7F Fig). On the Y, AATAT is the closest satellite to CENP-C (S7E and S7F Fig), but it does not overlap with CENP-C. Refer to Table 1 in the main manuscript for the position of satellites within heterochromatin.

## Localization of Oligopaint FISH probes on iso-1 mitotic chromosomes

We validated the positions of the X*^Maupiti^*, 4*^Lampedusa^*, Y*^Lipari^*, and 3*^Giglio^* using Oligopaint FISH on mitotic spreads from the iso-1 line. X*^Maupiti^* probe signal overlapped with CENP-C and AAGAG on the X chromosome (100%, N = 31 spreads), recapitulating the organization seen in Contig 79 (S12A Fig; white box). Interestingly, X*^Maupiti^* Oligopaint also overlaps with CENP-C, but not AAGAG on the Y chromosome (93.5%, N = 31 spreads) (S12A Fig; yellow box). The 4*^Lampedusa^* Oligopaint overlaps with CENP-C and is next to AAGAT (100%, N = 22 spreads; S12B Fig; white box), confirming that Contig119 corresponds to centromere 4. 4*^Lampedusa^* also hybridized to the Y chromosome’s centromere (100%, N = 22 spreads), which is devoid of AAGAT repeats (S12B Fig; yellow box). The Y*^Lipari^* Oligopaint overlaps with CENP-C specifically on the Y chromosome (100%, N = 14 spreads) (S12C Fig; white box). The closest satellite to the Y centromere is AATAT, however, this satellite does not overlap with CENP-C or Y*^Lipari^* (S12C Fig; blue; white box). The 3*^Giglio^* Oligopaint overlaps with CENP-C and *dodeca* on chromosome 3 (100%, N = 34 spreads) (S12D Fig; white box), consistently with the organization of contig 3R_5. 3*^Giglio^* Oligopaints weakly hybridize to the rDNA on the X chromosome (38.2%, N = 34 spreads), away from CENP-C (S12D Fig).

## Localization of Oligopaint FISH probes on S2 cells mitotic chromosomes

All centromere islands (*e.g.* Fig 2) are enriched in S2 cells (S4 Table and S3B Fig) except for Y*^Lipari^*, consistent with the Y chromosome being absent from S2 cells. However, we find some large differences between CENP-A domains in S2 cells and embryos. CENP-A is enriched in additional transposable elements (*e.g*. *Max-I*; S2 Table) and strongly enriched in some simple tandem repeats (*e.g*. *Prodsat* and *AATAG*, S1 Table) in S2 cells compared to the embryo ChIP-seq data using the same library method (R4).

We determined whether the positions of X*^Maupiti^,* Y*^Lipari^,* 3*^Giglio^*, and 4*^Lampedusa^* in the iso-1 genome had been maintained in S2 cells using our Oligopaints for FISH and IF with an antibody for CENP-A to mark the centromere (S13 Fig; green). DAPI staining combined with FISH revealed the S2 genome consisted of the canonical chromosomes X, 2, 3, and 4. In addition, S2 cells also contained a Robertsonian translocation between X and 4 (chromosome X;4), two centric fragment chromosomes generated most likely by a break within the centromere of chromosome 2 (designated cf(2R) and cf(2L)), and a small chromosome 4 (chromosome 4^s^) (S8 and S13 Figs and S18 and S19 Tables). The X*^Maupiti^* Oligopaint overlaps with CENP-A on the X;4 chromosome and is adjacent to CENP-A on the X chromosome, indicating X*^Maupiti^*’s position on X;4 is conserved with iso-1, but CENP-A may have shifted on the normal X chromosome. Interestingly, the X*^Maupiti^* Oligopaint also hybridizes to the centromere of chromosome 3 (see “Signal Adjusted” panel) and other heterochromatic loci on the X, chromosomes 2, cf(2R), cf(2L), and 4 (S13A Fig), suggesting that transposable elements within X*^Maupiti^* may have remobilized in S2 cells. The 4*^Lampedusa^* Oligopaint hybridizes to loci adjacent to CENP-A on cf(2L), the X and X;4 chromosomes, and is heterochromatic on chromosomes X, X;4, 2, cf(2R), 3, and 4 (S13B Fig). Surprisingly, the 4*^Lampedusa^* Oligopaint overlaps with CENP-A on chromosomes 2, cf(2R), and 3, but not on chromosomes 4 and 4^s^ (S13B Fig). These data suggest that like X*^Maupiti^*, 4*^Lampedusa^* elements have also remobilized and integrated into these chromosomes. On normal chromosome 4 the signals for 4*^Lampedusa^* and that of CENP-C do not overlap, so it is possible that the centromere has repositioned. In the case of chromosomes 4^s^, the lack of 4*^Lampedusa^* centromeric signal suggests that the centromere was lost, perhaps via a translocation with chromosome X;4. Unlike X*^Maupiti^* and 4*^Lampedusa^*, the Y*^Lipari^* Oligopaint shows no hybridization to anywhere in the S2 genome (S13C Fig), consistent with the ChIP-seq results. Lastly, the 3*^Giglio^* Oligopaint hybridizes to the centromere and pericentromere of chromosome 3, as well as the heterochromatic locus associated with rDNA of the X and X;4 chromosomes (S13D Fig), indicating that 3*^Giglio^*’s position has been retained between iso-1 and S2 cells (S12D Fig). Additionally, the pericentric position of 3*^Giglio^* suggests that the CENP-A domain on a subset of chromosome 3’s has shifted. See S18 Table for quantification of Oligopaint hybridization locations in S2 cells.

## Localization of satellites on S2 cells mitotic chromosomes

To determine whether the positions of centromere associated satellite sequences have been maintained from iso-1 flies to S2 cells, we performed FISH for centromeric satellites on S2 mitotic chromosomes using an anti-CENP-A antibody to mark the centromere (S8 Fig; green). Consistent with the X chromosome of iso-1 (S7E and S7F Fig), the centromeres of the X and X;4 chromosomes are flanked by *SATIII* on the long arm and AATAT (S8A Fig), AAGAG (S8D, S8E and S8F Fig), and AAGAT (S8F Fig) on the short arm. On chromosome 2, CENP-A overlaps with AAGAG (S8D, S8E and S8F Fig), AATAG (S8C and S8E Figs), and *Prodsat* (S8B, S8C and S8D Fig), consistent with iso-1’s chromosome 2 (S7B, S7C and S7G Fig), but has also gained AATAT repeats in all chromosome 2’s (S8A Fig) and has lost AAGAT on all but one chromosome 2 (S8F Fig). The centromere of cf(2R) overlaps with AAGAG (S8B Fig) and AATAG, with a small block of *Prodsat* adjacent to CENP-A (adjusted signal inset of S8C Fig). The centromere of cf(2L) overlaps with AAGAG and *Prodsat* (S8B, S8C and S8D Figs) and is flanked by two blocks of AATAG (S8C Fig). Blocks of AATAT (see cf(2R) and cf(2L) in S8A Fig) and AAGAT (see cf(2R) in S8F Fig) are in the pericentric heterochromatin of cf(2R) and cf(2L). As is seen on iso-1 chromosome 3’s, the centromere of the S2 chromosome 3 overlaps with *dodeca* (S8B Fig) and *Prodsat* (S8B, S8C and S8D Fig) and contains a pericentric block of AATAG only visible when not combined with *Prodsat* FISH probes (see adjusted signal inset on S8E Fig). Chromosome 4 and 4^s^’s centromeres overlap with AATAT (S8A Fig), contain AAGAG within the pericentric heterochromatin (S8E and S8F Fig), and AAGAT is present on the heterochromatin of chromosome 4 but absent on chromosome 4^s^ (S8F Fig). One explanation for the loss of AAGAT on S2 chromosome 4^s^ is that the AAGAT on X;4 was inherited from the S2 chromosome 4^s^ via a Robertsonian translocation. As for chromosome 4, the movement of AAGAT away from the centromere could have occurred via a pericentric inversion with breakpoints in AATAT and AAGAT, which either left behind the original centromere surrounded by an amount of AAGAT undetectable by FISH or inactivated the endogenous centromere within AAGAT and activated a new centromere within AATAT. See S18 and S19 Tables for detailed quantification of all satellite locations.

## *Drosophila simulans* centromeric *G2/Jockey-3*

A repeat with 500-bp single unit identified by Talbert et al. [1] as enriched in CENP-A has homology to the first 487 bp of the *D. simulans Jockey-3* annotation in Repbase. However, no other Drosophila species *Jockey-3* element contains this 500-bp fragment, including *G2/Jockey-3* in *D. melanogaster*. We suspect that the Repbase consensus is misannotated. The chimeric annotation may come from *Jockey-3* elements having an insertion preference for the 500-bp satellite, or selection for centromeric insertions of the TE. We cannot distinguish between these possibilities, but a detailed assembly-based method is forthcoming. We therefore refer to this element, less the first 487-bp found in the Repbase annotation [15] (https://www.girinst.org/protected/repbase_extract.php?access=Jockey-3_DSim; last accessed 1/15/2019), as *G2/Jockey-3* and use the *D. simulans* consensus of this element for read mapping in Fig 6.

**S1 Text References**

1. Talbert P, Kasinathan S, Henikoff S. Simple and Complex Centromeric Satellites in Drosophila Sibling Species. Genetics. 2018. Epub 2018/01/07. doi: 10.1534/genetics.117.300620. PubMed PMID: 29305387.

2. Raychaudhuri N, Dubruille R, Orsi GA, Bagheri HC, Loppin B, Lehner CF. Transgenerational propagation and quantitative maintenance of paternal centromeres depends on Cid/Cenp-A presence in Drosophila sperm. PLoS Biol. 2012;10(12):e1001434. Epub 2013/01/10. doi: 10.1371/journal.pbio.1001434. PubMed PMID: 23300376; PubMed Central PMCID: PMCPMC3531477.

3. Lee H, McManus CJ, Cho DY, Eaton M, Renda F, Somma MP, et al. DNA copy number evolution in Drosophila cell lines. Genome Biol. 2014;15(8):R70. doi: 10.1186/gb-2014-15-8-r70. PubMed PMID: 25262759; PubMed Central PMCID: PMCPMC4289277.

4. Jain D, Baldi S, Zabel A, Straub T, Becker PB. Active promoters give rise to false positive 'Phantom Peaks' in ChIP-seq experiments. Nucleic Acids Res. 2015;43(14):6959-68. doi: 10.1093/nar/gkv637. PubMed PMID: 26117547; PubMed Central PMCID: PMCPMC4538825.

5. Chen CC, Dechassa ML, Bettini E, Ledoux MB, Belisario C, Heun P, et al. CAL1 is the Drosophila CENP-A assembly factor. J Cell Biol. 2014;204(3):313-29. doi: 10.1083/jcb.201305036. PubMed PMID: 24469636; PubMed Central PMCID: PMCPMC3912524.

6. Erhardt S, Mellone BG, Betts CM, Zhang W, Karpen GH, Straight AF. Genome-wide analysis reveals a cell cycle-dependent mechanism controlling centromere propagation. J Cell Biol. 2008;183(5):805-18. doi: 10.1083/jcb.200806038. PubMed PMID: 19047461; PubMed Central PMCID: PMCPMC2592830.

7. Olszak AM, van Essen D, Pereira AJ, Diehl S, Manke T, Maiato H, et al. Heterochromatin boundaries are hotspots for de novo kinetochore formation. Nat Cell Biol. 2011;13(7):799-808. doi: 10.1038/ncb2272. PubMed PMID: 21685892.

8. Abad JP, Villasante A. Searching for a common centromeric structural motif: Drosophila centromeric satellite DNAs show propensity to form telomeric-like unusual DNA structures. Genetica. 2000;109(1-2):71-5. PubMed PMID: 11293798.

9. Garavis M, Mendez-Lago M, Gabelica V, Whitehead SL, Gonzalez C, Villasante A. The structure of an endogenous Drosophila centromere reveals the prevalence of tandemly repeated sequences able to form i-motifs. Sci Rep. 2015;5:13307. doi: 10.1038/srep13307. PubMed PMID: 26289671; PubMed Central PMCID: PMCPMC4542561.

10. Kasinathan S, Henikoff S. Non-B-Form DNA Is Enriched at Centromeres. Mol Biol Evol. 2018;35(4):949-62. doi: 10.1093/molbev/msy010. PubMed PMID: 29365169; PubMed Central PMCID: PMCPMC5889037.

11. Chang C-H, Chavan A, Palladino J, Wei X, Martins NMC, Santinello B, et al. Data from: Islands of retroelements are major components of Drosophila centromeres. Dryad Digital Repository. 2019. doi: Openly available via <https://doi.org/10.5061/dryad.rb1bt3j>.

12. Kofler R, Betancourt AJ, Schlotterer C. Sequencing of pooled DNA samples (Pool-Seq) uncovers complex dynamics of transposable element insertions in *Drosophila melanogaster.* PLoS Genet. 2012;8(1):e1002487. doi: 10.1371/journal.pgen.1002487. PubMed PMID: 22291611; PubMed Central PMCID: PMCPMC3266889.

13. Wang L, Dou K, Moon S, Tan FJ, Zhang ZZ. Hijacking Oogenesis Enables Massive Propagation of LINE and Retroviral Transposons. Cell. 2018;174(5):1082-94 e12. doi: 10.1016/j.cell.2018.06.040. PubMed PMID: 30057117.

14. Benjamini Y, Hochberg Y. Controlling the False Discovery Rate - a Practical and Powerful Approach to Multiple Testing. J Roy Stat Soc B Met. 1995;57(1):289-300. PubMed PMID: WOS:A1995QE45300017.

15. Bao W, Kojima KK, Kohany O. Repbase Update, a database of repetitive elements in eukaryotic genomes. Mob DNA. 2015;6:11. Epub 2015/06/06. doi: 10.1186/s13100-015-0041-9. PubMed PMID: 26045719; PubMed Central PMCID: PMCPMC4455052.
